# Flow-Guided Chemical Language Modeling for Linker Design in Reticular Chemistry

**DOI:** 10.1021/jacs.6c06920

**Published:** 2026-09-08

**Authors:** Dhruv Menon, Vivek Singh, Xu Chen, Mohammad Reza Alizadeh Kiapi, Ivan Zyuzin, Hamish W. MacLeod, Nakul Rampal, William Shepard, Omar M. Yaghi, David Fairen-Jimenez

**Affiliations:** † The Adsorption & Advanced Materials Laboratory (A^2^ML), Department of Chemical Engineering & Biotechnology, 2152 University of Cambridge, Cambridge CB3 0AS, U.K.; ‡ Department of Chemistry, 12442 University of California, Berkeley, California 94720, United States; § Bakar Institute of Digital Materials for the Planet, College of Computing, Data Science, and Society, University of California, Berkeley, California 94720, United States; ∥ KACST−UC Berkeley Center of Excellence for Nanomaterials for Clean Energy Applications, King Abdulaziz City for Science and Technology, Riyadh 11442, Saudi Arabia; ⊥ Milner Therapeutics Institute, Jeffrey Cheah Biomedical Centre, Puddicombe Way, Cambridge CB2 0AW, U.K.; # Synchrotron SOLEIL-UR1, L’Orme des Merisiers, Départementale 128, Saint-Aubin 91190, France

## Abstract

Reticular chemistry has enabled the synthesis of tens of thousands of metal–organic frameworks (MOFs), yet the discovery of new materials still relies largely on intuition-driven linker design and iterative experimentation. As a result, researchers explore only a small fraction of the vast chemical space accessible to reticular materials, limiting the systematic discovery of frameworks with targeted properties. Here, we introduce Nexerra^R1^, a building-block chemical language model that enables inverse design in reticular chemistry through targeted generation of organic linkers. Rather than generating complete frameworks directly, Nexerra operates at the level of molecular building blocks, preserving the modular logic that underpins reticular synthesis. The model supports both unconstrained generation of low-connectivity linkers and scaffold-constrained design of symmetric multidentate motifs compatible with predefined nodes and topologies. We further combine linker generation with flow-guided distributional targeting to steer the generative process toward application-relevant objectives while maintaining chemical validity and assembly feasibility. The generated linkers are subsequently assembled into three-dimensional frameworks and structurally optimized to produce candidate materials compatible with experimental synthesis. Using Nexerra^R1^, we validate this strategy by rediscovering known MOFs and by proposing the experimental synthesis of a previously unreported framework, CU-525, generated *in silico*. Together, these results establish a controllable building-block-level design framework for reticular chemistry in which chemical language modeling enables the direct translation from computational design to synthesizable frameworks.

## Graphical abstract

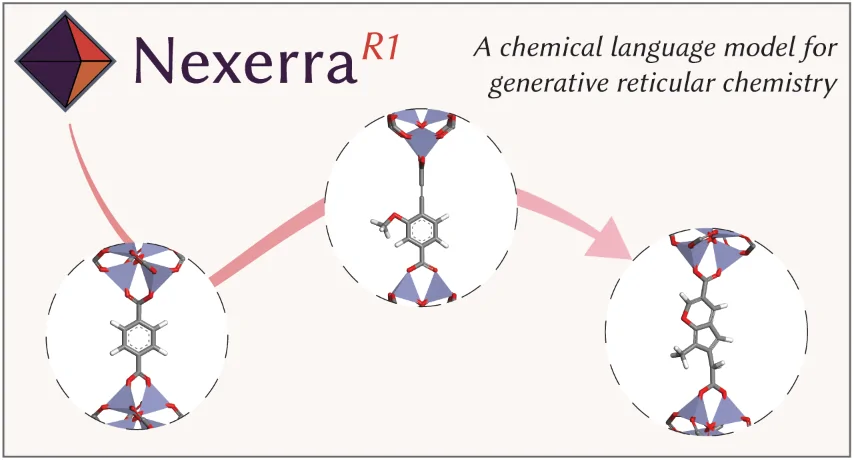

## 1 Introduction

The geometry-guided design and assembly of periodically extended structures through reticular chemistry have transformed the discovery of porous materials, enabling breakthroughs in areas such as carbon capture^1–3^ and water harvesting.^4^ Over the past three decades, researchers have discovered most metal–organic frameworks (MOFs) through chemical intuition-driven experimentation. While this approach has delivered remarkable materials with exceptional properties, progress has remained largely empirical and slow, limiting the systematic exploration of the vast chemical space accessible to MOFs and their rapid translation into industrial applications.^5–7^ Computational methods have begun to address these challenges. Classical molecular simulations and machine learning (ML) approaches now allow researchers to screen large libraries of materials *in silico*, expanding the search space and reducing experimental effort.^8,9^ Despite these advances, the current strategies remain fundamentally constrained. High-throughput screening still explores only a tiny fraction of the enormous design space of MOFs and relies entirely on known structures or, at best, combinatorially assembled hypothetical frameworks. As a result, current approaches struggle to guide the discovery of genuinely new materials that are designed from the outset to exhibit target properties.

In this context, generative modeling offers a promising route toward the inverse design of MOFs with targeted properties. These approaches learn the underlying statistical structure of chemical data sets and use this knowledge to generate new candidates that satisfy predefined objectives. Several generative approaches have recently emerged, including chemical language models, score-based models, and denoising diffusion probabilistic models (DDPMs), which have shown considerable success across molecular design problems such as small-molecule generation,^10^ drug discovery,^11^ *de novo* protein design,^12^ reaction mechanism prediction,^13^ and inorganic materials design.^14^ Among these approaches, chemical language models (CLMs) have attracted particular attention. These models treat molecular representations as sequences and learn to predict the probability of the next token given the preceding context.^15^ By learning the statistical patterns embedded in larger chemical data sets, CLMs can generate entirely new molecules consistent with the training distribution.^16^ When trained on massive data sets, these architectures scale into large language models (LLMs), which can capture increasingly complex chemical relationships.^15^ Other generative strategies have also proven to be powerful. Diffusion-based approaches, such as DDPMs, iteratively denoise data corrupted with noise, generating highly diverse outputs of interest.^17^ Score-based models provide a closely related formulation in which neural networks estimate gradients of the logarithmic probability density (known as the score) of noise-corrupted data.^18^ Despite this rapid progress, applying generative modeling to reticular chemistry remains a challenge. Many existing approaches struggle to ensure synthetic feasibility, adapt to the modular design principles of reticular materials, or generate structures that remain compatible with experimentally accessible building blocks and topologies. As a result, significant opportunities remain to develop generative strategies that directly address the unique constraints of reticular synthesis and enable the targeted discovery of new MOFs.^19–22^

Prior generative approaches for reticular materials largely formulated MOF design as an optimization within predefined “reticular vocabularies” of known topologies, metal nodes, and components.^20^ In these approaches, framework assembly remained constrained to discrete libraries of experimentally established reticular building blocks, with exploration generally restricted to combinations of these building blocks rather than the direct generation of novel linker chemistries. An early building-block representation for reticular materials was MOFid,^23^ which decomposes MOFs into their constituent building blocks and underlying topology to generate language-like identifiers. This representation subsequently inspired higher-level encodings, such as RFcode,^20^ that enabled framework-level inverse design within predefined reticular component libraries. More recently, generative frameworks, including Building-Block-Aware MOF Diffusion^24^ and EGMOF,^25^ have extended this paradigm toward the generation of complete framework structures by explicitly modeling reticular building blocks and topology. These advances represent important progress toward autonomous framework generation. However, the simultaneous generation of chemically valid building blocks, topologically consistent frameworks, and target properties remains an inherently high-dimensional inverse-design problem.

We therefore adopt a complementary perspective by decoupling the molecular design from framework assembly. Although the chemically flexible degree of freedom is primarily the linker (or edge representation), designing linkers remains challenging because they must simultaneously satisfy the geometric, symmetry, and coordination constraints required for the framework assembly. In parallel, most molecular generative models have been developed for pharmaceutical small molecules, which occupy a fundamentally different chemical space characterized by smaller molecular sizes, medicinal-chemistry-relevant functionalities, and comparatively less rigid structural constraints. MOF linkers instead occupy a more structured region of the chemical space defined by multidentate coordination motifs, extended rigidity, symmetry constraints, and structural assembly requirements. Unlike conventional molecular generation, linker design therefore requires not only chemical validity but also explicit compatibility with a predefined reticular environment. These characteristics pose challenges for fully atomistic generative approaches, particularly diffusion models, where preserving long-range geometric consistency becomes increasingly difficult with the linker size.^26^ We, therefore, view reticular linker design as operating at the intersection of molecular generative modeling and reticular framework design approaches, requiring both latent space navigability and structural constraints.

This approach mimics experimental practice, where synthetic chemists rationally select or design molecular building blocks with prespecified geometries to assemble desired nets.^27^ Here, we introduce Nexerra^R1^, a flow-guided CLM that generates diverse, synthesizable organic linkers for reticular chemistry. Nexerra^R1^ generates linkers that can be steered toward application-relevant molecular properties while preserving reticular compatibility. Nexerra^R1^ supports both the unconstrained design of bidentate and tridentate linkers, and the scaffold- or functional motif-constrained design of symmetric, multidentate linkers (e.g., porphyrin- and pyrene-derived linkers). Under the formulation of flow matching^28,29^ —a generalization of the diffusion process—in an optimal transport (OT) setting,^29^ Nexerra^R1^ can be steered toward target molecular distributions relevant to specific design objectives. By combining theory-informed reward functions with design templates, Nexerra^R1^ can guide the design of high-performing candidates that are compatible with experimental synthesis.

## 2 Results and Discussion

### 2.1 Model Architecture

Designing new MOFs often begins at the level of molecular building blocks.^27^ In reticular chemistry, frameworks arise from the assembly of inorganic nodes and organic linkers into periodic nets whose geometry determines the resulting structure (Figure 1a). To make the design space tractable and ensure reliable targets, we focus on edge-transitive nets (i.e., nets with a single type of nonintersecting edge) and zero-periodic metal-cluster building blocks (MBBs), which are experimentally robust, yet span diverse coordination geometries (see Section S1 for representative nets and nodes).^27,30^ Linkers offer a vast design space, where subtle structural changes can induce major effects on MOF structure and function. Nexerra^R1^ uses a textual representation of linkers and learns a continuous latent space for inverse linker design using a β-variational autoencoder (β-VAE)^31^ as a probabilistic generative model (for the model architecture, see Section S2). To enable this representation, we encode linkers using SELFIES, a molecular string language with a formal grammar that guarantees chemical validity (Section S2.1).^32^

**Figure 1:**
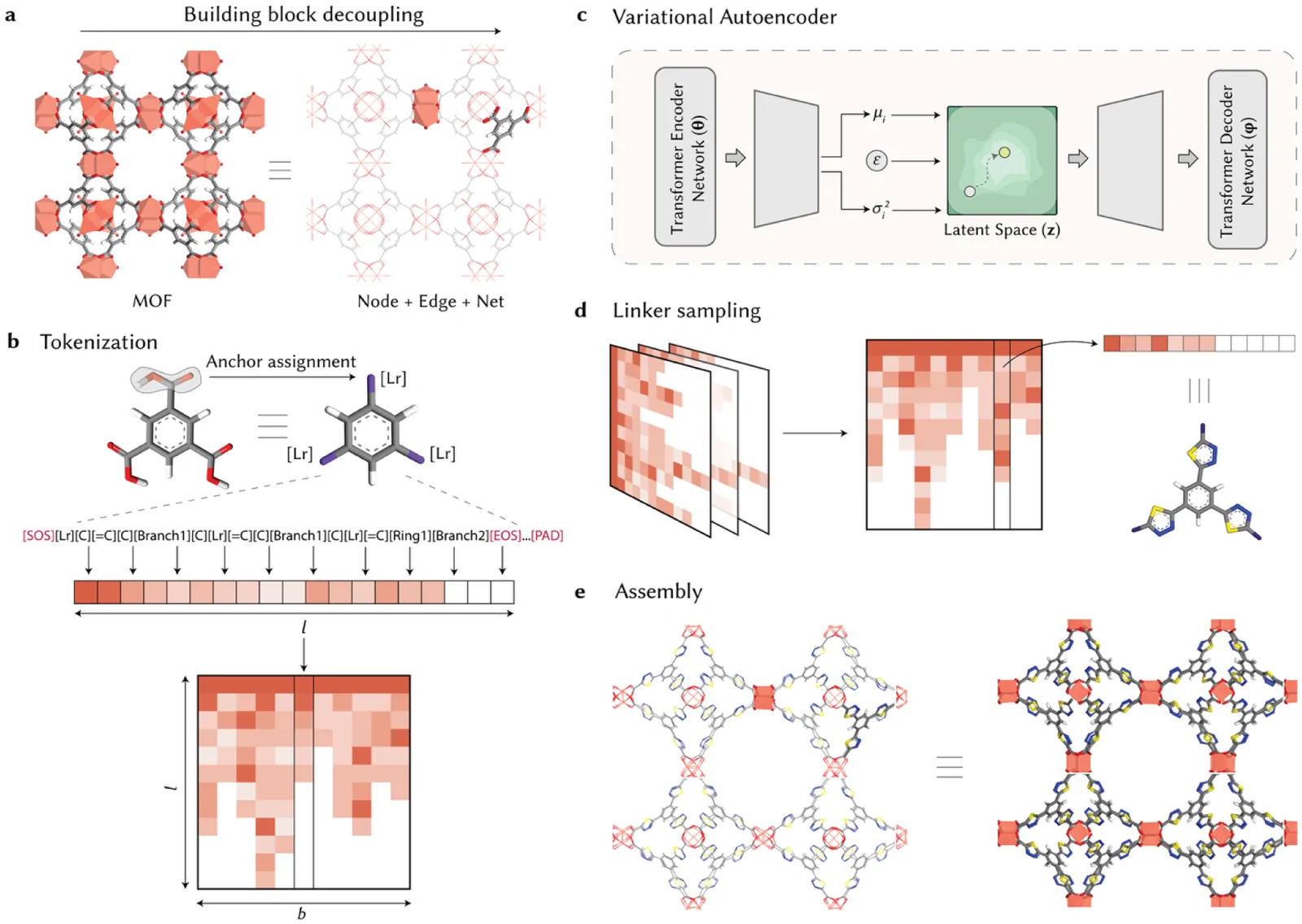
Inverse reticular chemistry with Nexerra^R1^. **a.** MOF structures are represented as nets made of nodes and edges. As a first step, we decouple the design process at the building-block level. **b.** Linkers are encoded as molecular strings (SELFIES) and tokenized into a sequence of symbols. Each token acts as a structural unit that is processed by the model. **c.** The VAE acts as a bottleneck wherein the encoder defines an approximate posterior over a low-dimensional, continuous latent variable (z), while the decoder reconstructs the original sequence from z. **d.** Nucleus sampling is used to generate new linkers from the decoder output. **e.** Subject to the synthetic feasibility of the designed linker, an attempt is made to assemble it into the target net. (A detailed theoretical description of the model is available in Section S2. Figure S1 describes the encoder and decoder networks. Color code: Polyhedron—Cu_2_(μ-COO)_4_; Gray—C; Red—O; Blue—N; Yellow—S; White—H; Purple—metal-linker coordination motif).

Each SELFIES string is then tokenized, with each token acting as a structural unit processed by a neural network (Figure 1b). The encoder network approximates a posterior *q*_ϕ_(*z*|*x*) over a low-dimensional, continuous latent variable *z*; the decoder specifies a likelihood *p*_θ_(*x*|*z*) that reconstructs the original sequence from *z* (Figure 1c). The model is trained by maximizing a β-weighted evidence lower bound (ELBO), balancing accurate input reconstruction against latent distribution regularization (for the mathematical formulation, see Section S2.2). This continuous latent space enables efficient sampling, smooth exploration, and controlled perturbation of the linker chemical space for inverse design.^33^

The encoder and decoder are implemented as stacks of transformer layers (outlined in Figure S1), transforming a set of vectors from one representation space to another while learning a rich internal representation.^34^ Through the attention mechanism,^35^ which assigns input-dependent weights to different elements of the sequence, capturing long-range dependencies, molecular strings are mapped to chemically meaningful embeddings that support generative design in the latent space (Section S2.3). Specifically, the encoder contextualizes the input token embeddings and compresses them into a latent representation through the self-attention mechanism, while the decoder projects this representation back into the input space using a combination of masked self-attention and cross-attention mechanisms (Section S2.3). The decoder outputs a probability distribution over the next token in the generated sequence, which we sample to generate new linkers (Figure 1d, Section S2.4). Following postprocessing, where the placement of coordination moieties is optimized (optimization algorithm described in Section S5), the linkers are subsequently assembled along with the node on a chosen net template to construct a new MOF (Figure 1e, Section S5).

Nexerra^R1^ is trained on a corpus of approximately 900,000 linkers derived from existing databases^20^ and systematically functionalized to expand chemical diversity (for data set curation, see Section S3). To maintain synthetic plausibility, we applied chemistry-based filters to remove linkers with high synthetic complexity, quantified by the synthetic complexity score (SCScore).^36^ The resulting data set spans a chemically rich, nonredundant linker space biased toward synthetically accessible chemotypes (Section S3). Nexerra^R1^ achieves per-token reconstruction accuracy of 96.5%, internal diversity >0.925, and novelty >96%, consistent with a nondegenerate latent representation of MOF linker space (for training and evaluation, see Section S4). Across a held-out test set, Nexerra^R1^ preserves the overall physicochemical profile of MOF linkers, reproducing descriptors related to molecular polarity (TPSA), hydrophobicity (logP), size (molecular weight), and structural complexity (aromaticity/ring counts). In particular, the model closely matches TPSA and aromatic ring distributions while showing only modest biases toward higher logP and molecular weight (Figure S7). Under unconstrained sampling, Nexerra^R1^ proposes diverse, multifunctional linkers with extended aromatic cores and dense substitution patterns consistent with the expressive capacity of the architecture (see example linkers in Figure S9). However, unconstrained generation alone does not guarantee an efficient “hit rate” for application-relevant candidates. We, therefore, employ templated, seed-guided design to focus sampling on promising regions of linker space and improve the efficiency of the inverse linker design process.

### 2.2 Linker Design and MOF Synthesis

Unconstrained sampling draws latent vectors z from the prior *p*(*z*) and decodes them autoregressively into new linkers, enabling global exploration of the learned linker distribution. In practice, however, only a small fraction of such samples remain compatible with the geometric constraints imposed by a chosen node-net template and with application-driven design objectives. We, therefore, use Nexerra^R1^ as a controllable generator via seeded inference: we encode a known linker (x_0_) into a local latent distribution *q*_ϕ_(*z*|*x*_0_), sample the neighborhood of its embedding, and decode analogues that preserve the seed chemotype while exploring nearby linker space. We couple this localized sampling to post hoc ranking under user-defined reward functions (denoted **R**), retaining the highest-scoring candidates $\left(X^{*}=TopK\left(x_{i},R\right)\right)$ . Section S6 shows the theoretical basis and formulation of these functions. Importantly, the generative model is not updated by the reward; **R** is used solely to select designs.

We implement seeded inference in two complementary modes (Figure 2a, Section S7). In “direct design”, the entire seed (x_0_) is allowed to vary. From the encoded neighborhood of x_0_, we generate a local library {x_k_}, assemble each linker on the chosen reticular template, and retain the *TopK* candidates under **R**. This mode is most effective for low connectivity, i.e., in systems with constrained connectivity, where the node geometry and linker connectivity largely determine topology. Using MOF-5 as a template and 1,4-benzenedicarboxylate (BDC) as a seed, we obtained several linkers satisfying **R**_**gas**_ > 0.75 × **R**_**gas**_ (seed) – **R**_**gas**_ being a function formulated for identifying exceptional linkers for gas storage (derived in Section S6). We could assemble nearly all candidates on the **pcu** net, yielding multiple clean, force-field-optimized frameworks (details in Section S10.1). Figure 2b shows 6 such designed MOFs (ϕ_1_ – ϕ_6_) after cleaning and optimization; Section S7.1 shows the direct design approach applied to other classes of bidentate and tridentate linkers.

**Figure 2:**
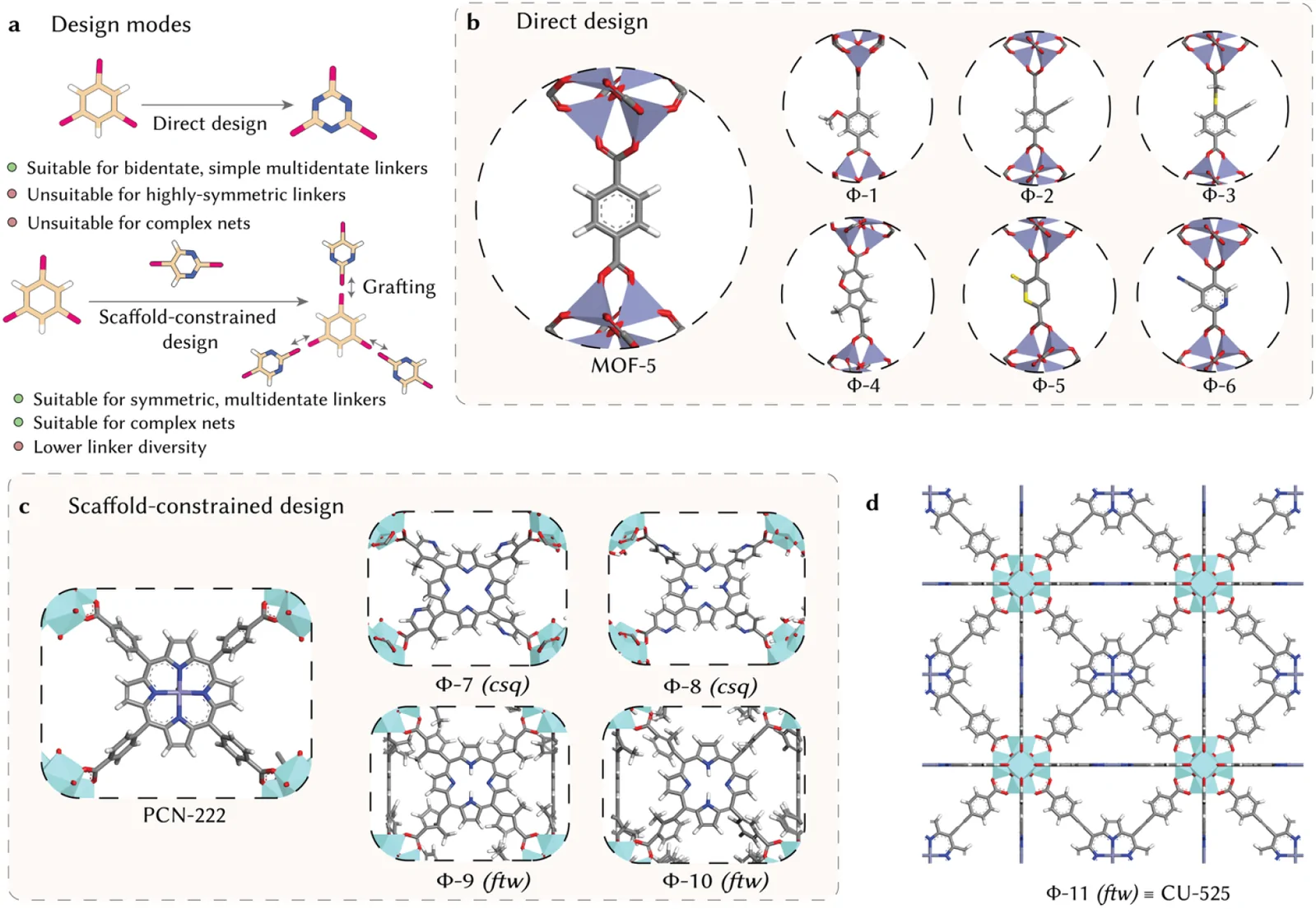
Linker design. **a.** Nexerra^R1^ enables inference in two modes: direct design and scaffold-constrained design. In direct design, Nexerra^R1^ fully maps the seed to new linkers, while in scaffold-constrained design, the scaffold is fixed, and the model generates new arms that are symmetrically grafted to the core. **b.** Using MOF-5 as a design template, Nexerra^R1^ generates a library of linkers which are filtered and scored by a reward function (**R**). ϕ_1_ – ϕ_6_ are 6 representative MOFs that were successfully assembled with the Zn_4_O node on the **pcu** net. **c.** Using PCN-222 as a design template, ϕ_7_ – ϕ_10_ are 4 representative MOFs that were assembled with the Zr_6_-oxo node. Since several topologies can be targeted (depending on geometric and topological constraints), we were able to successfully assemble candidates into the 8-connected **csq** net and the 12-connected **ftw** net. **d.** ϕ_11_ assembled on an **ftw** net was experimentally synthesized as CU-525. (Color code: Polyhedron (Purple)—Zn_4_O­(COO)_6_; Polyhedron (Blue)—Zr_6_(μ_3_-O)_4_(μ_3_-OH)_4_(COO)_8_(OH/H_2_O)_4_; Gray—C; Red—O; Blue—N; Yellow—S; White—H).

For high-connectivity frameworks, direct design becomes unreliable because small perturbations in the latent space can distort rigid, highly symmetric scaffolds that are essential for preserving topology. We, therefore, introduce a second design strategy, “scaffold-constrained design” (Figure 2a), in which the linker is decomposed as *x* = (*s,a*), where *s* represents a fixed core scaffold and *a* represents variable arms. During generation, we preserve the scaffold and sample only the arms, allowing Nexerra^R1^ to explore functionalization and pore-environment chemistry while preserving the connectivity and geometry required for framework assembly. We apply this strategy to porphyrinic Zr_6_-oxo systems using PCN-222 as a template. Starting from the tetrakis­(4-carboxyphenyl)­porphyrin (TCPP) linker, Nexerra^R1^ diversifies linker chemistry while retaining the porphyrin core, enabling targeted modulation of the pore environment without violating topology-critical geometry (Figure 2c). Among the top-ranked candidates (filtered using **R**_**grav**_ and **R**_**gas**_), several could be assembled and optimized on the **csq** and **ftw** nets. Figure 2c shows four such designed MOFs (ϕ_7_ – ϕ_10_); Section S7.2 shows this approach applied to other linkers. Notably, the Hf_6_-oxo analogue of one such **ftw** design was synthesized as CU-525 (CU = Cambridge University), a variant of MOF-525 (Figure 2d). This result demonstrates that scaffold-constrained generative design can yield synthetically accessible, highly porous frameworks whose internal chemistry can be tailored for function. Single-crystal X-ray diffraction (SCXRD) data were collected, and the crystal structure of CU-525 was successfully determined on the microfocus beamline PROXIMA 2A at Synchrotron SOLEIL (for experimental details, see Section S8). Although this seeded approach concentrates sampling relative to unconstrained generation, it still relies on random exploration followed by ranking. The learned prior reflects the empirical distribution of the training data rather than the structural requirements of a specific application. Consequently, sampling from this prior—even when guided by seeds—remains inefficient when targeting linkers with specific geometric or physicochemical attributes.

### 2.3 Flow-Guided Distributional Targeting

To address this sampling inefficiency, we developed a flow model—Nexerra^R1^-Flow—that implements a conditional flow-matching model in the latent space of Nexerra^R1^ (Figure 3a).^29^ The flow defines a time-dependent, invertible transformation by learning a vector field *v*_θ_(*z*,*t*;*c*) that transports latent samples (*z*) from a base distribution *p*_0_ to a target distribution *q*_1_; conditioning is introduced using a scalar variable *c*.^28,29^ Nexerra^R1^-Flow is trained using optimal-transport conditional flow matching (OT-CFM); an optimal transport coupling (π) is computed between minibatches from *p*_0_ and *q*_1_, and intermediate points *x_t_* together with conditional velocities *v_t_* are sampled along the corresponding displacement interpolation path. A neural vector field is then trained to match these velocities and integrated as an ordinary differential equation (ODE) during inference, allowing probability mass to be redistributed toward the target regime without modifying the underlying CLM (for the mathematical formulation, see Section S9.1). This flow formulation is, in principle, reward-agnostic; *q*_1_ can be biased toward arbitrary scalar objectives relevant to reticular materials design. The difficulty of this transport, however, reflects the intrinsic geometry of the objective in the latent space. Fragmented or nonsmooth reward landscapes produce more complex transport dynamics—making the training process more difficult.

**Figure 3:**
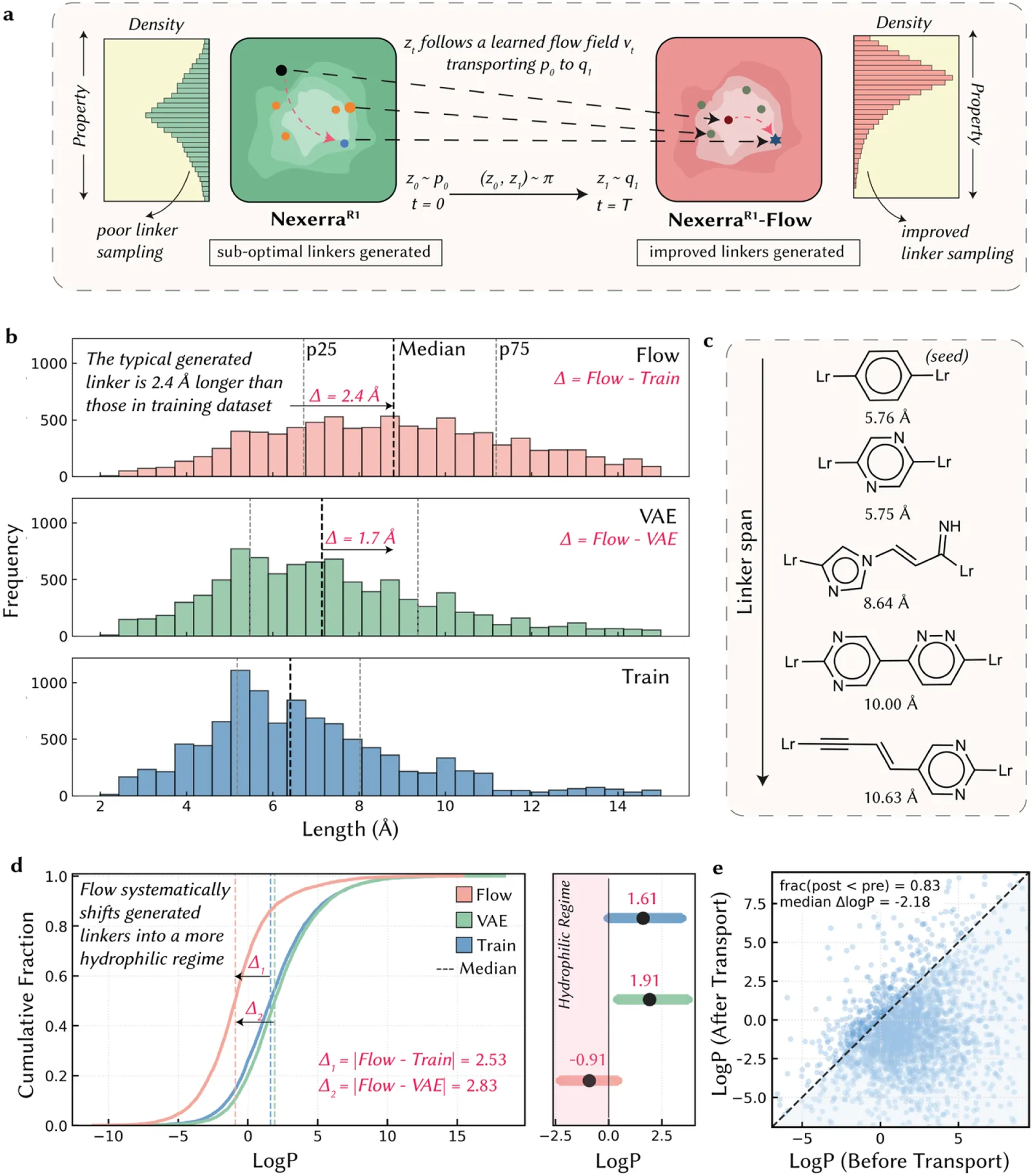
Flow-guided design. **a.** Nexerra^R1^-Flow learns a vector field *v*_θ_ *(z*,*t*;*c*) that transports latent samples from a base distribution *p*_0_ to a target distribution *q*_1_ under the formulation of optimal-transport conditional flow matching (a detailed theoretical description of the model is available in Section S9.1). **b.** Linker span distributions of molecules sampled from the training data set (*n* = 10,000), Nexerra^R1^ (*n* = 10,000), and Nexerra^R1^-Flow (*n* = 10,000). The typical generated linker from Nexerra^R1^-Flow is 2.4 Å longer compared to the training data set and 1.7 Å longer compared to Nexerra^R1^. **c.** Using BDC as the seed, Nexerra^R1^-Flow generates several optimal linkers with a longer span; the best-ranked linker has a span of 10.63 Å which is 1.8× the span of the seed. The corresponding generated structures are presented in Section S9.4. **d.** Cumulative LogP distributions of molecules sampled from the training data set (*n* = 10,000), Nexerra^R1^ (*n* = 10,000), and a separately trained Nexerra^R1^-Flow model targeting lower LogP (*n* = 9617). Flow-guided transport systematically shifts the generated linker population to a more hydrophilic regime, with the median LogP reducing relative to both the training data set and the Nexerra^R1^ generated population. **e.** A molecule-wise comparison of LogP values before and after latent-space transport (*n* = 2499). 83% of linkers undergo a reduction in LogP following transport, consistent with property-steering toward a more hydrophilic regime.

The linker length (defined by us as the maximum anchor–anchor span) presents a physically interpretable descriptor. Increasing the span helps maximize accessible pore volume, lending itself to applications such as improved gravimetric gas storage and heterogeneous catalysis.^37–39^ Starting from an empirical latent prior *p*_0_, we train Nexerra^R1^-Flow toward a target distribution biased toward longer spans by progressively increasing percentile thresholds. Specifically, over 200,000 steps, the target region was annealed from the 70th to the 95th percentile of the anchor–anchor span distribution (for training and evaluation, see Section S9.3). This progressively biases generation toward longer linker spans. Figure 3b shows span distributions of the linkers sampled from the training data set, Nexerra^R1^, and Nexerra^R1^-Flow, respectively. While Nexerra^R1^ reproduces the marginal distributions of the training data set (KL­(Train || VAE) ∼ 0.10), there is a marked shift in the distribution generated by the flow model (KL­(Train || Flow) ∼ 0.42). Notably, the median length sampled from Nexerra^R1^-Flow is right-shifted by 2.4 Å compared to the training data set and by 1.7 Å compared to Nexerra^R1^. There is a large population of generated linkers that exceed 10 Å in span. As an illustrative example, using the BDC linker as a seed, Nexerra^R1^-Flow generated several linkers that outperform it pursuant to an **R** that prioritizes length. Figure 3c shows several optimal candidates generated by Nexerra^R1^-Flow subject to strict geometrical filters to enable assembly into the **fcu** net; Figure S22 shows the generated structures postoptimization. Notably, Nexerra^R1^-Flow was able to scale the linker span from 5.76 Å for the seed to over 10.6 Å for the best generated candidate. Unlike random functionalization or enumeration-based analogue generation strategies, which primarily explore highly local chemical neighborhoods surrounding a seed linker, latent-space transport enables controllable traversal toward chemically diverse linker populations. Section S9.5 compares Nexerra^R1^-Flow against constrained enumeration-based baselines and illustrates how progressively stronger transport strengths systematically shift the accessible linker distribution beyond local substituent-modification regimes. To further evaluate whether the learned latent-space transformation remains chemically meaningful, in Section S9.6 we performed a rediscovery analysis using representative linkers including BDC and biphenyl-4,4″-dicarboxylic acid (BPDC; UiO-67). Nexerra^R1^-Flow recovered several experimentally reported linker families exactly, including BDC, BPDC, 4,4″-dicarboxy-*p*-terphenyl (TPDC; UiO-68), and 9,10-anthracenyl bis­(benzoic acid) (DPA; DPA-MOF),^40^ while also generating structurally similar analogues that preserve structural constraints such as anchor symmetry and compatible linker geometries (Figure S25).

To probe the generality of the flow-guided latent-transport method, we next trained a separate Nexerra^R1^-Flow model, targeting lower linker hydrophobicity through minimization of the octanol/water partition coefficient (logP). LogP is a widely used descriptor for molecular hydrophobicity, with increasingly positive values corresponding to greater lipophilicity (hydrophobicity) and increasingly negative values corresponding to greater hydrophilicity. Whereas the previous linker length example biases generation toward larger architectures, many applications additionally depend on the chemical character of the pore environment. For example, water-harvesting applications require optimizing several (often competing) textural properties (e.g., pore size and pore volume) and surface chemistry.^41^ We therefore selected logP as an objective to test whether latent-space transport can steer populations to physicochemical distributions of interest.

Figure 3d shows the cumulative logP distributions of linkers sampled from the training data set, Nexerra^R1^, and Nexerra^R1^-Flow. Flow-guided transport produces a clear redistribution of the linker population toward more hydrophilic regimes. Figure 3e shows a molecule-wise comparison of logP values before and after latent-space transport, with the majority of linkers undergoing a reduction in logP following transport. Importantly, both objectives are achieved by using the same underlying latent transport formulation without retraining the generative prior itself. While one flow field transports the latent distribution toward longer linker geometries, the second transports it toward chemically distinct hydrophilic regimes, demonstrating that the framework can flexibly steer generation toward different application-relevant molecular objectives.

### 2.4 Application-Guided Design

To evaluate whether flow-guided latent transport translates into application-relevant performance gains, we simulated methane adsorption at 298 K and 80 bar using grand canonical Monte Carlo (GCMC) simulations on the optimized frameworks assembled on the **fcu** net from the designed linkers discussed in the previous section (for computational details, see Section S10). Methane storage in porous materials is known to be strongly influenced by structural descriptors such as accessible surface area, pore volume, and framework density.^42^ Accordingly, the present objective is to evaluate whether application-guided linker generation can identify chemically novel candidates that improve framework-level performance within fixed reticular-assembly constraints. Importantly, linker elongation can potentially be accompanied by competing trade-offs. While increasing the linker span can enhance accessible pore volume, excessively long linkers may promote framework interpenetration (depending on the topology), increase conformational flexibility, and compromise mechanical robustness during activation.^43,44^ Similarly, synthetic tractability can also pose challenges for increasingly long and complex linkers. To partially account for these effects, the underlying reward formulation for prioritizing linkers does not rely on the linker span alone but instead balances several chemical descriptors relevant to gas storage performance, including rigidity, linker symmetry, and molecular-weight-dependent packing considerations (Section S6.1). In particular, flexibility penalties based on rotatable bond counts and sp^3^ character were introduced to bias generation toward more rigid, shape-persistent linkers, with bounded desirability functions to prevent extreme characteristics. Figure 4 presents the gravimetric versus volumetric methane uptake landscape of the generated frameworks relative to UiO-66. The structures were able to achieve a systematic shift of uptakes toward the upper-right region of the performance space; in other words, we were able to achieve a concurrent increase in both volumetric and gravimetric uptake—arguably, the two critical and competing metrics within a fixed topology.^37,38^ While UiO-66 occupies a relatively moderate capacity (gravimetric uptake, 0.102 g/g; volumetric uptake, 173 cm^3^ (STP)/cm^3^), several generated MOFs outperform it. The two best-performing candidates—ϕ_13_ and ϕ_23_—achieve gravimetric uptakes of 0.191 g/g and 0.179 g/g and volumetric uptakes of 203 cm^3^ (STP)/cm^3^ and 219 cm^3^ (STP)/cm^3^, respectively. These represent up to an 87% increase in gravimetric and a 27% increase in volumetric uptake relative to UiO-66. These results demonstrate that latent-space flow transport can efficiently bias reticular linker generation toward high-performing regions of the constrained design space relative to a seed framework, while preserving fixed topological and assembly constraints.

**Figure 4:**
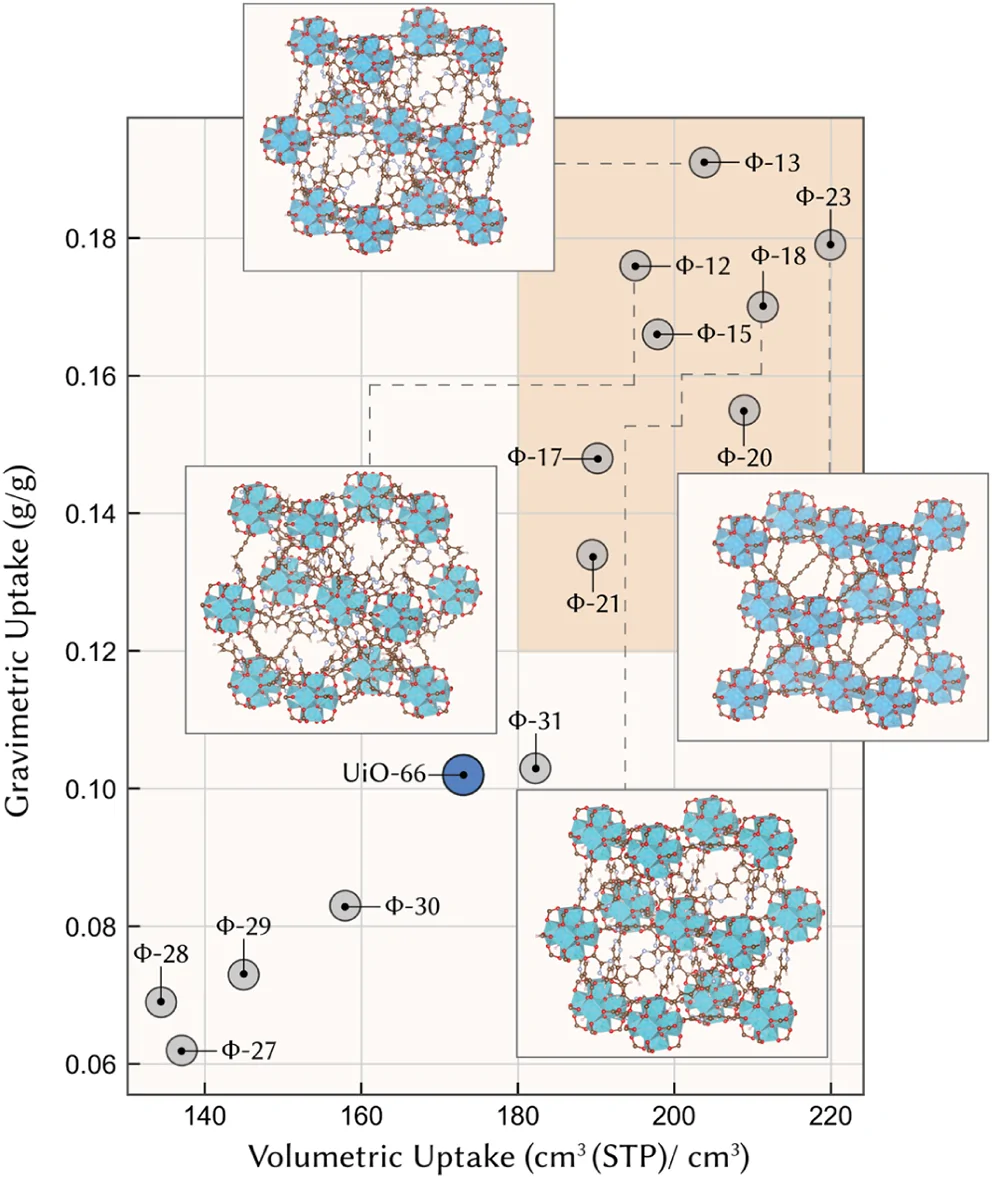
Flow-guided inverse linker design of UiO-66 analogues for methane storage. Gravimetric versus volumetric methane uptake at 298 K and 80 bar calculated using GCMC simulations for **fcu** frameworks assembled from Nexerra^R1^-Flow designed linkers (BDC seed). Several candidates simultaneously exceed UiO-66 in both volumetric and gravimetric uptake. The distributional steering achieved by Nexerra^R1^-Flow enables concurrent improvements in storage performance, as shown in the darker shaded region. Insets show representative high-performing optimized structures. (Color code: Polyhedron—Zr_6_(μ_3_-O)_4_(μ_3_-OH)_4_(COO)_12_; Gray—C; Red—O; Blue—N; Yellow—S; White—H).

In a complementary study, we additionally evaluated methane adsorption in the experimentally synthesized CU-525 framework relative to its originating seed MOF-525. As noted above, the linker was prioritized on the basis of the gravimetric reward function **R**_**grav**_ (Section S6.1). Consistent with its expanded pore architecture, CU-525 exhibited a higher gravimetric methane uptake relative to MOF-525 (0.272 g g^–1^ vs 0.218 g g^–1^ at 298 K and 80 bar). Section S10.3 describes the comparative GCMC analysis, and Figure S26 visualizes representative adsorption snapshots. These simulations provide computational support for the underlying design rationale by demonstrating that the experimentally realized framework exhibits the expected improvement relative to its parent structure under an identical computational protocol. GCMC simulations are well established for predicting methane adsorption in MOFs and therefore provide a robust computational assessment of the proposed design.^45^ However, the present study does not include experimental benchmarking of methane adsorption under identical synthesis, activation, and measurement protocols. Accordingly, while the computational results provide strong support for the proposed design, comprehensive experimental measurements across newly generated frameworks remain an important next step toward closing the inverse-design loop.

Although methane storage serves as the end-to-end application demonstrated in this work, the underlying framework is not restricted to gas storage. Since the design objective only enters through the reward function **R**, alternate application-specific targets can be incorporated without modifying the underlying generative prior. As an illustrative example, building on our recent work on the computational design of biocompatible MOFs for drug delivery,^46,47^ in Section S6.2, we develop a modular reward function **R_bio_** for the design of biocompatible, high-performance linkers. This highlights how the same platform can be adapted to steer generative reticular chemistry to diverse functional regimes. Future work will couple high-ranked designs with experimental synthesis and characterization for a direct assessment of the predictive power of flow-guided latent transport—ultimately, enabling accelerated reticular materials discovery.

## 3 Conclusions

We have introduced a targeted, controllable approach for linker design in reticular chemistry in which chemical language modeling is coupled to latent space transport to bias generation toward functional regimes. The developed formulation is modular and can, in principle, be extended to additional scalar objectives relevant to reticular materials design, including adsorption, stability, or catalytic descriptors. A central implication of our work is the decoupling of representation learning from objective steering. By maintaining a fixed generative prior over the linker space and learning an optimal transport map in latent space, we enable targeted redistribution of probability mass without the need for retraining. This allows application-specific linker design while preserving the structural grammar of the reticular assembly. Although our current implementation operates within predefined node and topology constraints, the conceptual framework establishes a foundation for future extensions toward joint linker-node optimization, enabling the exploration of heterogeneous metal environments and mixed-node systems. More broadly, continued advances in generative modeling, automated property evaluation, and experimental automation are likely to accelerate the systematic exploration of the reticular material space.^48^ In this context, platforms such as Nexerra^R1^ provide a scalable strategy for controllable discovery of new building blocks in constrained structural manifolds. An important future direction will be the prospective experimental validation of computationally prioritized candidates across multiple reticular systems. Integrating generative design with synthesis and adsorption measurements will enable fully closed-loop optimization and provide rigorous experimental assessment of inverse-design frameworks under practical conditions. We anticipate that such platforms will accelerate the transition from intuition-driven synthesis to programmable MOF engineering, where application-specific materials are generated, assembled, and validated within a unified computational-experimental pipeline.

## Supplementary Material



## Data Availability

The crystallography data in this work have been deposited in the Cambridge Crystallographic Data Centre (CCDC) under the accession number CCDC: 2514566 (CU-525). These data can be obtained free of charge from CCDC via www.ccdc.cam.ac.uk/data_request/cif. The code for Nexerra^R1^ is available at https://github.com/fairen-group/nexerra-r1 under an MIT license. Model weights are available at https://zenodo.org/records/19100679 under an MIT license.

## Notes

Model architecture, data set curation and generation, model training and evaluation, postprocessing, reward function formulation, design modes, experimental details, flow model formulation and training, and molecular simulation details (PDF)
