# Supplementary material for: Flow-Guided Chemical Language Modeling for Linker Design in Reticular Chemistry
Source: J Am Chem Soc. 2026 Sep 8;148(37):39546–55. doi: 10.1021/jacs.6c06920 (PMC13614305; doi:10.1021/jacs.6c06920)
Supplement: Supplementary file 1 [file ja6c06920_si_001.pdf]

Supplementary information for

Flow-guided chemical language modeling for linker design in reticular chemistry

Dhruv Menon[1,2], Vivek Singh[2,3,4], Xu Chen[1], Mohammad Reza Alizadeh Kiapi[1], Ivan Zyuzin[1,5], Hamish W. MacLeod[1], Nakul Rampal[2,3,4], William Shepard[6], Omar M. Yaghi[2,3,4] and David Fairen-Jimenez[1,*]

[1]The Adsorption & Advanced Materials Laboratory (A^2ML), Department of Chemical Engineering & Biotechnology, University of Cambridge, Cambridge CB3 0AS, United Kingdom.

[2]Department of Chemistry, University of California, Berkeley, California 94720, United States.

[3]Bakar Institute of Digital Materials for the Planet, College of Computing, Data Science, and Society, University of California, Berkeley, California 94720, United States.

[4]KACST–UC Berkeley Center of Excellence for Nanomaterials for Clean Energy Applications, King Abdulaziz City for Science and Technology, Riyadh 11442, Saudi Arabia.

[5]Milner Therapeutics Institute, Jeffrey Cheah Biomedical Centre, Puddicombe Way, Cambridge CB2 0AW, United Kingdom.

[6]Synchrotron SOLEIL-UR1, L'Orme des Merisiers, Départementale 128, 91190 Saint-Aubin, France.

Correspondence: df334@cam.ac.uk

Table of Contents

S1. Edge-transitive nets and compatible nodes ………………………….. **2**
S2. Nexerra[R1] architecture ……………………………………………….. **3**
S3. Dataset curation and generation ……………………………………… **6**
S4. Model training and evaluation …………………………………….. **10**
S5. Post-processing of linkers after generation …..……………………… **14**
S6. Reward function …………………………………………………… **15**
S7. Design modes ……………………………………………………… **22**
S8. Experimental Section ……………………………………………… **25**
S9. Flow model ………………………………………………………… **28**
S10. Molecular Simulations ……………………………………………... **37**
S11. References ………………………………………………………... **38**

Section S1. Edge-transitive nets and compatible nodes

MOF structures can be described as periodic graphs (or nets) with coordination between nodes (vertices) and spacers (edges).[1–3] In such a representation, framework assembly involves embedding this topological graph into Euclidean space under strict geometric and chemical constraints. Not all nets, therefore, correspond to achievable design targets due to topological symmetry and connectivity heterogeneity – which, in turn, complicates algorithmic assembly and synthesizability. We thus focus on nets that are edge-transitive, i.e., edges which are symmetry-equivalent; each edge has identical local coordination and geometric identity.[2] These nets offer several advantages for inverse design. First, they require only a single type of linker, eliminating the combinatorial complexity of multivariate systems. Second, the geometric constraints are uniform, i.e., connectivity, span, and orientation are consistent. Third, MOFs based on such nets are easier to experimentally synthesize. Although restricting the design space to edge-transitive nets reduces the dimensionality of the overall inverse-design problem, these nets still admit a vast and theoretically unbounded chemical space through linker design. By varying the linker chemistry within fixed reticular assembly constraints, it remains possible to explore an extensive range of chemically distinct architectures while substantially simplifying the inverse-design problem.

A second constraint we impose is the use of zero-periodic metal-cluster building blocks (MBBs). These MBBs (such as Zr_6-oxo, Cu_2-paddlewheel, Zn_4O) have well-defined topological coordination behavior, making algorithmic assembly tractable. Such MBBs have led to the development of several chemically stable MOFs with well-defined properties.[2] In contrast, supramolecular building blocks (SBBs), extended metal-chains or merged-net approaches introduce multiple additional degrees of freedom which drastically complicate algorithmic assembly and thus, inverse design. For detailed discussions on edge-transitive nets, MBBs and principles involving targeting such nets, we refer readers to some excellent reviews from Eddaoudi and co-workers.[1,2,4]

The successful assembly of nodes and designed edges on a target net would necessitate compatibility with geometric constraints. This would involve the connectivity of the linker being compatible with the target net, the span and orientation of the linker being within a reasonable range of the target values and the absence of steric clashes that would prevent coordination or lead to overlapping atoms. Certain nets, such as *pcu* and *fcu,* are much easier to target due to lower topological constraints, while nets with higher symmetry, such as *csq,* are more difficult. Consequently, these 'simpler' nets will have higher success rates for assembly compared to the 'difficult' nets. Most nodes we explore here have coordination numbers ranging from 6 – 12, which include clusters such as Zr_6-oxo (seen in UiO-66, PCN-222) and Cu_2-paddlewheels (seen in HKUST-1).

Our present work, therefore, does not address: **i**) multi-linker or multi-edge nets[5]; **ii**) mixed-node systems; **iii**) merged nets[6]; **iv**) periodic SBUs[7]; and **v**) interpenetrated structures.[8] We foresee that future work will expand to the joint optimization of node geometry and improved algorithmic assembly, enabling the targeting of such complex nets.

Section S2. Nexerra^{R1} architecture

S2.1. Sequence representation and SELFIES. Nexerra^{R1} is developed on molecular linkers represented as a sequence of tokens. The model is trained to predict the next token in a sequence given an initial set of tokens (**Figure S1 a**, **b**). Sequence representation is thus crucial to the development of a robust language model. We chose SELFIES (SELF-referencing embedded string)[9] as a string-based molecular representation for developing Nexerra^{R1}. The key advantage of SELFIES is that it is built on a formal grammar which guarantees chemical validity of generated sequences, which is a known problem of conventionally employed representations such as SMILES strings, especially on long sequences. For discussions on these formulated grammar rules and working principles of SELFIES, we refer readers to parent publications from Aspuru-Guzik and colleagues.[9,10] **Figure S1 b** shows the SELFIES string of a representative linker.

The SELFIES string (say x) is subsequently decomposed into a sequence of discrete tokens drawn from a finite (defined) vocabulary V, where each token corresponds to a structural unit. Let

$$x = (x_1, \dots, x_L), \qquad x_t \in V \tag{2.1}$$

denote the resulting tokenization of a SELFIES string. We append start-of-sequence '[SOS]', end-of-sequence '[EOS]' and padding '[PAD]' symbols to enable autoregressive training. Each sequence is padded to a fixed maximum length (102 tokens) with the [PAD] symbol masked during training and inference.

S2.2. β-VAE. Nexerra^{R1} is formulated as a β-VAE with transformer-based encoder and decoder networks. While detailed mathematical motivations on the VAE and transformer networks remain outside the remit of present work (instead read ref.[11,12]), we formulate the working principles of the architecture. Given the token sequence x from (2.1), we introduce a continuous latent variable z as follows:

$$z \in \mathcal{R}^{d_z};\ \ d_z = 128 \tag{2.2}$$

The probabilistic latent variable model defines:

$$p_\theta(x, z) = p(z) p_\theta(x|z) \tag{2.3}$$

with a Gaussian prior

$$p(z) = \mathcal{N}(0, I) \tag{2.4}$$

The transformer encoder maps these sequences to contextualized embeddings to produce a fixed-dimensional representation (**Figure S1 c**). From this, the approximate posterior is parameterized as follows:

$$q_\phi(z|x) = \mathcal{N}\left(z; \mu_\phi(x), diag\left(\sigma_\phi^2(x)\right)\right) \tag{2.5}$$

The latent samples can be drawn from the posterior using the reparameterization trick,

$$z = \mu_\phi(x) + \sigma_\phi(x) \odot \epsilon;\ \ \epsilon \sim \mathcal{N}(0, I) \tag{2.6}$$

The transformer decoder (**Figure S1 e**) – conditioned on z – defines an autoregressive likelihood,

$$p_\theta(x|z) = \prod_{t=1}^{L} p_\theta(x_t | x_{<t}, z) \tag{2.7}$$

We linearly project z from the latent space to the model space, and input it to the decoder via the cross-attention mechanism. The model parameters are optimized using a β-weighted evidence lower bound (ELBO),

$$\mathcal{L} = \mathrm{IE}_{q_\phi(z|x)}[log p_\theta(x|z)] - \beta D_{KL}(q_\phi(z \mid x)\ ||\ p(z)) \tag{2.8}$$

For reconstruction, we implemented a cross-entropy loss with padding-token masking. During training, to prevent posterior collapse, we gradually anneal the β-term starting from 0 during a warmup phase. Post-warmup, the β is held at a fixed value for the rest of the training. Due to the powerful learning capabilities of the transformer neural network, we find β = 0.01 provided stable training (for training curves, see **Section S4**).

S2.3. Transformer encoder and decoder. Both the encoder and decoder are implemented as stacks of transformer layers (**Figure S1**). We use a standard implementation as reported by Vaswani et al.[12] Given query (Q), key (K) and value (V) representations, scaled dot-product attention is defined as:

$$Attention(Q, K, V) = softmax\left(\frac{Q.K^T}{\sqrt{(d_k)}}\right)V \tag{2.9}$$

Each encoder layer applies multi-head self-attention followed by the conventional feedforward network and residual connections. In our context, the self-attention mechanism allows each token to attend to all others in the sequence – thereby capturing long-range structural dependencies within the linker. This is particularly important for MOF linkers, which often have extended conjugation and coordination motifs where geometric relationships cannot easily be captured. The decoder too, retains the attention-based working, but incorporates masked self-attention to preserve its autoregressive nature. In other words, we mask future tokens so that the decoder cannot 'cheat'. We condition on the latent variable using cross-attention.

We chose transformer networks for our architecture due to their ability to model long-range interactions and accommodate longer context windows without degradation. This is evidenced by high per-token reconstruction accuracies for a 102-token window. That being said, such architectures are not without their share of problems. The powerful learning capabilities require strong control over model hyperparameters to prevent memorization, while the high expressivity necessitates strong geometrical and chemical filters coupled with reward functions to generate desirable linkers.

S2.4. Inference. At inference, during unconstrained sampling, new linkers are generated by sampling from the latent prior, $z \sim \mathcal{N}(0, I)$ followed by the autoregressive decoding of tokens described in Equation 2.7. To control the extent of diversity (i.e., how similar or different the samples are), we scale the generated logits by a temperature parameter $T(> 0)$. Lower values make the distribution sharper – i.e., less diversity – while higher values increase diversity. We get best results at $T = 0.8$. However, sampling is flexible. To prevent sampling logits with extremely low probability (which leads to sub-optimal generated linkers), we perform nucleus (or top-p) sampling.[13] This means that at $p = 0.9$, at each step, we only perform sampling on the smallest subset of tokens whose cumulative probability mass reaches or exceeds 0.9. This eliminates sub-optimal tokens from consideration – improving inference stability. Inference continues until we hit an '[EOS]' token or reach the maximum token limit of 102. Since SELFIES enforces validity, all generated molecules are syntactically and semantically valid.

Following sampling, we implement a post-processing algorithm that optimizes the placement of coordination anchors – i.e., the [Lr] token. This algorithm is described in **Section S5**. Moreover, what we have described here is unconstrained sampling. A more efficient methodology involves seed-guided design (as introduced in the main manuscript). These design methods have been described in **Section S7**.

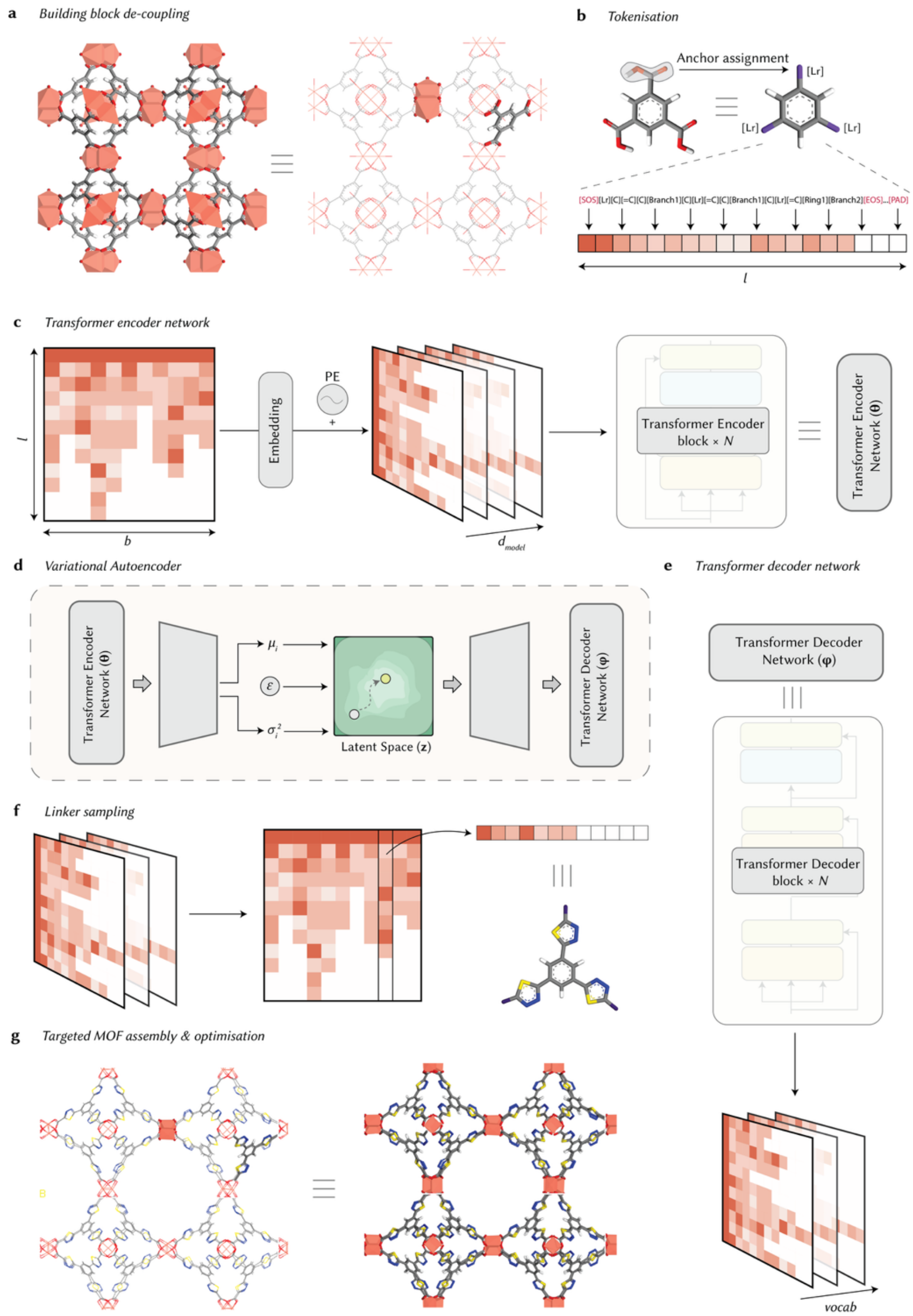

Figure S1. Nexerra^{R1} architecture. a. MOF structures are represented as nodes and edges. As a first step, we decouple the design process at the building-block level. **b.** Linkers are encoded as molecular strings and tokenized into a sequence of symbols. **c.** The encoder network of the model is composed of a stack of transformer layers that map tokenized vectors in one representation space into a corresponding set of vectors in a new space, and by doing so, learn a rich internal representation. The token sequences are first embedded and subsequently undergo position encoding (PE) to capture sequence order. This embedded tensor is then passed to the transformer stack which applies the self-attention mechanism to capture long-range interactions. **d.** The VAE acts as a bottleneck wherein the encoder defines an approximate posterior over a low-dimensional continuous latent variable (z), while the decoder reconstructs the original sequence from z. **e.** The decoder is again composed of a stack of transformer layers that autoregressively reconstructs the token sequence conditioned on the latent representation and outputs logits over the vocabulary (*vocab*). Here we implement masked self-attention to prevent the decoder from seeing future tokens during training. We condition it on the latent variable using cross-attention. **f.** Nucleus sampling is used to generate new linkers from the decoder output. **g.** Subject to chemical validity, an attempt is made to assemble the new linker into the target net (Color code: Polyhedron - Cu_2(μ-COO_4; Gray - C; Red - O; Blue - N; Yellow - S; White - H; Purple - [Lr]).

Section S3. Dataset curation and generation

While there is a lack of consensus on how much data is required to train CLMs,[14,15] when it comes to specific domains such as MOF linkers, datasets spanning 10^5 – 10^6 samples generally yield good results.[16] Starting from the dataset reported by Aspuru-Guzik et al.[16], we extracted ~ 80,000 unique molecules (as SMILES). In order to expand the dataset, we implemented a randomized monovalent functionalization protocol to augment SMILES data. **Algorithm C1** details the developed functionalization protocol. Briefly, for each original molecule: we identified heavy-atom sites bearing hydrogens, randomly selected a replaceable site, removed one hydrogen and appended a fragment from a curated list (**Figure S2**) by combining the parent and fragment molecules, forming a single bond at the attachment site. Each generated molecule is then sanitized using an open-source cheminformatics library (Rdkit[17]) and discarded if this sanitization or valence checks fail. To favor synthetically accessible modifications – candidates with SCScore > 4.75 were discarded. Each molecule from the original dataset undergoes up to 20 such attempts (to compensate for rejected modifications) and up to 3 monovalent functionalization steps per attempt (number of attempts selected at random). The resulting molecules were canonicalized and filtered for duplicates. This ultimately yielded a final training corpus of 898,677 unique molecules.

Algorithm C1: Dataset generation

Input: Parent molecule set $\mathcal{D}_0 = \{d_1, \ldots, d_N\}$;
Fragment set $\mathcal{F} = \{f_1, \ldots, f_K\}$;
Maximum attempts $A = 20$;
Maximum substitutions per attempt $S_{\max} = 3$;
Synthetic complexity (SCScore) threshold $\tau = 4.75$
Output: Augmented molecule set $\mathcal{D}_{aug}$
Initialize $\mathcal{D}_{aug} \leftarrow \emptyset$
foreach $m \in \mathcal{M}_0$ **do**
  Identify eligible attachment sites:

$$\mathcal{H}(m) = \{a \in \text{HeavyAtoms}(m) \mid \text{HydrogenCount}(a) \geq 1\}$$

  for $i = 1$ *to* A **do**
    $m' \leftarrow m$ Sample number of substitutions:

$$s \sim \text{Uniform}\{1, \ldots, S_{\max}\}$$

    for $j = 1$ *to* s **do**
      Randomly select attachment site:

$$a \sim \text{Uniform}(\mathcal{H}(m'))$$

      Randomly select fragment:

$$f \sim \text{Uniform}(\mathcal{F})$$

      Remove one hydrogen from a
      Form single bond between a and attachment atom of f
      Sanitize m' (valence check, RDKit sanitization)
      if *sanitization fails* **then**
        break
    if *sanitization succeeds and* $SCScore(m') \leq \tau$ **then**
      Canonicalize m'; Add m' to $\mathcal{M}_{aug}$
Remove duplicates from $\mathcal{M}_{aug}$
return $\mathcal{M}_{aug}$

Given the scale of the dataset, although we do not have precise control over the quality of each generated molecule, the rigid SCScore cutoff and the sanitization and canonicalization protocols are expected to ensure that poor quality molecules are eliminated.

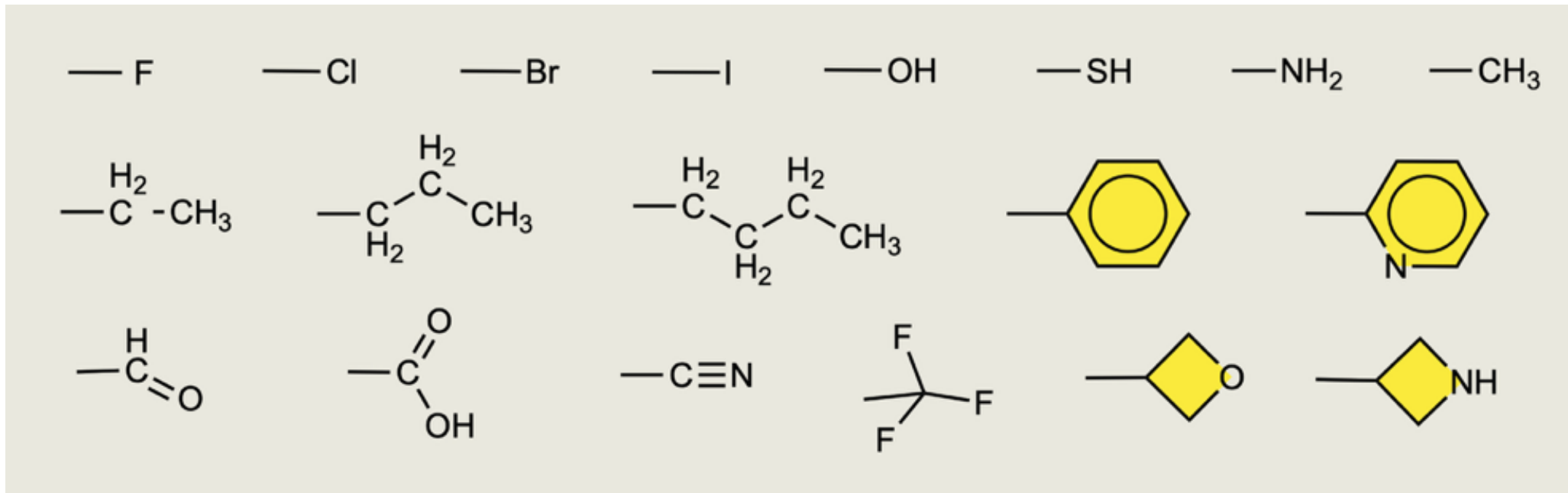

Figure S2. List of fragments used to randomly functionalize linkers to expand the training corpus.

Figure S3 shows distributions of molecular properties for the extended dataset post-functionalization (n = 200,000). The heavy atom count distribution is roughly unimodal, with most linkers in the medium-large regime (i.e., ~ 20 – 30 heavy atoms) and relatively few short linkers (< 10 heavy atoms). Accordingly, the model is expected to learn medium-large linkers best, with limited support for very short linkers. The logP follows a bell-shaped curve – spanning both polar and hydrophobic linkers, and centered in a moderately lipophilic regime. Seeing no massive spikes at extreme ends is encouraging as it indicates that the data is not biased towards extremely polar or lipophilic molecules and covers a healthy range. The SCScore distribution is also reasonable – with the dataset explicitly biased away from extremely difficult linkers (i.e. scores > 4.75). There is a broad peak around 2 – 4, with very few molecules in the hyper-trivial regions (scores near 1). There is thus, a meaningful spread of synthetic difficulty – capturing what one would expect for MOF linkers. For rotatable bond counts (as a proxy for flexibility), the distribution is concentrated towards ~ 5 - 10 rotatable bonds, with a tail > 20 and some entries close to 0. There is a mix of rigid, semi-rigid and flexible linkers (here, it is expected that functionalization would have increased these counts) – but the distribution is not overly skewed. The molecular weight distribution is also unimodal – capturing small bidentate to complex multidentate linkers. Overall, while functionalization gently skews some properties, the resulting distributions are smooth, unimodal and span chemically sensible ranges, yielding broad coverage biased towards medium-large, moderately flexible, moderately lipophilic, yet synthetically tractable linkers.

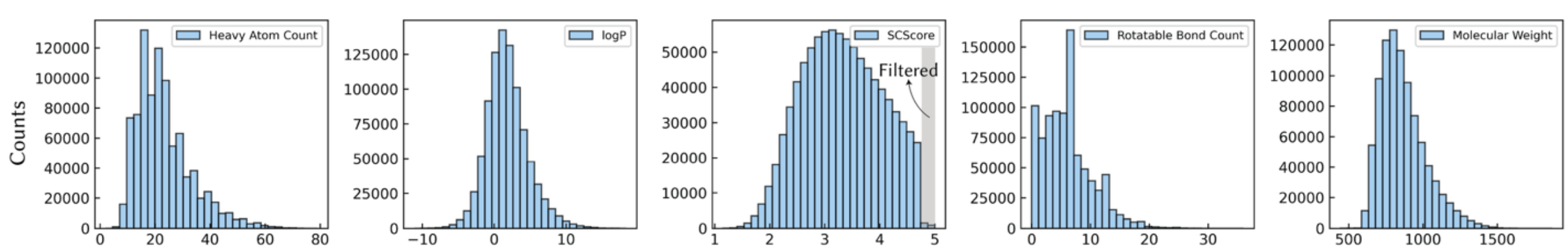

Figure S3. Distributions of molecular properties – (from left to right) heavy atom count, logP, SCScore, rotatable bond count and molecular weight – for the extended dataset (n = 200,000 sampled randomly from a total 898,677 molecules). **Note:** The steep cliff observed in the SCScore distribution at large values is because we have filtered values exceeding 4.75 during the functionalization process.

Figure S4 shows UMAP embeddings of linkers from the training dataset (Morgan fingerprints, radius 2, 2048 bits, n = 50000) colored by logP, molecular weight and rotatable bond count – providing a complementary view of the corpus. The linkers occupy a single, coherent manifold with only a few small satellite regions, suggesting that the training data forms a connected chemical space rather than several isolated clusters. This suggests that the training distribution is continuous rather than being disjoint into isolated clusters (although some satellites do exist on the UMAP

plot). Such distributions are favourable for models that rely on interpolation in the latent space. Variations in logP, size and flexibility are distributed across this manifold. There is some degree of qualitative visual coincidence between higher molecular weights and increased rotatable-bond counts, as expected for functionalised multidentate linkers. Regions with extreme values of these properties are present but relatively sparse and remain embedded within the main manifold, in agreement with the one-dimensional descriptor distributions (**Figure S3**).

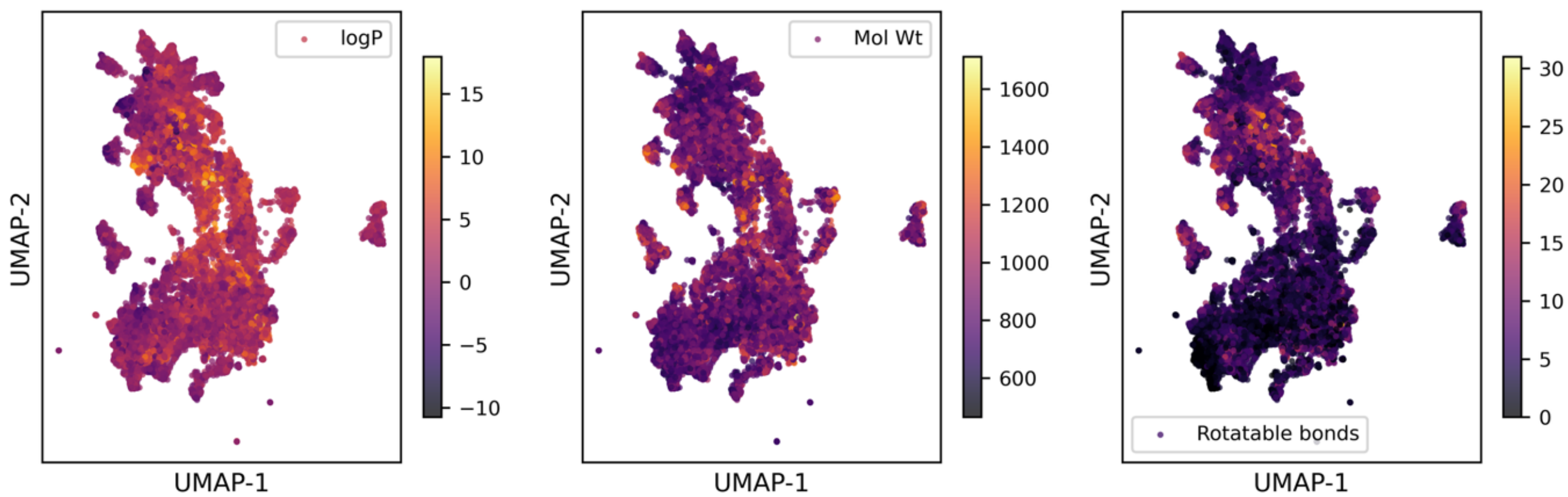

Figure S4. UMAP visualization of the training corpus (n = 50,000, Morgan fingerprints, radius 2, 2048 bits). Colored by (left-right) logP, molecular weight and rotatable bond counts. Note: The colors have been normalized for each property class.

Across the ~ 900,000 linker corpus, we identified ~ 103,000 unique Bemis-Murcko scaffolds – representing an ~ 11% uniqueness. Among these, the top 50 scaffolds (**Figure S5**) account for 30% of all linkers, implying that ~ 0.05% of scaffolds – representing a small number of chemically simple cores – are reused, while the remaining cores have a longer tail of rarer, more complex motifs, not captured among the common cores. The commonly observed scaffolds are dominated by benzenoid and N-heterocyclic cores together with a few biaryl and fused aromatic systems, typical in MOF chemistry. The scaffold distribution provides frequent analogues on well-known MOF cores but also implies a bias towards these cores, with less statistical support for under-represented (complex) scaffold families. However, we anticipate that the scaffold-constrained design approach (discussed in the main manuscript) should be able to mitigate some of these biases.

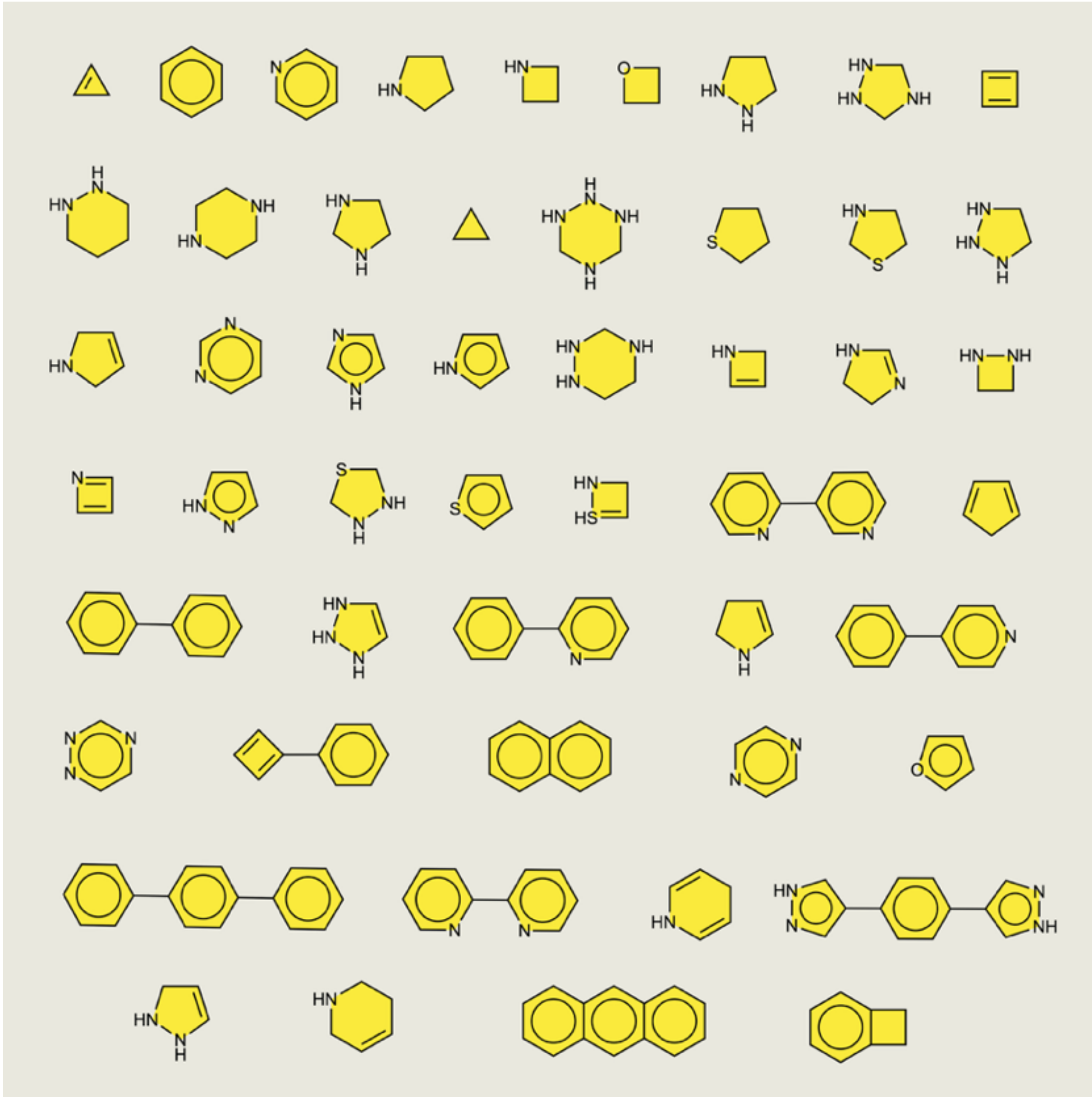

Figure S5. The 50 most commonly observed Murcko scaffolds from the training dataset accounting for ~30% of the entire corpus. Ranked in decreasing order of prevalence from left-right, top-bottom.

Section S4. Model training & evaluation

Nexerra[R1] was trained over 172 epochs, with early stopping selecting a checkpoint around 120 epochs. **Figure S6** shows the training logs, which exhibit behavior consistent with stable training. The total and reconstruction loss rapidly decrease over the first few epochs. The slight increase in total loss in the 50 – 100 epoch range coincides with an increase in the β-weight assigned to the KL term in the loss function. Around a similar epoch-range, the KL loss exhibits a steep jump (corresponding to KL-free learning schedule) before gradually decreasing and stabilizing. The KL loss stabilizing at ~ 25 is encouraging as it indicates that the model avoids posterior collapse, thanks to the β-warmup scheduler. This indicates that the latent space has meaningfully regularized and has not degenerated into a purely deterministic autoencoder. Parallelly, the per-token reconstruction accuracy jumps from ~ 20% to > 90% over the initial stage of training, following which it gradually stabilizes to ~ 96.5% over the training duration. For samples drawn during the training process, the internal diversity also stabilizes to slightly > 0.925, which is close to what we observed for the training dataset, another encouraging sign. As for the novelty of these sampled molecules, it lies in the healthy 96 – 99.5% range. The small fraction of exact reconstructions (causing the slight dip in novelty) is consistent with the model's ability to reproduce training-like linkers while still generating predominantly novel molecules. Overall, the training logs support a non-degenerate representation of the MOF linker space that is capable of generating novel linkers.

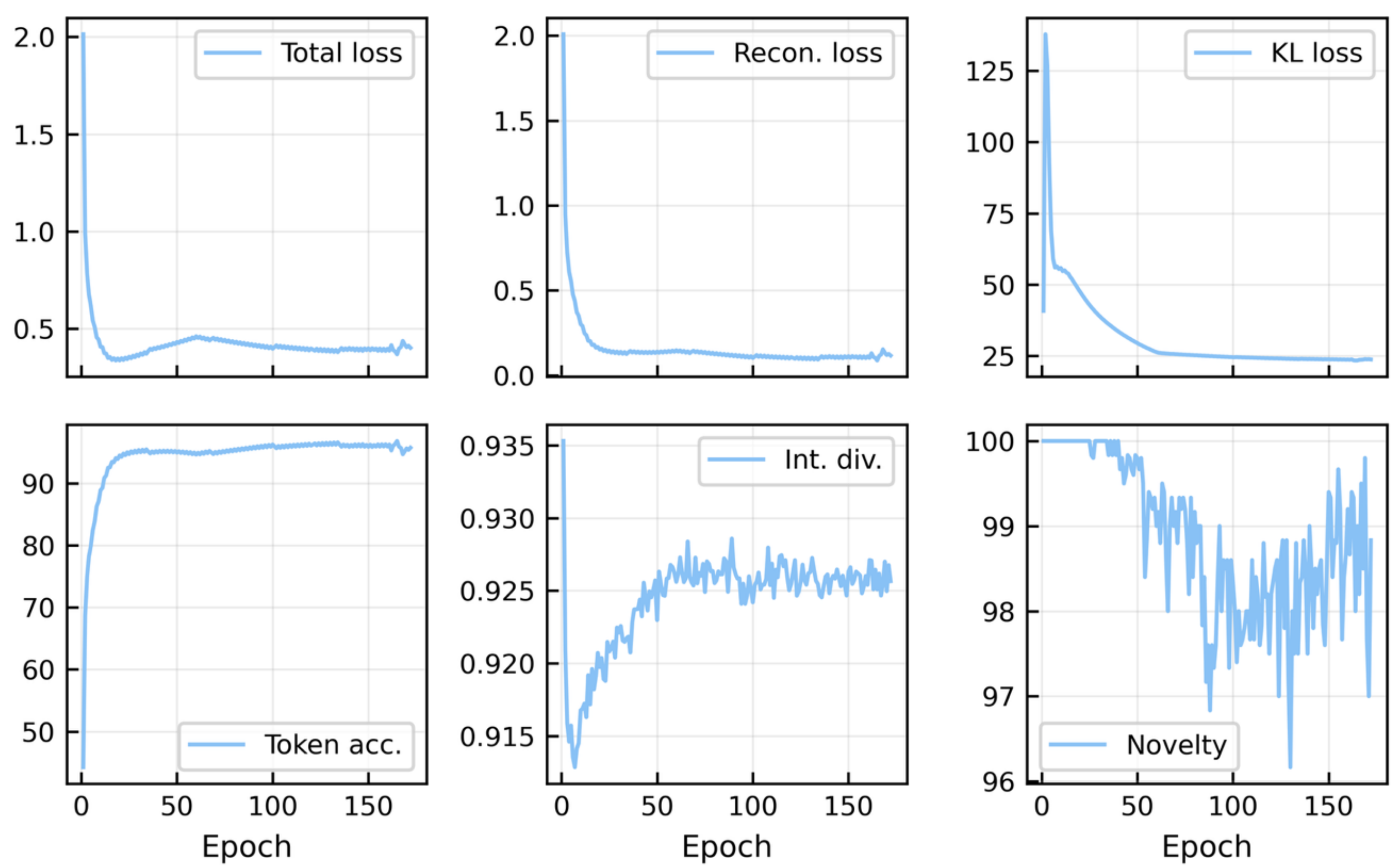

Figure S6. The training logs measured over 172 epochs indicate stable training. (First row, left to right): Total loss, reconstruction loss and KL loss. (Second row: left to right): Per-token reconstruction accuracy, internal diversity of sampled molecules and novelty of sampled molecules (n = 5000).

Beyond training metrics, a key test for a generative model is whether it reproduces the physicochemical characteristics of experimentally reported molecules. For MOF linker design, properties such as hydrophobicity, polarity, molecular size, saturation, and ring architecture influence synthetic accessibility, framework assembly,

rigidity, and ultimately, performance. To assess whether the model captures these chemically meaningful characteristics, **Figure S7** compares distributions of six physicochemical descriptors between 10,000 generated molecules and 10,000 molecules drawn from a previously unseen test set (logP, topological polar surface area (TPSA), fraction Csp3, molecular weight, ring count, and aromatic ring count).

For logP, both the generated and test distributions are unimodal and centred in a similar region, although the generated molecules show a modest right shift, indicating a slight bias toward more lipophilic species (KL = 0.143). Fraction Csp^3, which signals the balance between saturated and aromatic character and therefore influences linker flexibility and rigidity, spans the full 0-1 range in both sets; the test distribution is more concentrated near the extremes, whereas the generated distribution is flatter (KL = 0.074). TPSA, a measure of molecular polarity and hydrogen-bonding capacity, is reproduced particularly well, with only a very small divergence (KL = 0.013). Molecular weight shows a slight right shift in the generated set, consistent with somewhat larger molecules overall, although the two distributions remain broadly similar (KL = 0.109). Ring count, which reflects structural complexity and rigidity, is also well reproduced (KL = 0.065), while aromatic ring count, a particularly relevant descriptor for MOF linkers given the prevalence of aromatic building blocks in reticular chemistry, shows especially strong agreement (KL = 0.017).

Taken together, these results indicate that the VAE reproduces several key physicochemical characteristics of experimentally reported MOF linkers. The closest agreement is observed for TPSA and aromatic ring count, with similarly good agreement for fraction Csp^3 and total ring count, while modest shifts are observed for logP and molecular weight. Overall, the model generates molecules that occupy the same broad chemical design space as known MOF linkers, supporting the chemical realism of the learned latent representation.

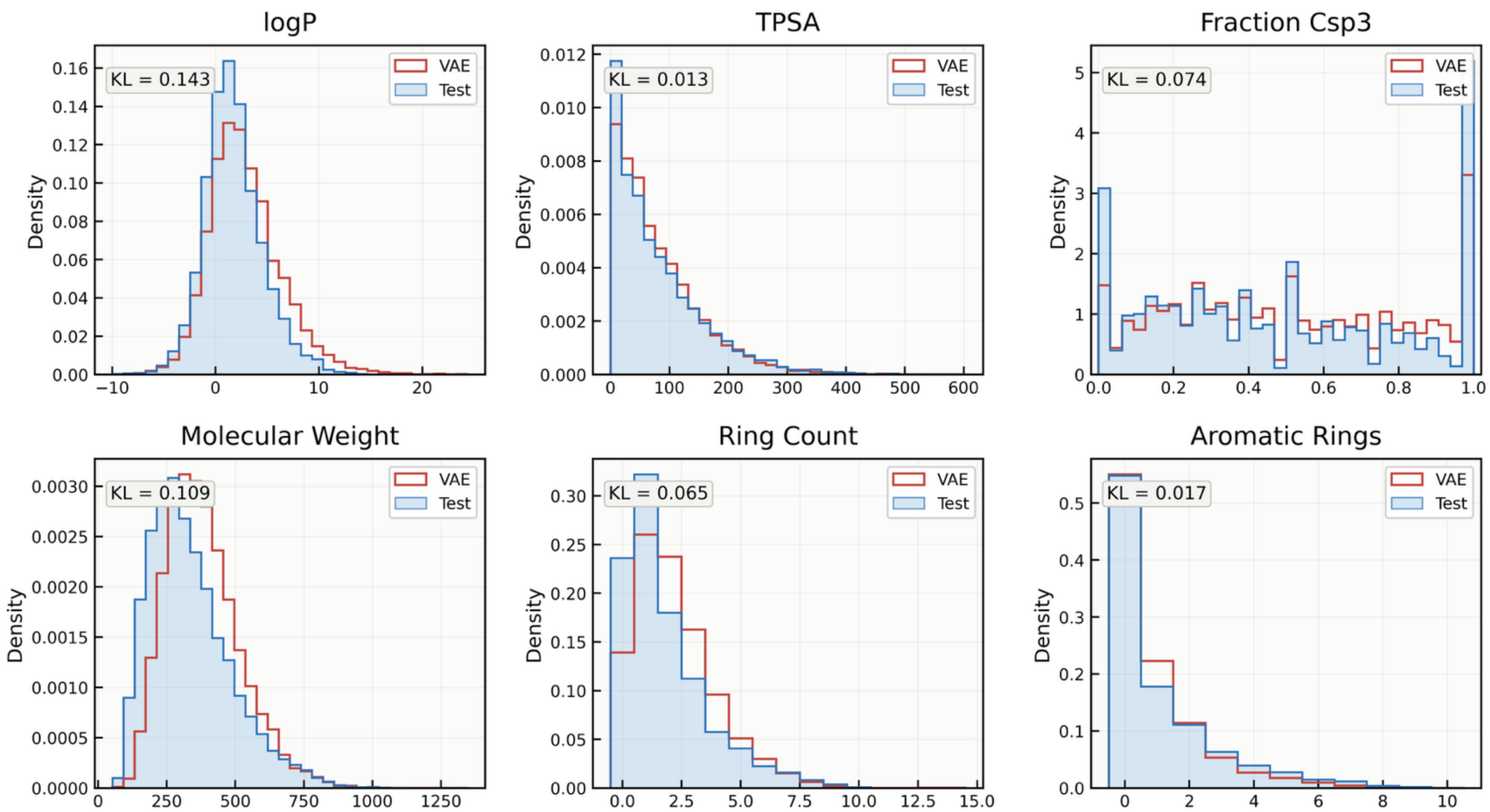

Figure S7. Distributions for logP, fraction Csp3, TPSA, molecular weight, ring count, and aromatic rings for generated molecules (n = 9,998) and a non-redundant subset of the test set (n = 10,000). The annotated text represents the KL divergence between the two distributions. A KL divergence of 0.0 would indicate a perfect match (i.e., the ideal case).

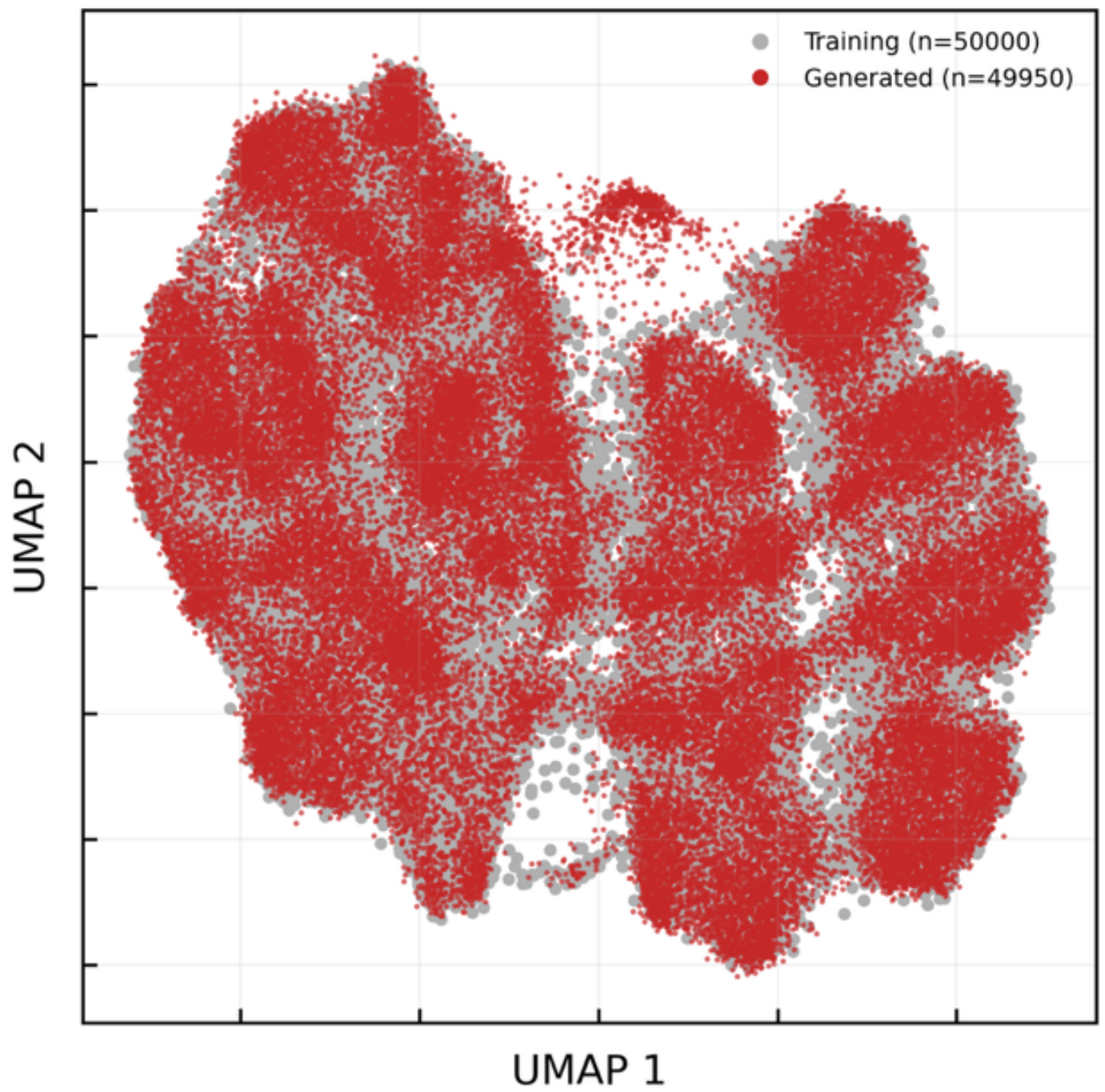

Figure S8. UMAP visualization of the latent embeddings of training molecules (gray, n = 50,000) and generated molecules (red, n = 49,950). The generated samples closely overlap the training manifold, although, there is mild clustering of generated molecules in some pockets. Additionally, there are no major pockets outside the training manifold, suggesting that sampling remains within the learned latent space.

Next, to assess the continuity of the learned latent representation, we projected 50,000 training embeddings and 49,950 samples drawn from the latent prior into a shared UMAP embedding (**Figure S8**). The generated samples closely overlap the empirical manifold without forming explicitly disjoint clusters or major regions outside the training manifold. This suggests that samples drawn from the prior occupy regions broadly overlapping with the encoded training distribution in the low-dimensional UMAP projection.

Figure S9 shows examples that illustrate the structures Nexerra[R1] can access under free sampling from the prior. The model readily proposes multi-functional, high-connectivity linkers with extended aromatic cores and dense substitution patterns. This complexity reflects both the expressivity of the language model and its large context window (up to 102 tokens), which allows it to coordinate long-range patterns such as symmetry, repeating units and balanced placement of coordination groups along the backbone. However, we must stress that these are representative high-quality samples selected from a much larger pool: in unconstrained free sampling, the same expressive capacity also produces many molecules that are synthetically unappealing, poorly suited to MOF linkers, or simply off-target for the design objective. In other words, the model's raw generative power does not automatically translate into an efficient 'hit rate' for good candidates. In practice, we therefore rely on seed-guided design, where a chemically reasonable linker template or scaffold is provided and the model is used to explore the chemical vicinity of this scaffold in the latent space pursuant to a reward function, to focus generation onto promising regions of linker space and substantially improve the efficiency of discovering useful linkers.

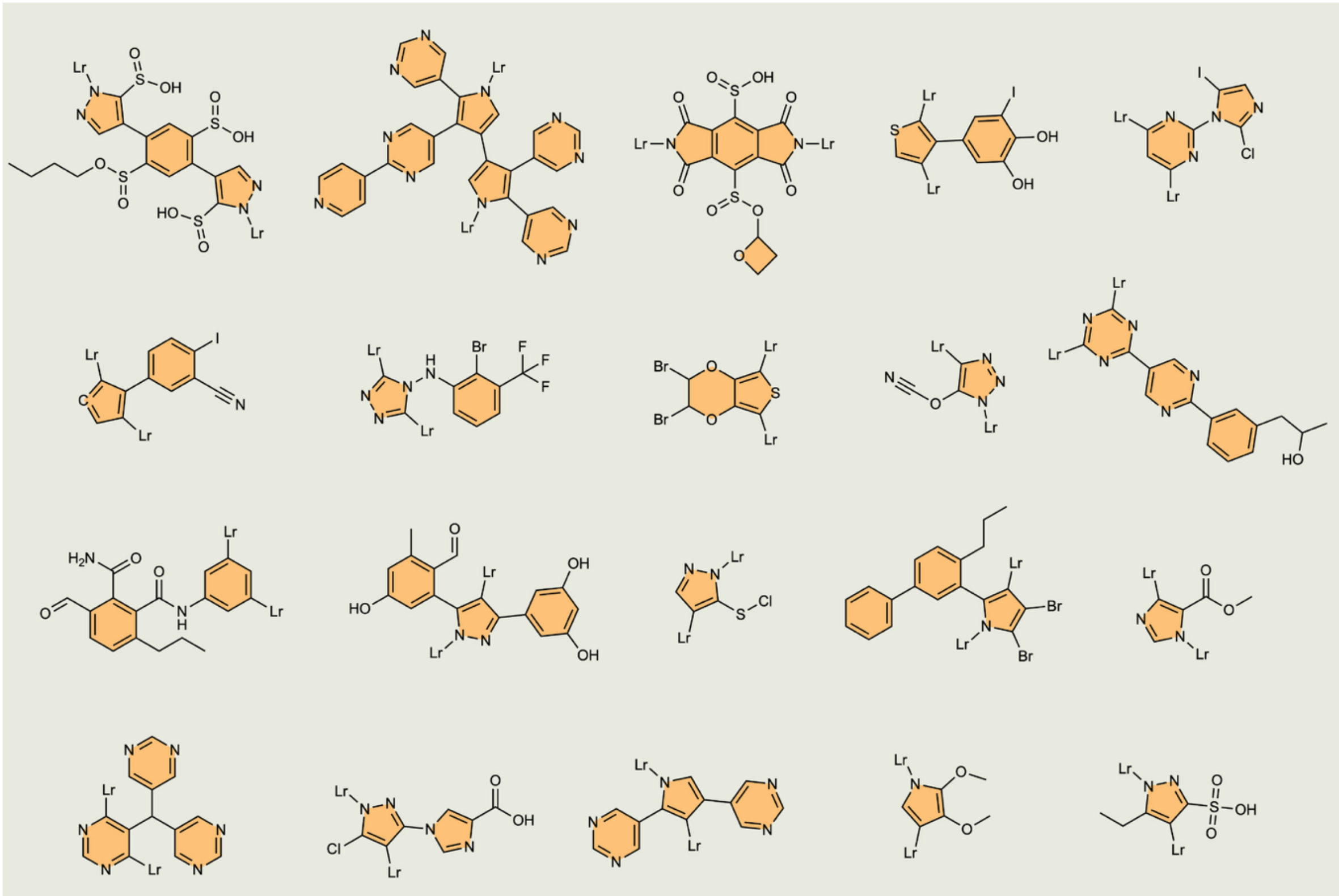

Figure S9. Illustrative examples of molecules sampled freely from the prior of Nexerra[R1]. The model is able to generate multi-functional, complex molecules with polyaromatic cores and dense substitution patterns. However, owing to the high expressivity of the CLM, under unconstrained sampling, the 'hit rate' for desirable linkers is low. Thus, seed-guided design serves as a more optimal route towards the discovery of exceptional linkers.

Finally, using the 10,000 linkers sampled from the latent prior, we computed standard distributional diagnostics. All generated molecules were chemically valid (validity = 1.00; as expected when using SELFIES), with negligible duplication (uniqueness = 0.99), indicating that the decoding is stable, with no mode collapse. Internal diversity remained high (IntDiv = 0.925), comparable to that of the training distribution, suggesting broad exploration of linker chemotypes. The average similarity to the nearest molecule (SNN) = 0.26, reflecting moderate structural proximity without memorization; this is also confirmed by the latent space embeddings (**Figure S8**), which show that the sampled molecules occupy the same chemical space as the training dataset. The scaffold diversity (Scaff = 0.92) confirms that linker generation is not confined to a narrow subset. Put together, these metrics indicate that sampling from the VAE prior produces a coherent and chemically diverse distribution, providing a stable foundation for subsequent distributional targeting via flow.

Section S5. Post-processing of linkers after generation.

Once generated, molecules are subjected to standard sanitization checks using RDKit[17]; these include valency checks, aromaticity and ring assignment checks. Should a generated molecule fail this, it is immediately discarded from any further consideration. This is, however, rare, considering we use SELFIES representation for the model which guarantees validity.

To coordinate with the metal-containing cluster (i.e., the node), the linker should have coordinating moieties. In practice, these are usually groups such as carboxylates, phosphonates, sulfonates or azolates. However, due to the nature of the MOF assembly algorithm[18] that we use, we instead denote these moieties using an '[Lr]' token. Now, in addition to having these '[Lr]' tokens, they must be placed onto the generated linker in a manner that ensures geometric and topological compatibility with the target net.

First, we remove all existing '[Lr]' tokens; in case this token is on a ring system, it is substituted with a Nitrogen atom. This resulting scaffold is then geometry optimized. The number of coordination handles to be placed either depends on the input seed (i.e., to match the coordination of the seed) or alternatively, can be specified by the user. In principle, the algorithm is agnostic to the number of such handles. Next, we embed the linker in Euclidean space and define candidate attachment sites as the set of carbon atoms with at least one removable hydrogen atom. Let $S = \{s_1, \dots, s_n\}$ denote a set of 3D coordinates of these carbon atoms. In order to place k anchors, we enumerate all possible k candidate sites. For each candidate set $S_i = \{s_{i_1}, \dots, s_{i_k}\}$, we calculate the mean pairwise separation as,

$$\Phi = \frac{2}{k(k-1)} \sigma_{a<b} \left\| r_{i_a} - r_{i_b} \right\| \tag{5.1}$$

We select the configuration that maximizes Φ. This favors spatially well-separated anchors with good linker geometries and low steric crowding. This works especially well for ditopic and tritopic linkers. Following the selection of these candidate sites, we remove one hydrogen from these specified Carbons and replace it with an '[Lr]' token. **Figure S10** describes the linker optimization workflow adopted.

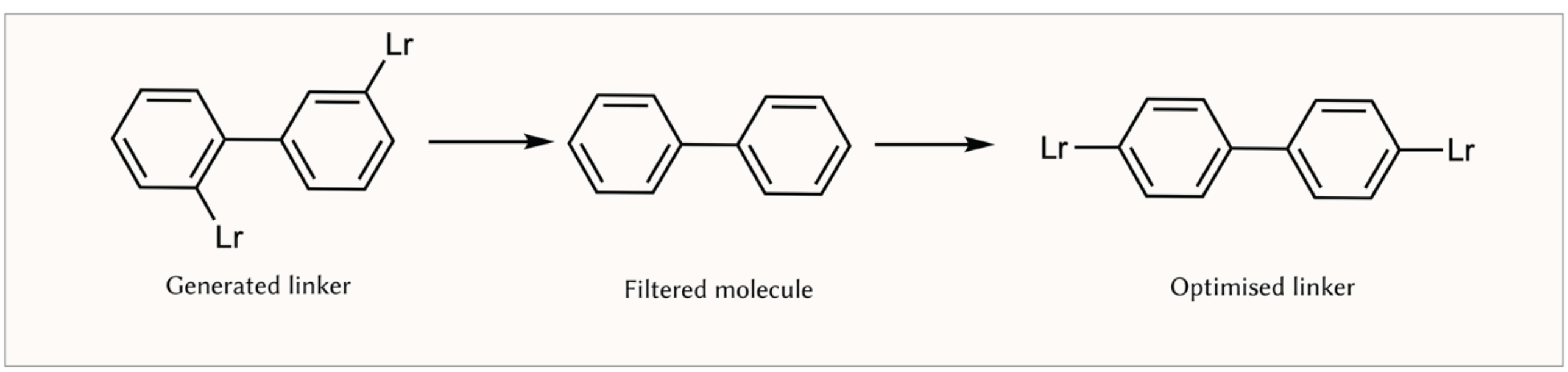

Figure S10. The linker optimisation workflow. Starting with a generated linker, it is stripped of its '[Lr]' handles, and optimised. Next, we collect a set of candidate attachment sites (described above) and select those sites which maximise the spatial separation between anchor points. We append the required '[Lr]' handles to these selected sites. This workflow is in principle, agnostic to the number of handles.

Section S6. The reward function

The generative capabilities of Nexerra[R1] do not extend to the efficient discrimination of useful linkers – i.e., linkers with favorable properties – against linkers that are sub-optimal. Even with the addition of the flow model (**Section S9**), we are at best able to favor linker sampling against certain target properties (for instance, linker length). For most applications, we need to optimize several, often competing properties. We thus rely on reward functions that score linkers based on their utility for the application of interest. In this context, reward functions offer several advantages: (**i**) they are modular, i.e., they can be easily formulated for an application of interest to screen for desirable linkers; (**ii**) they are computationally cheap and do not require any additional model training; and (**iii**) they can theoretically span arbitrary complexity. On the downside, these functions rely on several simplifications and assumptions, and on the whole are tough to generalize. Functions formulated for a system of interest, may not necessarily work well for other systems. Nonetheless, the flexibility on offer enables access to vast design spaces that in principle, span multiple application landscapes.

Here, as case studies, we formulate two reward functions targeting gas storage and biocompatibility. Before proceeding to the formulation of these functions, we preface the discussion by clarifying that these functions operate completely at the linker-level and do not explicitly account for metal-node and topology effects, host-guest interactions, and framework flexibility. These characteristics undoubtedly play critical roles in the overall performance, and can be evaluated using geometric analysis and molecular simulations on assembled frameworks. The goal here is to develop computationally lightweight structural filters to bias generation towards desirable chemotypes.

S6.1. Gas storage. In the design of adsorbents for gas storage (e.g., H_2, CH_4), the deliverable capacity is a critical design parameter.[19] Here, the gravimetric uptake – mass of adsorbate taken up per unit mass of adsorbent – and the volumetric uptake – mass of adsorbate taken up per unit volume of adsorbent – are two equally important, yet competing metrics.[19,20] Design strategies need to focus on concurrently optimizing both objectives. At the linker level, several structural characteristics influence framework-level gas storage capabilities: (**i**) anchor-to-anchor span, which modulates the node-node separation and the pore size; (**ii**) molecular weight, which controls framework density; (**iii**) rigidity, which impacts disorder and framework collapse; and (**iv**) linker symmetry, which impacts the packing efficiency.

Starting with gravimetric storage, the capacity benefits by maximizing accessible void volume per unit mass. To approximate this at the linker-level, we define a mass-normalized span descriptor as:

$$d_{mass-norm-span} = \frac{d_{anchor-anchor}}{MW} \tag{6.1}$$

Where, $d_{anchor-anchor}$ is the maximum anchor – anchor distance and MW is the molecular weight of the linker (excluding anchors). $d_{mass-norm-span}$ serves as a proxy for structural expansion per unit mass. This proxy – which is intended as a purely relative descriptor – biases generation towards longer, lightweight linkers. At the same time, such linkers tend to exhibit a high degree of flexibility, which makes ultraporous frameworks susceptible to potential pore collapse. We therefore introduce a second descriptor $d_{rigidity}$ that penalizes flexible linkers that may promote framework collapse. The rigidity proxy is defined as follows:

$$d_{rigidity} = 0.5 \times RBC + 0.5 \times N_{C_{sp^3}} \tag{6.2}$$

Where, RBC is the fraction of the rotatable bonds in the linker and $N_{C_{sp^3}}$ is the fraction of sp^3 Carbons in the linker. Put together, the gravimetric reward (R_{grav}) is defined as follows:

$$R_{grav} = \alpha_1 D(d_{anchor-anchor}) + \alpha_2 D(d_{rigidity}) + \alpha_3 D(d_{mass-norm-span}) \tag{6.3}$$

Where, D denotes bounded desirability functions that prevent extreme values from distorting the reward scores while enabling smoother interpolations; $\alpha_1, \alpha_2, \alpha_3$ denote the relative weights assigned to each term. In the case of the mass-normalized span descriptor, D is an increasing sigmoidal function centered around the 75th percentile of the distribution from the sampled training set (**Figure S11 a**). Due to the strong regularization effect of this descriptor that penalizes heavy linkers, it is assigned a lower weightage of 0.2. For the flexibility descriptor, D is a decreasing sigmoidal function centered around 0.5, with a width of 0.2. For the span descriptor, D is an increasing sigmoidal function centered around 16 Å, with a width of 4 Å. Overall, R_{grav} encodes structural priors that support lightweight, long-span, shape-persistent linkers. **Figure S11 b** shows the R_{grav} distribution of linkers from the training set (n = 25,000). The distribution has a median reward of 0.52 (p75 = 0.58, p25 = 0.43). Crucially, the distribution is not skewed to either extreme confirming that the rewards are not saturated; instead, the rewards span a healthy range of (0.2, 0.8). The absence of sharp cutoffs or collapsed modes indicates that the reward landscape is well-conditioned and preserves meaningful variance across chemically diverse linkers. The function should ensure that structural modifications produce graded changes in the reward – consistent with flow-based optimization.

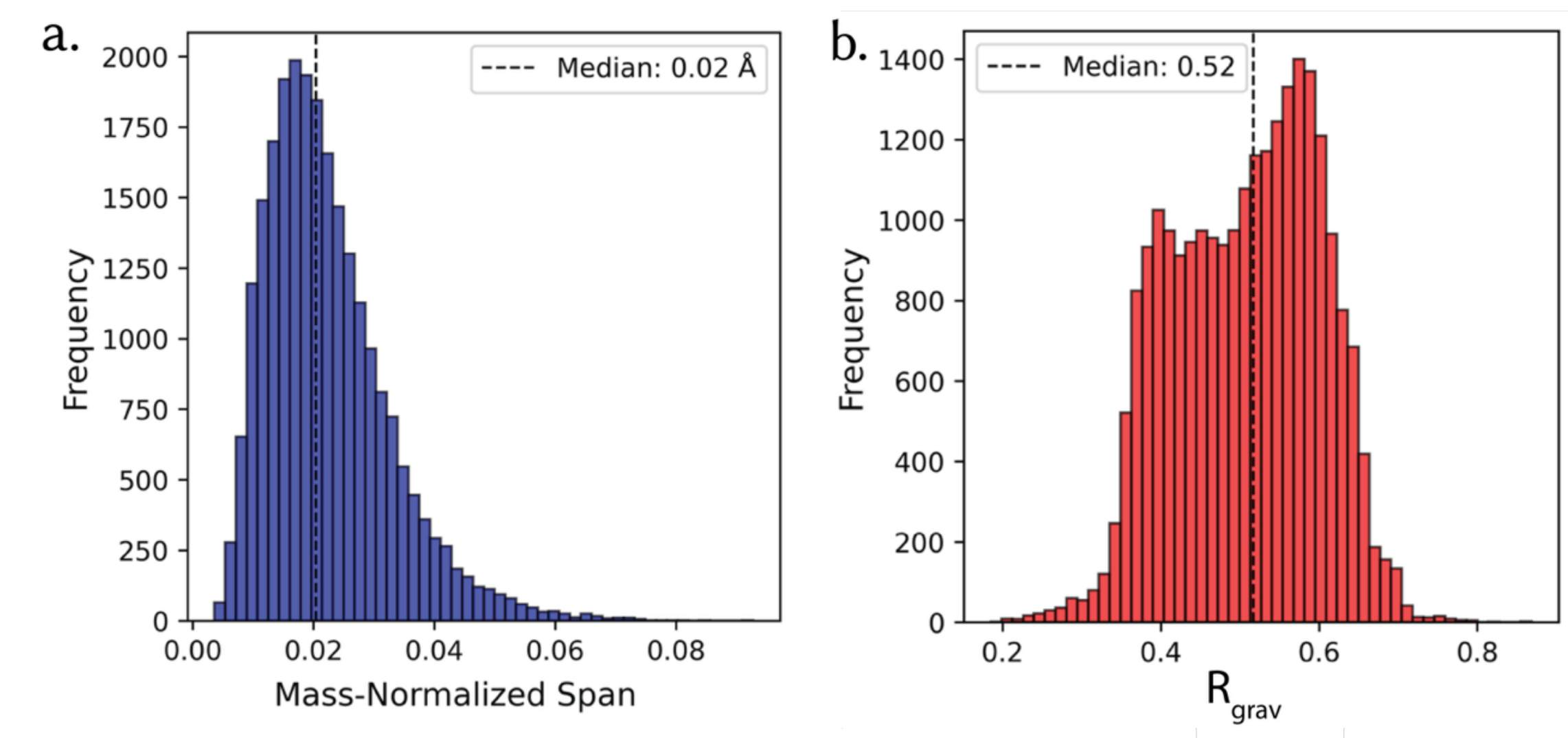

Figure S11. a. Distribution of the mass-normalized span for molecules from the training set (n = 25,000). Median = 0.02 Å mol g^{-1}; p75 = 0.03 Å mol g^{-1} and p25 = 0.01 Å mol g^{-1}. The increasing sigmoidal desirability function is centred around p75, with a width of 0.5 × (p75 – p25) to favor linkers with favorable mass-normalized spans. **b.** Distribution of the gravimetric reward score (R_{grav}) for molecules from the training set (n = 25,000). Median = 0.52; p75 = 0.58 and p25 = 0.43. Notably, the distribution is not skewed towards either extreme bound – which would have been indicative of collapse. The distribution spans almost the entire range and we also observe a smooth gradient towards higher scores, suggesting that a non-trivial fraction of the training linkers achieve high scores [$\alpha_1 = \alpha_2 = 0.4; \alpha_3 = 0.2$].

Figure S12 shows the R_{grav} scores computed for linkers corresponding to some of the top-performing MOFs for gravimetric CH_4 storage. The scores assigned to each linker roughly correspond to the observed CH_4 storage trends. Notably, the linker of CU-6 – a MOF recently reported by some of us[20] – which has the highest gravimetric CH_4 storage capacity to date, shows the highest relative R_{grav} score. We, however, stress that there are several aspects relating to metal-node effects, topology and volumetric packing that are not captured here; this remains a computationally efficient proxy for discriminating linkers.

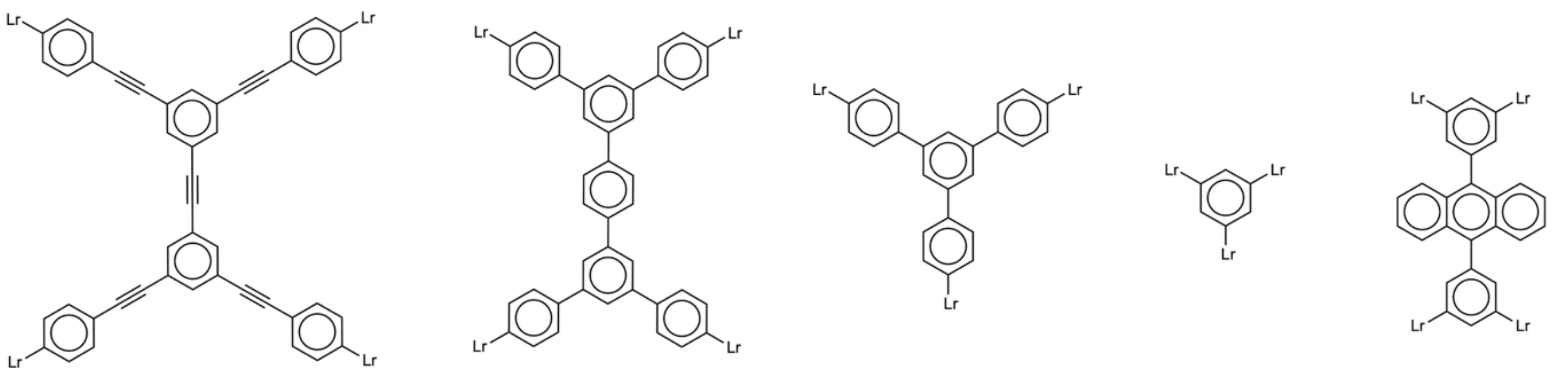

CU-6 (0.81) **Al-soc-MOF-1** (0.78) **MOF-519/-520** (0.68) **HKUST-1** (0.61) **PCN-14** (0.68)

Figure S12. R_{grav} scores for linkers belonging to top-performing MOFs for gravimetric CH_4 storage. The ranking of benchmark linkers is broadly consistent with reported trends. However, for a complete assessment, it is crucial to account for metal-node effects, topology and volumetric packing. As an illustrative example, while MOF-519 and MOF-520 share the same linker, they differ in coordination environments that in-turn have a significant impact on storage capacities.

Moving to volumetric storage, the goal is to balance porosity with the framework density. Excessively long, lightweight linkers which favor gravimetric storage, will lower the volumetric capacity due to reduced packing density. Accordingly, a balanced reward function needs to account for: (**i**) anchor – anchor span; (**ii**) rigidity; (**iii**) symmetry of the linker (or a proxy-thereof); and (**iv**) intermediate molecular weights that avoid excessively heavy (reduces gravimetric capacity) and light (reduces volumetric capacity) linkers. The balanced gas storage reward (R_{gas}) is defined as follows:

$$R_{gas} = \beta_1 D(d_{anchor-anchor}) + \beta_2 D\big(d_{rigidity}\big) + \beta_3 D\big(d_{symmetry}\big) + \beta_4 D(d_{MW}) \tag{6.4}$$

where $d_{symmetry}$ is a measure of similarity of the chemical environment around each anchor using Morgan fingerprints (a proxy for linker symmetry) and d_{MW} is the molecular weight of the linker passed through a Gaussian desirability function that penalizes extremely heavy and lightweight linkers while rewarding linkers with intermediate molecular weights (**Figure S13 a**). Each component is bounded between $[0.05, 0.95]$, and the parameters $\beta_1 - \beta_4$ are tunable. R_{gas} is similarly bound between $[0.05, 0.95]$, with higher values suggesting an increased applicability for gas storage. We note here that several parameters (especially the Gaussian desirability function) may need to be tuned to the system of interest.

The parameters $\beta_1 - \beta_4$ were empirically optimized against known linkers belonging to high-performing MOFs, with weights selected to preserve qualitative ranking of linkers corresponding to benchmark MOFs. At values of β_1 = 0.2; β_2 = 0.2; β_3 = 0.4 and β_4 = 0.2, the reward score trends were found to reasonably correlate to experimental data. **Figure S13 c** shows the distribution of R_{gas} values using these parameters when evaluated on 25,000 molecules from the training dataset. The distribution is broad (with a median at 0.43) and similar to the R_{grav} distribution, does not saturate at either extreme. There is a weak bimodal character, with a secondary shoulder at higher values, suggesting a partitioning of the linker space into two regimes. The larger class is geometrically sub-optimal (**Figure S13 b**), while the smaller class is primarily driven by symmetry and rigidity components of the score. This is by design, considering that reticular assembly of frameworks of interest favor linkers with symmetric anchor environments and lower conformational flexibility, yet having a reasonable span.

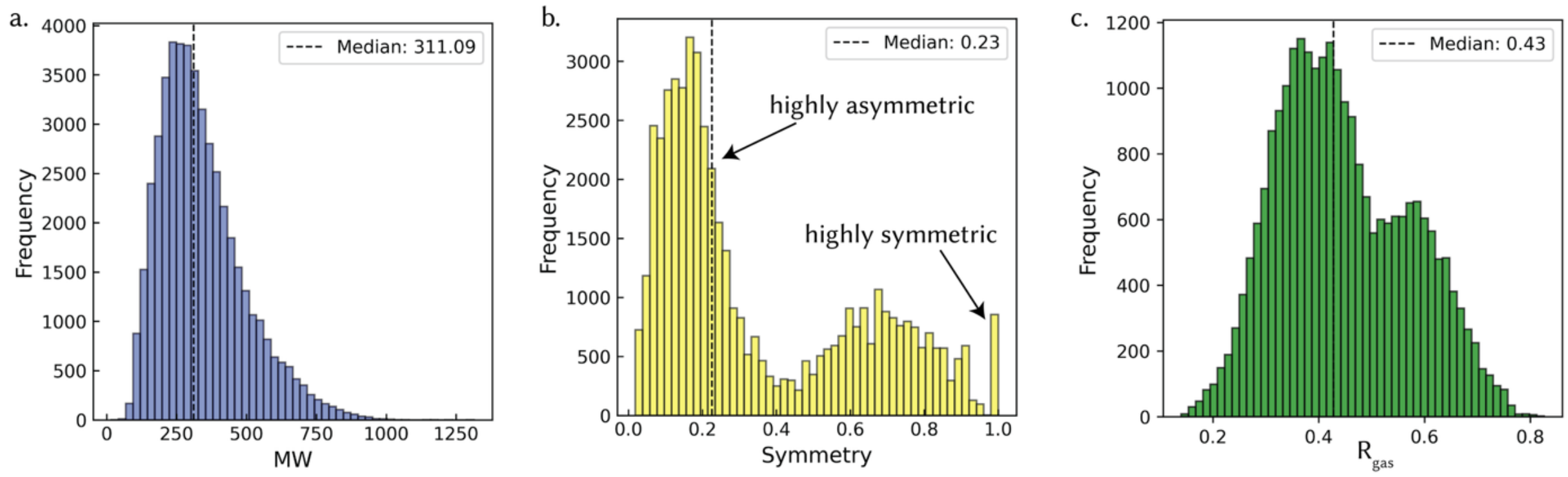

Figure S13. Distribution of the molecular weight of molecules from the training dataset (n = 50000). The distribution follows an expected trend with a mode around 250 g mol^{-1}, median of 311 g mol^{-1} and a long tail that extends past 1250 g mol^{-1}. Ideal linkers for gas storage should adopt intermediate molecular weights – i.e., not too light to favor low framework densities yet not too heavy to favor high densities. We thus define a Gaussian desirability function (centered around 320 g mol^{-1}) with a width of 140 g mol^{-1} that rewards these intermediate values while penalizing extremes. **b.** Distribution of the $d_{symmetry}$ and **c.** R_{gas} scores of molecules from the training dataset (n = 25,000). $d_{symmetry}$ has a strong bimodal distribution suggesting the partitioning of the linker space into two classes: linkers with sub-optimal symmetry and linkers with optimal symmetry. Only 1.6% of the linkers attain the maximum $d_{symmetry}$. For R_{gas}, the distribution is broad (median = 0.43) with a weak bimodal character. Neither extreme is saturated – confirming that there is no collapse. The weak bimodal character stems from the partitioning of the linker space described above. Here, the smaller class indicates more favorable symmetry and rigidity yet having a reasonable span and molecular weight.

Figure S14 shows the R_{gas} scores computed for linkers corresponding to some of the top-performing MOFs for gravimetric CH_4 storage. Again, the scores assigned to each linker correlate well with the observed trends. In this instance, the linker of Al-soc-MOF-1[21] shows a higher relative R_{gas} score compared to the linker of CU-6. The linker in PCN-14 shows the highest relative score; while the MOF itself is not the best performing, its performance is competitive, nonetheless. Yet again, we stress that there are several aspects not captured here; we remind the reader that this remains a computationally efficient proxy for discriminating linkers.

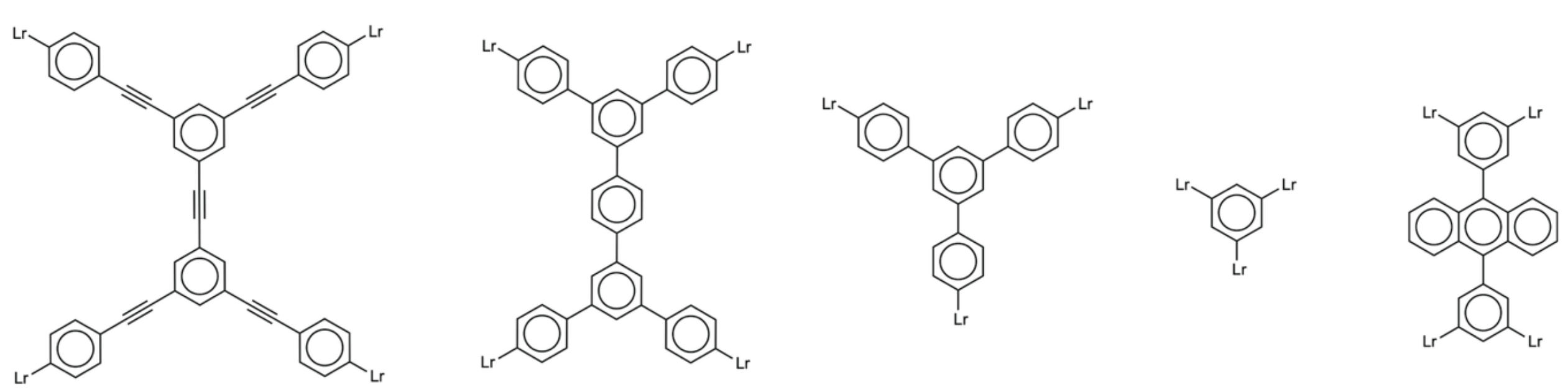

CU-6 (0.73) **Al-soc-MOF-1** (0.73) **MOF-519/-520** (0.61) **HKUST-1** (0.64) **PCN-14** (0.81)

Figure S14. R_{gas} scores for linkers belong to top-performing MOFs for CH_4 storage. There is a reasonable correlation between the score assigned to the linker and the reported gravimetric and volumetric storage capacities of the MOF. However, for a complete assessment, it is crucial to account for metal-node effects, topology and volumetric packing.

S6.2. Biocompatibility. For passive drug delivery applications, the goal is not only to identify synthetically plausible linkers but to prioritize chemotypes with molecular and structural characteristics associated with safety, solubility, and functional performance after assembly. Consistent with the gas-storage analysis above, we preserve the geometric approach of linker-level screening while introducing safety, performance and compatibility controls that are relevant to biomedical deployment.

The rationale of this reward function is again, modularity, focusing on: (**i**) safety terms penalizing linker chemistries or fragments associated with high acute-toxicity risk; (**ii**) performance emphasizing linker span, solubility and accessible porosity properties for loading, which include pore-limiting diameter (PLD) and accessible surface area (ASA) or derived approximations if unavailable; (**iii**) benign-linker terms suppressing strongly interacting or reactive motifs that can perturb therapeutic cargo; (**iv**) stability prioritizing candidates less likely to trigger burst release, premature linker failure, or charge-driven instability under physiological conditions; finally (**v**) penalty element penalizing linkers with known toxicity warnings and extreme charge states. We define a biocompatibility objective as follows:

$$R_{bio} = \gamma_1 S + \gamma_2 P + \gamma_3 B + \gamma_4 T - \Pi \quad (6.5)$$

where *S*, *P*, *B*, and *T* denote safety, performance, benign-linker behavior, and stability sub-scores, respectively, and Π is an explicit penalty for warnings and extreme charge states. All scores and components range between $[0,1]$. The biocompatibility reward is formulated such that it can operate at either the framework or linker level. Where information on the metal node and assembled framework is available, the complete linker- and framework-level descriptors are evaluated; where such information is unavailable, the reward is evaluated only using linker-level descriptors.

The safety block (*S*) combines: (**i**) metal-node safety from curated median lethal dose (LD50)-based priors reported by Menon and Fairen-Jimenez,[22] (**ii**) toxicity-space proximity derived from acute-toxicity chemistry, and (**iii**) formal-charge safety,

$$S = s_1 . M_{metal-toxicity} + s_2 . T_{tox-space} + s_3 . Q_{charge} \quad (6.6)$$

The toxicity-space term was built from descriptor and fingerprint neighborhoods obtained from LD50 readouts for 35,300 compounds in TOXRIC Database.[23] A 55-feature standardized numeric manifold (RDKit descriptors) and ECFP4 fingerprint manifold (2048 bits) were blended by learned block weights: numeric 0.62, fingerprint 0.38. Toxicity severity was weighted across United Nations Globally Harmonized System of Classification and Labelling of Chemicals (UN GHS) classes (Class 1 as the most severe to Class 5 associated with least toxic compounds) so that proximity (k-NN) to highly toxic regions is penalized more strongly.

Toxicity-space was qualitatively evaluated using several MOF linkers and other chemicals that were reported to have been experimentally tested, including PCN-222 (Tetrakis (4-carboxyphenyl) porphyrin (TCPP) linker), UiO-66 (terephthalic acid linker), NU-1000 (Pyrene tetrabenzoic acid) and ZIF-8 (2-methylimidazole, featuring known LD50 oral rat toxicity at ∼1,400 mg kg^{-1}).

The performance block (*P*) combines linker length, solubility, and surface/loading proxies,

$$P = p_1(d_{anchor-anchor}) + p_2(d_{solubility}) + p_3(d_{loading}) \quad (6.7)$$

where linker length ($d_{anchor-anchor}$) is estimated from anchor-to-anchor separation and heavy-atom span; $d_{solubility}$ is encoded using LogS together with a bounded LogP window; $d_{loading}$ is a surface/loading proxy which uses accessible surface area (ASA) and pore limiting diameter (PLD) when available, with deterministic

linker-level proxies otherwise. Pore accessibility and packing constraints remain fundamental for biocompatibility applications as well.

The benign-linker block (B) combines formal-charge moderation, a payload-interaction window (LogP and TPSA), and structural alert burden (PAINS/BRENK/NIH),

$$B = b_1(d_{charge}) + b_2(d_{interaction}) + b_3(d_{burden}) \quad (6.8)$$

This function penalizes reactive or otherwise undesirable linker chemistries that may increase the likelihood of unfavorable payload-framework interactions. We also note that charge contributes to both safety and benign terms by design: the safety block penalizes extreme formal-charge states, whereas the benign block captures the payload interaction fit. Structural burden (d_{burden}) is estimated by calculating normalized count of reported warning matches for the fragments of the generated structure in either (**i**) PAINS (pan-assay interference); (**ii**) BRENK (reactive, toxic, or unstable fragments) or (**iii**) NIH (problematic functional groups based on NIH medicinal chemistry guidelines).

The stability block (T) integrates metal stability, linker rigidity/symmetry, solubility-mediated dispersion behavior, and charge-release stability,

$$T = t_1(d_{metal-stability}) + t_2(d_{linker-stability}) + t_3(d_{solubility}) + t_4(d_{release}) \quad (6.9)$$

Solubility is included as a relevant molecular descriptor for aqueous compatibility and linker behavior under physiological conditions. Metal stability and safety are obtained from the curated dataset in Menon and Fairen-Jimenez,[22] while the stability of the linker is determined based on rigidity and symmetry, similar to the reward function for gas storage in **Section S6.1** above.

During method development, Bayesian optimization was used to tune component and subcomponent weights under constrained known validation linker samples. The design objective combined toxicity-aware ranking constraints (to suppress high scores in severe toxicity classes), smooth score spread across viable chemotypes, and robustness across repeated sampled subsets. Final production weights were selected from the stable region identified across these runs and then fixed to preserve deterministic scoring as $\gamma_1 = 0.60, \gamma_2 = 0.14, \gamma_3 = 0.07, \gamma_4 = 0.19$. Safety here is prioritized and was assigned the highest weightage. Our calculated safety score qualitatively aligns well with expected trends (for instance (highest to lowest safety): terephthalic acid > ethylene glycol > TCPP > 2-methylimidazole > Pyrene tetrabenzoic acid > fentanyl). **Figure S15** shows the distributions of the individual blocks and the distribution of the corresponding composite $\boldsymbol{R_{bio}}$. All distributions remain broad and non-saturated, indicating that the function retains ranking resolution rather than collapsing near hard bounds.

We note that the presented formulation is an initial demonstration of how reward functions can accommodate multicomponent design objectives. Given the inherent complexity of MOF biocompatibility, a comprehensive treatment of biocompatibility requires consideration of additional framework, payload and biological factors that lie beyond the scope of the present study. This formulation therefore serves as proof-of-concept, with a more extensive development and validation reserved for a future study.

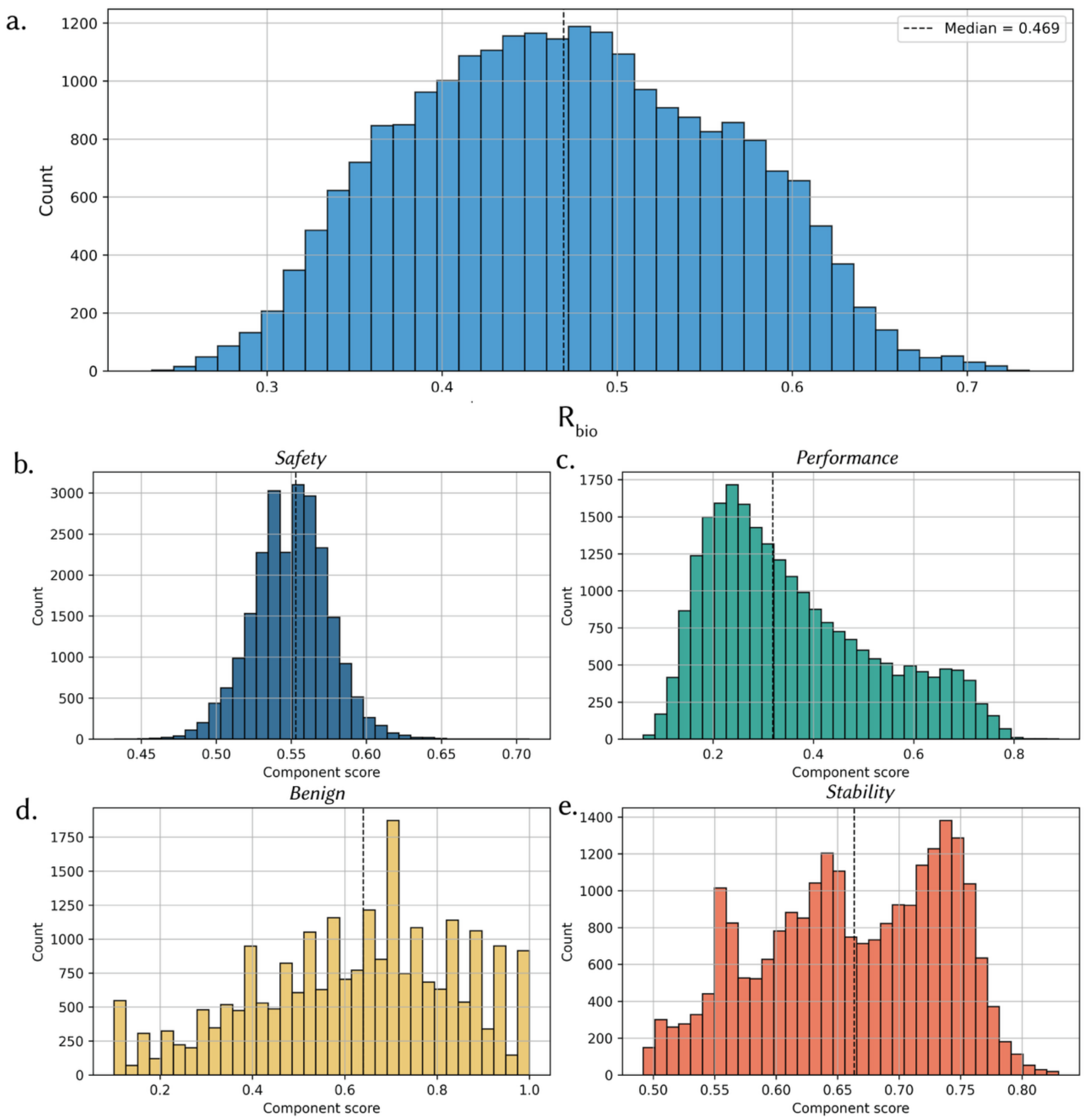

Figure S15. a. Distribution of the R_{bio} scores of linkers from the training set (n = 25,000) of which 93.76% produced valid scores after invalid SMILES cleaning/parsing. The total score distribution remains broad (with median = 0.469), indicating non-collapsed ranking capacity for molecular optimization. **b - e.** Distributions of individual components scores for **b.** safety (*S*); **c.** performance (*P*); **d.** benign-linker score (*B*) and **e.** stability (*T*). Each of these distributions is also non-collapsed to either extreme. We further impose rigid constraints during the design process, such as rejecting molecules with safety scores < 0.50.

Section S7. Design modes

Nexerra[R1] supports two distinct design modes for linker generation: (**i**) Direct design and (**ii**) Scaffold-constrained design. These design modes differ in how the latent variables are sampled and how geometric constraints are imposed. In practice, the direct design mode works best for 2-connected linkers and on nets with less strict topological constraints – for instance, *fcu* and *pcu* nets. On the other hand, the scaffold-constrained design mode works better for highly-connected linkers and nets with stricter topological constraints – such as *csq*.

S7.1. Direct design. Here Nexerra[R1] generates linkers by sampling the local neighborhood around a user-specified seed molecule. Given a seed linker x_{seed}, the encoder approximates a posterior distribution,

$$q_{\phi}(z|x_{seed}) = \mathcal{N}\big(z; \mu_{seed}, diag(\sigma_{seed}^2)\big) \tag{7.1}$$

Where $\mu_{seed} = \mu_{\phi}(x_{seed})$ and $\sigma_{seed} = \sigma_{\phi}(x_{seed})$. To control the radius of the neighborhood, we sample embeddings from a scaled neighborhood of the posterior, i.e,

$$z = \mu_{seed} + \gamma\sigma_{seed} \cdot \epsilon, \ \ \epsilon \sim \mathcal{N}(0, I) \tag{7.2}$$

Where $\gamma > 0$ is a user-defined neighborhood radius. The smaller the γ, the more conservative the exploration while larger γ values lead to more diverse molecules with respect to the seed. Each latent embedding is subsequently decoded autoregressively,

$$x \sim p_{\theta}(x|z) \tag{7.3}$$

using temperature-scaling and nucleus sampling as described in **Section S2.4**. Generated molecules are then filtered first using strict chemical filters, followed by application-specific reward functions (see **Section S6**). The direct design mode thus enables rapid exploration of the local chemical neighborhood of seeds without imposing any scaffold constraints.

Figure S16 shows the direct design method applied to several different classes of bidentate MOF linkers. These linkers post-generation are subjected to strict chemical filters to discard structures which are entirely implausible or highly synthetically complex. Here we show a subset of generated linkers with R_{grav} scores > $0.7 \times R_{grav}$ values of the seed.

S7.2. Scaffold-constrained design. Here the design is constrained around a pre-defined molecular core (or scaffold) that contains defined connecting handles (again denoted with the '[Lr]' token for ease of processing). The goal of this mode is to preserve this scaffold – which is otherwise not possible in the direct design mode. Given a seed linker x_{seed}, we first decompose the linker as,

$$x_{seed} = \big(x_{scaff}, x_{arm}\big) \tag{7.4}$$

The design method is agnostic to the number of arms, however, this number is preserved during the design process. In other words, this design mode does not change the connectivity of the input seed. After this decomposition, we apply the direct design method to x_{arm} (see $7.1 - 7.3$).

Once new arms are generated, these are symmetrically grafted onto the core. First, we identify the anchor sites on the scaffold, denoted by the '[Lr]' token. Here, the connection site is the atom directly bonded to '[Lr]'. Once these atoms are identified, the '[Lr]' atoms are deleted from the scaffold. From the arm (which is ditopic), we delete one '[Lr]' atom, while preserving the other one to indicate node-coordination moieties. We then attach this processed arm by forming a single bond between the scaffold connection site and the arm connection site. We then repeat this process for every connection site on the core. Finally, we perform a force-field optimization of the linker and carry out sanitization. As an illustrative example, **Figure S17** shows this design mode applied to the porphyrin-core.

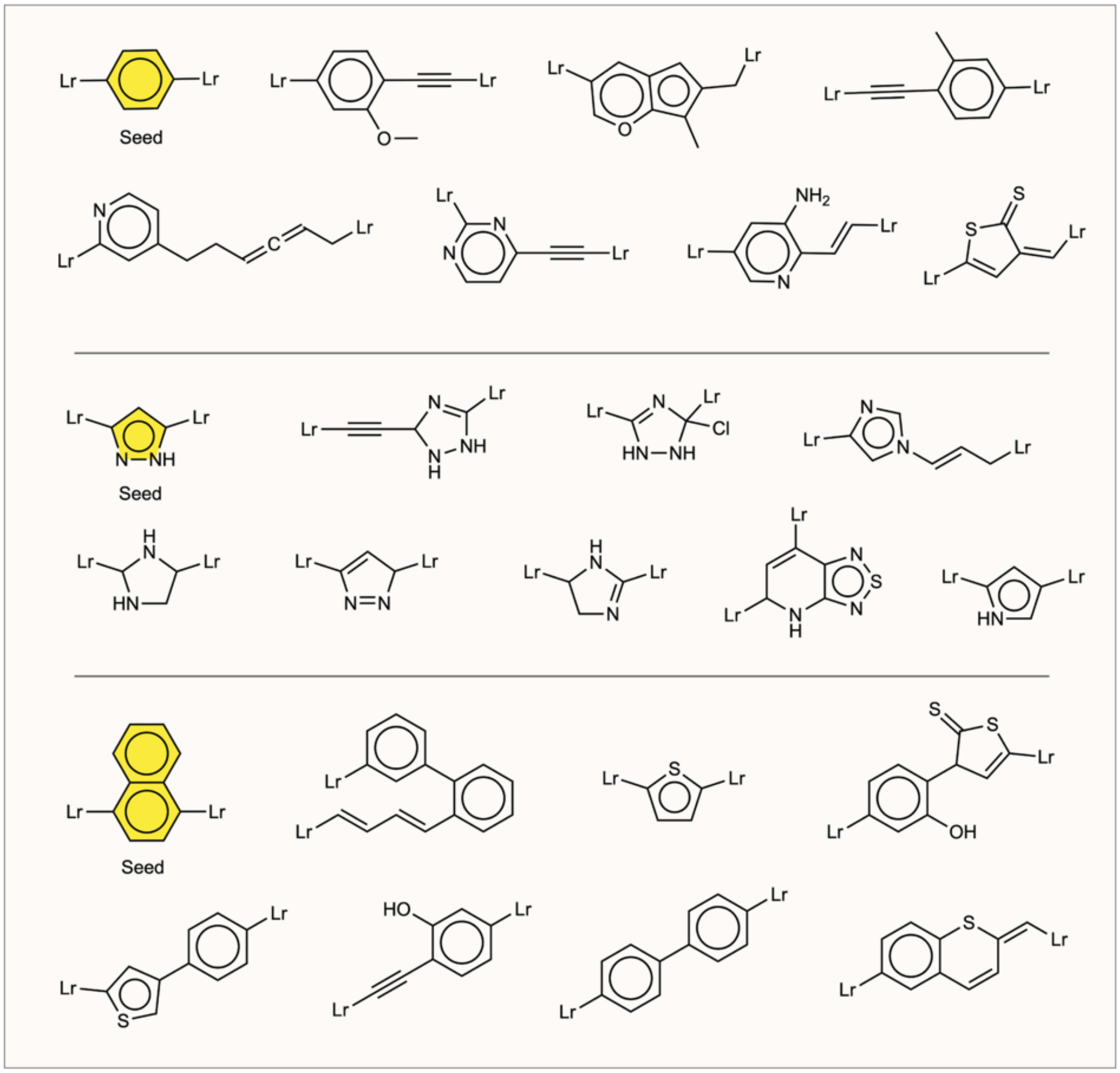

Figure S16. Direct design mode applied to different classes of bidentate MOF linkers. The seeds are highlighted in yellow.

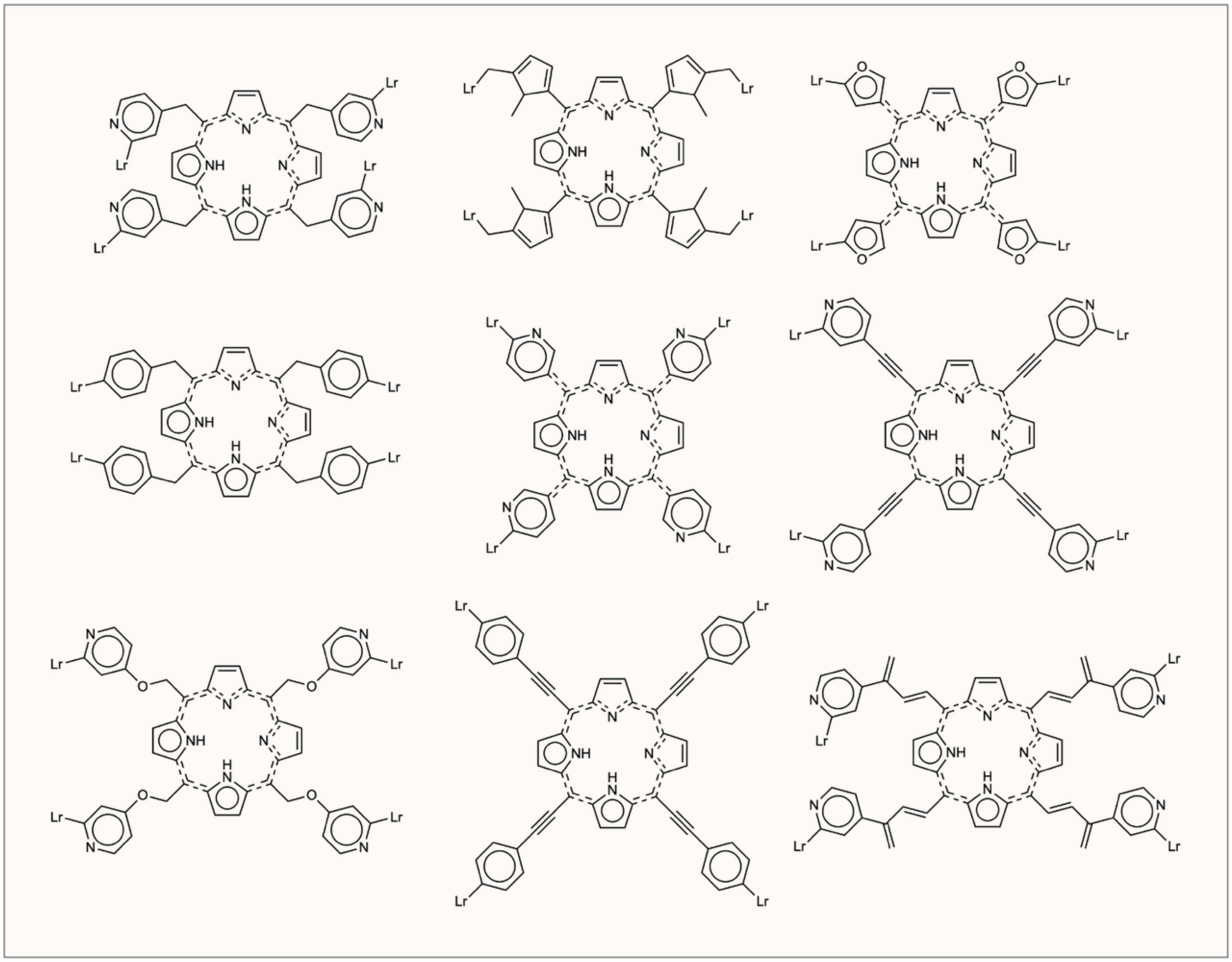

Figure S17. Scaffold-constrained design mode applied to the porphyrin-core.

Section S8. Experimental section

S8.1. Synthetic procedure

Synthesis of Linker (L). The synthesis of L (**Figure S18**) follows a previously reported procedure.[24]

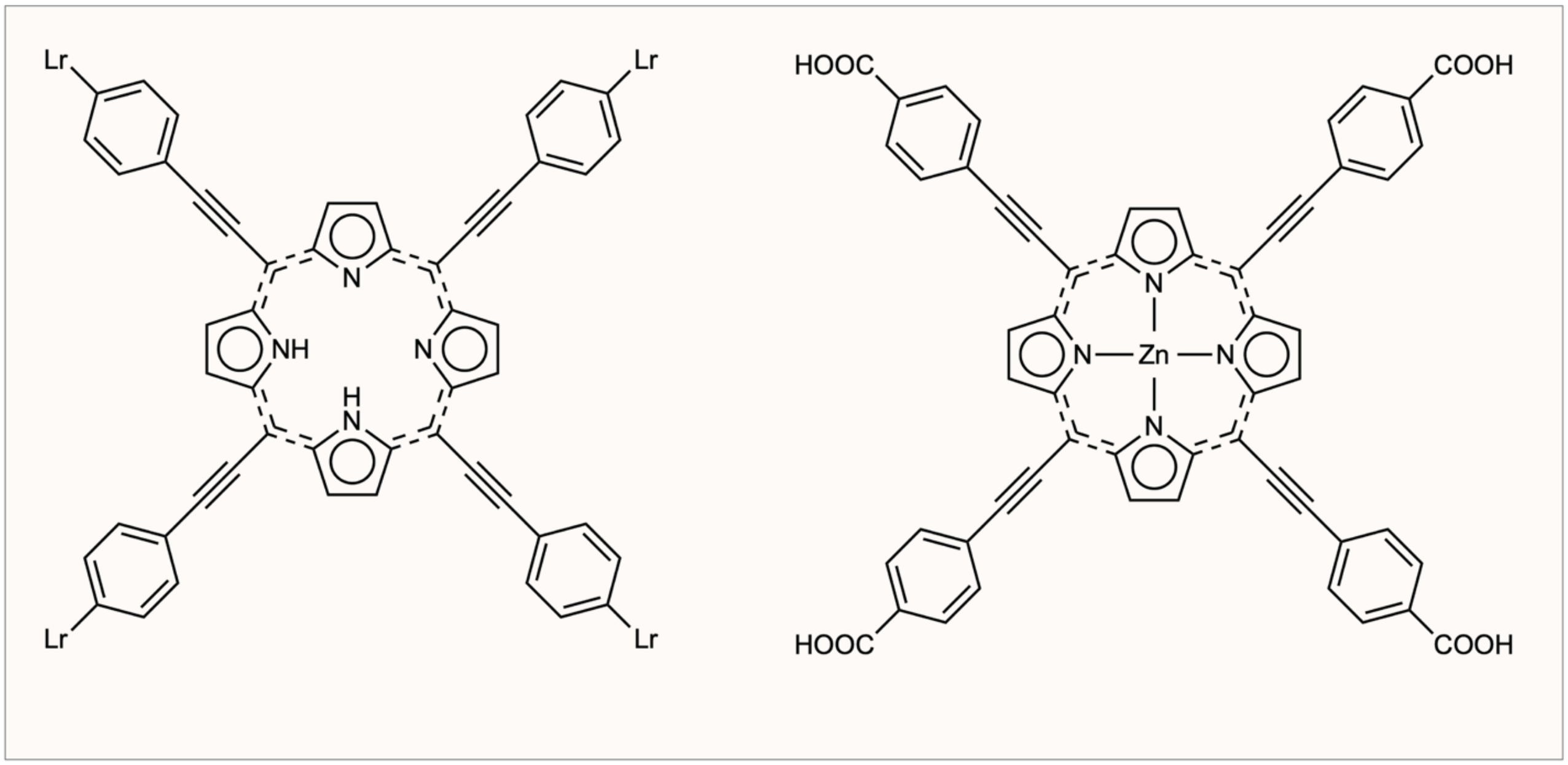

Figure S18. The porphyrin linker designed using Nexerra[R1] (left) and the linker (L) used for the synthesis of CU-525.

Synthesis of CU-525. $HfCl_4$ (100 mg), H_4L (50 mg) and benzoic acid (1.25 g, 7.49 mmol) were dissolved in a mixture of DMA (25 mL) and HOAc (5 mL) through sonication. The resulting solution was sealed and heated in a 120 °C oven for 48 h. Black cubic crystals were formed.

S8.2. Single Crystal X-ray Crystallography

Single crystal X-ray diffraction measurements were performed on the *PROXIMA 2A* micro-focused beamline at synchrotron SOLEIL (λ = 0.729319 Å) using an EIGER X 9M 2D hybrid photon counting detector. X-ray data were collected at 100 K. Data integration and reduction were undertaken with *Xia2*.[25] No corrections for solvent scattering have been made. The structure was initially solved with SHELXT[26] and refined by full-matrix least squares refinement on F^2 by the SHELXTL-2014[27] using the Olex2-1.5[28] software package. Standard crystallographic methods were used where necessary, including constraints, restraints, and rigid bodies. All carbon-bound hydrogen atoms were added in idealized positions and refined using a riding model. Restraints were used to obtain reasonable parameters. H-atoms were refined isotropically, while the other atoms were refined anisotropically. Crystal data and details of the X-ray data collection are given in **Table S1**.

Table S1. Crystal data and structure refinement for CU-525

	**CU-525**<br>CCDC: 2514566
Formula	$C_{38.67}H_{54.67}HfN_{7.33}O_{5.33}Zn_{0.5}$
F. W. (g mol^{-1})	918.73
Temperature/K	100
Wavelength/Å	Synchrotron (λ = 0.72932)
Crystal system	Cubic
Space group	*Pm-3m*
$a = b = c$/Å	22.7093(2)
$\alpha = \beta = \gamma$ /°	90
Volume/Å^3, Z	11711.5(3) 6
ρ_{calc}g/cm^3	0.782
μ/mm^{-1}	1.608
F(000)	2806.0
2Θ range for data collection/°	2.602 to 54.206
Reflections collected	117300
Independent reflections	2228 [R_{int} = 0.1933, R_{sigma} = 0.0858]
Data/restraints/parameters	2228/110/68
Goof	0.894
Final R indexes [I>=2σ (I)]	R_1 = 0.0928, wR_2 = 0.2712
Final R indexes [all data]	R_1 = 0.1193, wR_2 = 0.2811
Largest diff. peak/hole / e Å^{-3}	2.60/-2.48

S8.3. Comparison of structural models. To assess the structural fidelity of the computational assembly, we compare the experimentally resolved single-crystal structure of CU-525 against the assembled structural model of $\phi - 11$. Since the isolated yield of CU-525 was insufficient to obtain a high-quality experimental PXRD pattern, the powder diffraction comparison shown below is performed between the experimentally resolved crystal structure and the corresponding computational model.

Figure S19 compares the structural model obtained from the topological assembly of the Nexerra[R1]-generated linker ($\phi - 11$) with the experimentally resolved single-crystal structure of CU-525. The model structure is assembled using the Zr_6-oxo cluster (consistent with MOF-525), while CU-525 is synthesized experimentally using an Hf_6-oxo cluster. Due to nearly identical coordination geometry, this substitution is not expected to alter the underlying net. At a global topology and connectivity level, $\phi - 11$ reproduces the coordination environment and node connectivity of CU-525; however, local differences are observed in some linker arms. These arise because the assembly algorithm selects an alternate but symmetry-equivalent orientation – essentially 'flipping' the placement of the arm. This occurs because the arm placement is geometrically invariant and the algorithm is

unable to discriminate between the alternate placement conditions. Resolving this would require substantial refinement of the assembly algorithm itself and lies outside the scope of the present work.

Simulated powder X-ray diffraction (PXRD) patterns of $\phi - 11$ and CU-525 are also shown in **Figure S19**. Both patterns are in close agreement with each other, with no additional peaks observed in the generated structure. We however observe a slight systematic shift in the peak positions of the generated structure relative to CU-525 which arise as an effect of structural relaxation. There are differences in relative peak positions as well, which are attributed to variations in either atomic positions or linker conformations. Overall, these observations suggest structural equivalence with minor conformational deviations.

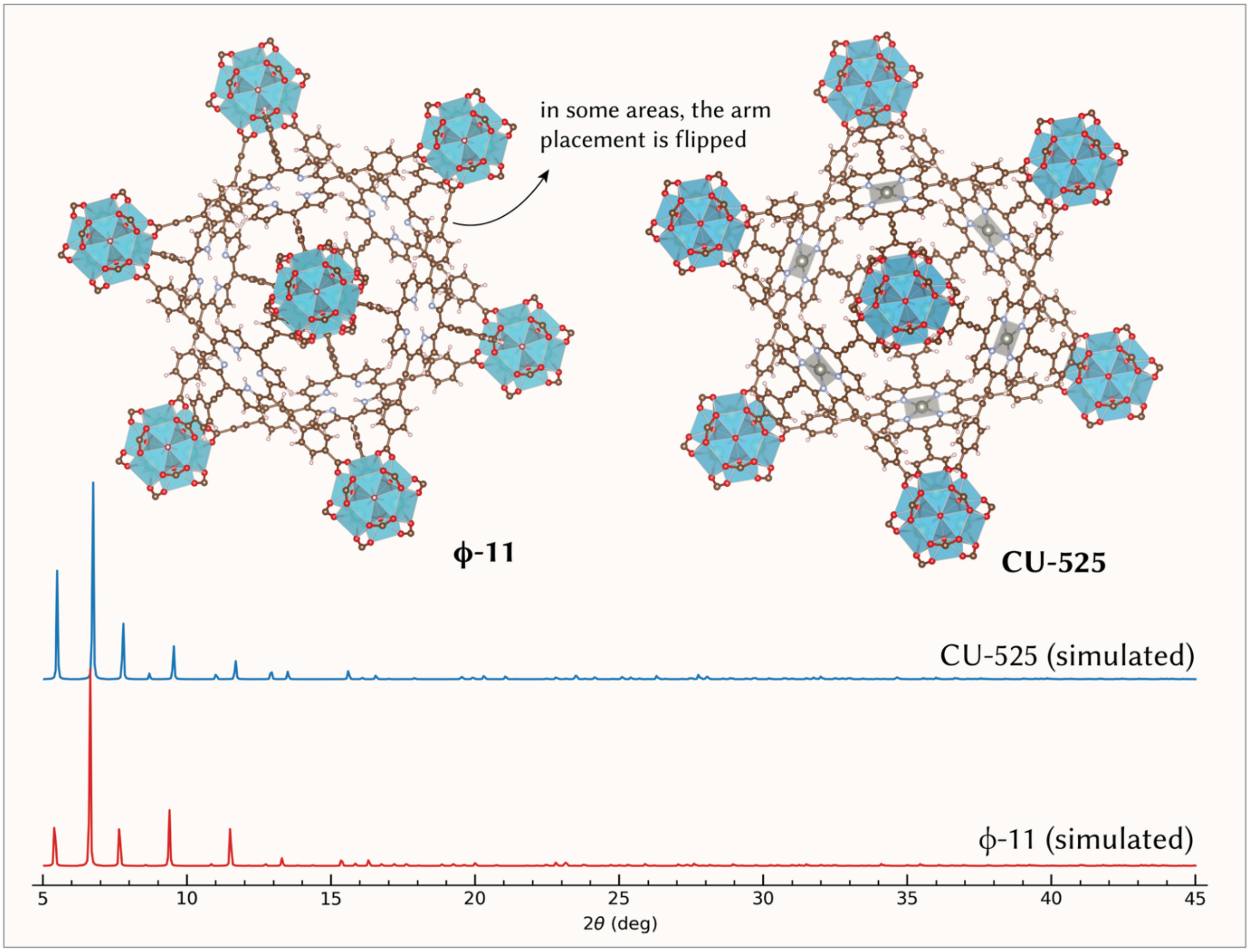

Figure S19. (Top) Optimized structural model of ϕ-11 (left) compared to the structure of CU-525 resolved using SCXRD as described above. We note that the MOF assembly algorithm flips the placement of the arm in certain instances leading to conformational deviations from the experimentally resolved structure. (Bottom) Simulated PXRD patterns from the assembled ϕ-11 model (red) and experimentally resolved single-crystal structure of CU-525 (blue). Please note that the patterns have been max-normalized.

Section S9. Flow model

S9.1. Flow matching formulation. We use the flow-matching formulation introduced by Lipman et al.[29] and extended by Tong et al.[30] Below, we provide only the essential mathematical definitions and refer readers to the original and foundational references for full derivations. Starting with the latent space learnt by Nexerra[R1], let:

$$\{(z_i, y_i)\}_{i=1}^{N},\ z_i \in R^{\mathbb{D}},\ y_i \in R \tag{9.1}$$

denote the latent encodings z_i of the training dataset and y_i the associated scalar property (e.g. anchor – anchor span, logP, or, in principle, any reward function). The latent variables are then standardised as:

$$\mu_z = \frac{1}{N}\sum_{i=1}^{N} z_i\ ,\ \sigma_z = \sqrt{\left(\frac{1}{N}\sum_{i=1}^{N}(z_i - \mu_z)^2 + \epsilon\right)} \tag{9.2}$$

and we define:

$$\tilde{z}_i = \frac{z_i - \mu_z}{\sigma_z} \tag{9.3}$$

The flow model and associated losses are defined in this standardised latent space. This is then inverted during inference. During training, source points are sampled from the empirical latent distribution in the standardized latent space, while target points are sampled from percentile-conditioned subsets. The source and target mini-batches are coupled with the exact minibatch OT-formulation of Tong et al.[30] We define a percentile-based threshold (τ_s) on $\{y_i\}$ such that for sorted property values $y_1 \geq y_2 \geq \cdots \geq y_N$, we have a linear percentile scheduler:

$$P_s = P_{start} + (P_{end} - P_{start}) \times \frac{s}{S} \tag{9.4}$$

where s is the current training step, and S is the total number of training steps. For maximising a property (e.g. length) $P_{end} > P_{start}$ while for minimizing a property (e.g. logP) $P_{start} > P_{end}$. Given $P_s \in [0,\ 100]$, we select an index $k_s = \left[N\left(\frac{100-P_s}{100}\right)\right]$ and define the current threshold as:

$$\tau_s = Y_{(k_s)} \tag{9.5}$$

The conditional target distribution at step s is the empirical distribution over latents whose property exceeds the threshold (τ_s) for property maximization:

$$q_{\tau_s^+}(z)\ \propto\ \sum_{i=1}^{N}\{y_i \geq \tau_s\}\delta(z - \tilde{z}_i) \tag{9.6}$$

For property minimization, we instead use the subset $y_i \leq \tau_s$. For a contrastive unconditional sampling (used for classifier-free guidance, discussed shortly) we define a larger subset of the latent distribution (OR the full distribution) as:

$$q_{uncond}(z)\ \propto\ \sum_{i=1}^{N}\delta(z - \tilde{z}_i) \tag{9.7}$$

We parameterize a continuous normalizing flow (CNF) in the latent space by an ordinary differential equation (ODE):

$$\frac{dz(t)}{dt} = f_\theta(t, z(t), c),\ t \in [0,1] \tag{9.8}$$

where $z(t) \in \mathcal{R}^{\mathcal{D}}$ is the latent state at time t, $c \in \mathcal{R}$ is the scalar conditioning variable and $f_\theta: [0,1] \times R^D \times R \to R^D$ is the neural net vector field. We implement this vector field as a neural network NN (defined in **Section S9.2**) operating on the concatenation of the latent (z), time embedding (t) and condition (c):

$$f_\theta(z, t, c) = NN([z, \gamma(t), c]) \tag{9.9}$$

where $\gamma(t) \in R^{2d_{Fourier}}$ is a Fourier feature embedding of time – $\gamma(t) = (sin(2\pi Bt),\ cos(2\pi Bt))$ – similar to the positional encoding performed on the training data before passing it to the transformer encoder. $B \in R^{d_{Fourier}}$ is a random frequency vector ($d = 64$). For a fixed condition c, integrating this ODE from $t = 0 \to t = 1$ defines a flow map:

$$\Phi_\theta^c: R^D \to R^D,\ z_1 = \Phi_\theta^c(z_0) \tag{9.10}$$

This flow map transports samples from the empirical latent distribution towards the conditional distribution $q_\tau(z)$.

We train f_θ using the exact optimal transport-conditional flow matching (OT-CFM) between the empirical latent distribution and the conditional (OR unconditional) empirical latent distribution.[30] For a training sample (s), we sample the base latents: $z_0 \sim q_{emp}$, conditional target: $z_1^{cond} \sim q_{\tau_s}$ and unconditional target: $z_1^{uncond} \sim q_{uncond}$. These are paired with corresponding conditions: $c^{cond} = \tau_s$, $c^{uncond} = c_{uncond} \leftarrow$ (in our case $\emptyset = 0.0$). In order to train both conditional and unconditional flows in a single network, we apply conditional dropout with probability p_{drop}. For each sample in the mini-batch, we choose:

$$P[(z_1, c) = (z_1^{\text{uncond}}, c_{\text{uncond}})] = p_{\text{drop}},$$
$$P[(z_1, c) = (z_1^{\text{cond}}, c_{\text{cond}})] = 1 - p_{\text{drop}}. \tag{9.11}$$

For each pair $(z_0,\ z_1)$, we define the displacement interpolation (the OT geodesic in Euclidean space):

$$x_t = (1 - t)z_0 + tz_1,\ t \in [0,1] \tag{9.12}$$

Under the quadratic cost OT formulation, the exact conditional OT flow along this path has a constant velocity:

$$v^*(z_0,\ z_1) = z_1 - z_0 \leftarrow \text{t independent} \tag{9.13}$$

OT-CFM leverages this formulation to construct a simple closed-form target vector field. The training objective minimizes the discrepancy between the model vector field and this OT-flow at random intermediate times:

$$\mathcal{L}_{OT-CFM}(\theta) = \mathbb{E}_{z_0 \sim q_{emp}, (z_1, c) \sim q_s, t \sim \mathcal{U}(0,1)} ||f_\theta(t, x_t, c) - (z_1 - z_0)||_2^2 \tag{9.14}$$

where q_s is a mixture over conditional and unconditional latent samples. The training is performed in mini-batches and t is sampled *i.i.d.* from $\mathcal{U}(0,1)$ per example.

During sampling, we use classifier-free guidance (CFG)[31] to bias the flow model towards high-threshold conditions without having to train a separate classifier. Given a desired threshold $c^* = \tau^*$ and a CFG scale – $m \geq 0$, we define conditional and unconditional vector fields at a point (t, z) as:

$$v_{uncond}(t, z) = f_\theta(t, z, c_{uncond}),\ v_{cond}(t, z; c^*) = f_\theta(t, z, c^*) \tag{9.15}$$

The guided vector field is then:

$$v_{CFG}(t, z; c^*, m) = v_{uncond}(t, z) + m\big(v_{cond}(t, z; c^*) - v_{uncond}(t, z)\big) \tag{9.16}$$

We generate samples by solving the guided ODE:

$$\frac{dz(t)}{dt} = v_{CFG}(t, z(t); c^*, m) \tag{9.17}$$

from $t = 0 \rightarrow t = 1$. The ODE is integrated with an adaptive Dormand-Prince solver. For $m = 0$, this is reduced to an unconditioned model and for $m = 1$, this is the plain conditional model. $m > 1$ amplifies condition-dependent output.

S9.2. Neural network architecture. The core network is a stack of N residual blocks (*ResBlocks*).[32] For our implementation, we use $N = 8$. Each block uses FiLM conditioning.[33] Given a block input $x \in \mathrm{IR}^H$, we first apply a LayerNorm (LN):

$$\tilde{x} = LN(x) \tag{9.18}$$

A FiLM module produces per-feature scale and shift parameters $(\gamma, \beta) \in \mathrm{IR}^H$ from the conditioning vector:

$$(\gamma, \beta) = FiLM(h_c), \;\; FiLM: \mathrm{IR}^H \rightarrow \mathrm{IR}^{2H} \tag{9.19}$$

Where h_c is a hidden conditioning vector. The normalized activations are then modulated per-feature as,

$$\hat{x} = \tilde{x} \odot (1 + \gamma) + \beta \tag{9.20}$$

These are then passed through an MLP with SiLU activations. The FiLM layer is intended to improve the stability of the deep residual stacks. Following this, we add LN and linear projection back to the latent dimension.

S9.3. Training, evaluation and inference. Nexerra[R1]-Flow was trained over 200,000 steps with evaluation every 20,000 steps. At each evaluation, 5000 samples were generated and shift in the property distribution and transport fidelity were logged. **Figure S20** shows the corresponding training logs. The transport accuracy was quantified via relative RMSE (or rRMSE) and mean cosine similarity between predicted and reference latent displacements. Across the training steps, rRMSE increased moderately from ~ 0.02 to ~ 0.035 (~ 0.030 at final checkpoint), while cosine similarity decreased gradually from ~ 0.97 to ~ 0.92 – 0.94. These trends are reflective of a progressively stricter percentile scheduler – i.e., the target slice moving toward higher percentiles. As the target to meet gets stricter, the required displacement increases in magnitude. Importantly, cosine similarity remained high (> 0.92) throughout training, indicating strong directional alignment of the learned vector field with the reference OT displacement.

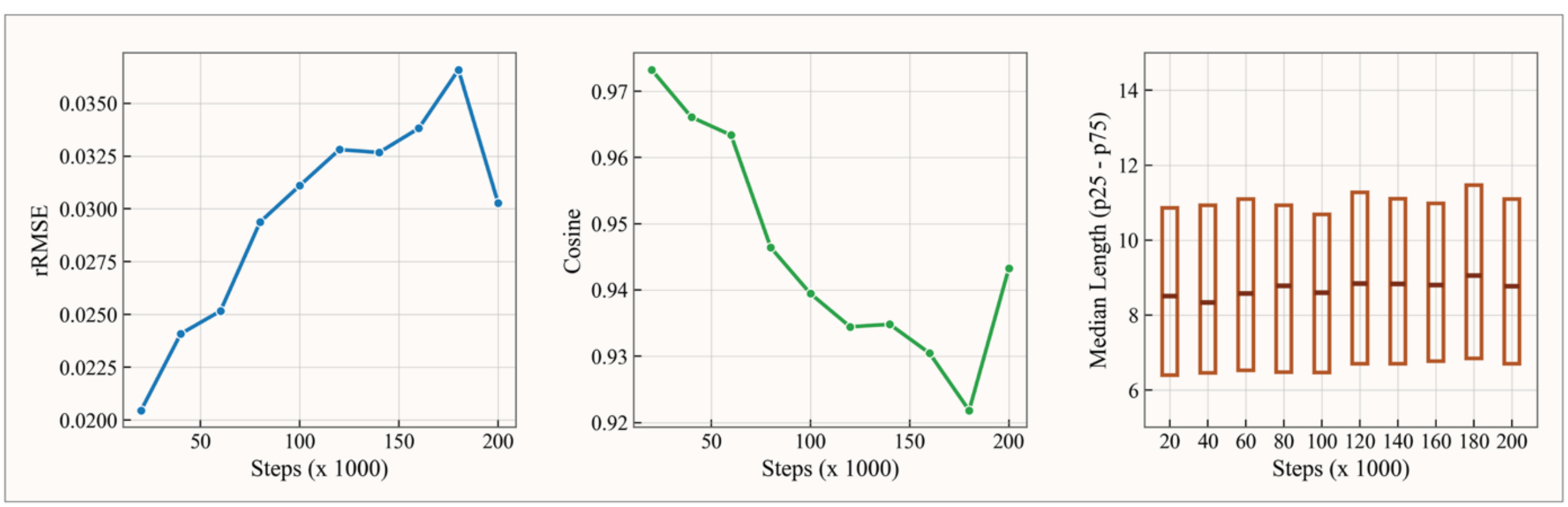

Figure S20. (Left to right). rRMSE of predicted and target latent displacement; mean-cosine similarity between the predicted and target latent displacements; median anchor – anchor span for n = 5000 sampled molecules from the model checkpoint. Please note: during training the target is monotonically increased from the 70th percentile to the 95th percentile – i.e., the training gets progressively harder as the steps increase, which reflects in the growing rRMSE and dropping cosine.

Sampled molecules exhibited a systematic upward shift in the target property distribution (here the anchor – anchor span) over training. The median span increased, while the interquartile range remained stable across checkpoints. This indicates redistribution of probability mass toward higher-percentile regimes without collapse. Notably, at

step 180,000 we recorded the highest relative median anchor-anchor length of 9.05 Å. We thus, selected this checkpoint for further production. Overall, training dynamics are stable and consistent with successful optimal-transport-based conditional generation.

During inference, we initialize the latent trajectory from a stochastic interpolation between the encoded seed linker and isotropic Gaussian noise, $z_0 = \alpha z_{seed} + \sqrt{1-\alpha^2}\epsilon$, where $\epsilon \sim \mathcal{N}(0, I)$. The parameter $\alpha \in [0,1]$ controls the strength of seed retention, i.e., larger values keep samples closer to the seed latent, while smaller values increase exploration.

S9.4. Flow generated linkers and structures. We shift our focus to linkers and their corresponding structures generated using the trained flow model. Each optimized structure has been appended to the data supporting this manuscript. Starting with the BDC linker as a seed, **Figure S21** shows sample linkers generated by Nexerra[R1]-Flow subject to strict geometric filters to ensure compatibility with the target *fcu* net. The main filter implemented here was restricting selection to those linkers whose anchor – anchor angle > 160^0 – i.e., a linearity constraint.

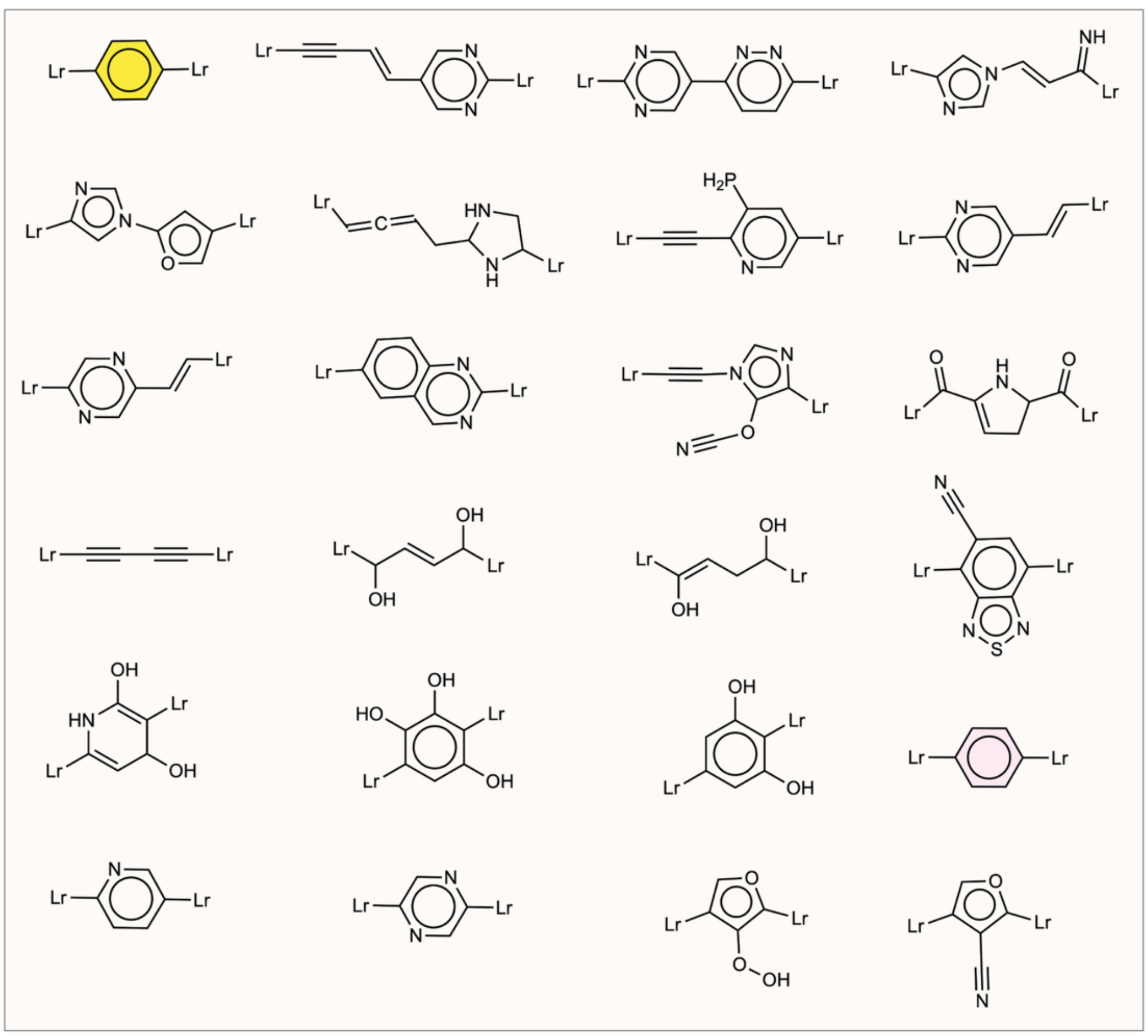

Figure S21. A subset of linkers generated by Nexerra[R1]-Flow using a BDC seed (yellow). These linkers were selected post-filtering for geometric compatibility with the *fcu* net. The linker highlighted in pink is a rediscovery of the input seed.

Figure S22 shows optimized structures generated using these linkers on an *fcu* net using a Zr_6-oxo cluster. While in most cases structural assembly was successful, we observed instances where assembly fails – most commonly,

due to overlapping atoms, or additionally coordinating moieties close to the placed handles. Success rates however depend on topological and geometric complexity of the target net.

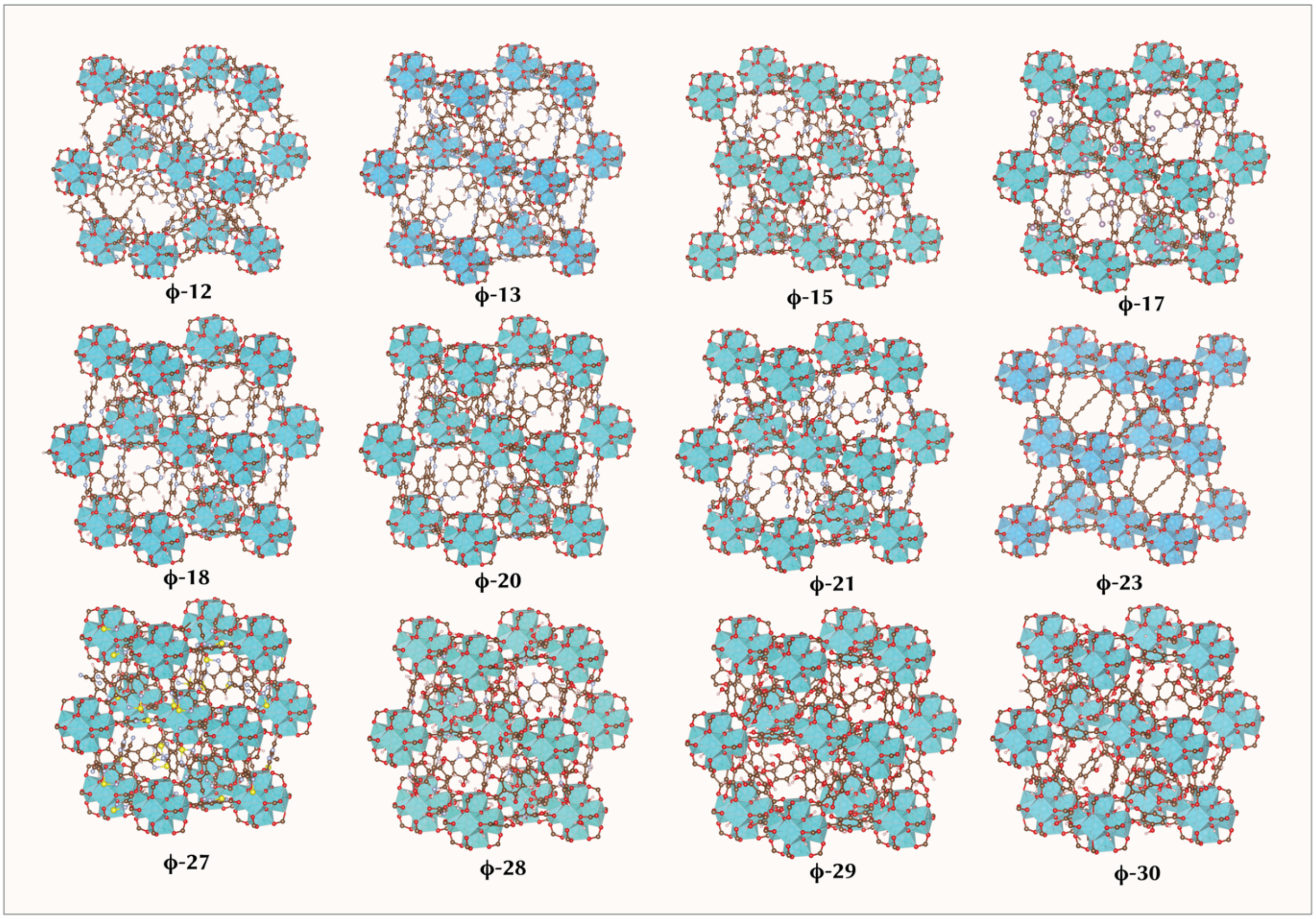

Figure S22. A subset of optimized structures assembled on the *fcu* net from linkers generated from Nexerra^{R1}-Flow.

S9.5. Flow-guided linker elongation relative to baselines. To evaluate whether the flow-guided latent transport framework explores linker regimes beyond those typically accessible through local enumeration strategies, we compared Nexerra^{R1}-Flow against constrained baseline workflows using representative experimentally reported linkers. Specifically, baseline populations were generated using random functionalization and enumeration-based modifications while preserving anchor compatibility constraints. Simply put, the experimentally reported linkers were subject to repeated random functionalization from a fixed library of substituents. Nexerra^{R1}-Flow populations were generated under matched sampling budgets using progressively stronger transport strengths (α = 0.5-0.9). **Figure S23** summarizes the mean linker spans obtained for representative seeds spanning several experimentally relevant reticular linker classes. Across most systems investigated, Nexerra^{R1}-Flow systematically shifted the sampled linker populations toward longer-span regimes relative to the corresponding baseline workflows. It should be noted that seeds that are already long undergo more modest shifts. The extent of the observed distributional shift depended on both the seed linker and transport strength. Increasing α progressively biased sampling toward increasingly elongated linker populations, consistent with stronger latent-space transport toward the target distribution. In contrast, enumeration-based baselines generally saturated within relatively local structural neighborhoods surrounding the originating linker scaffold. These results suggest that the latent-space transport formulation enables controllable exploration beyond local functional-group modification regimes typically accessible through direct enumeration approaches.

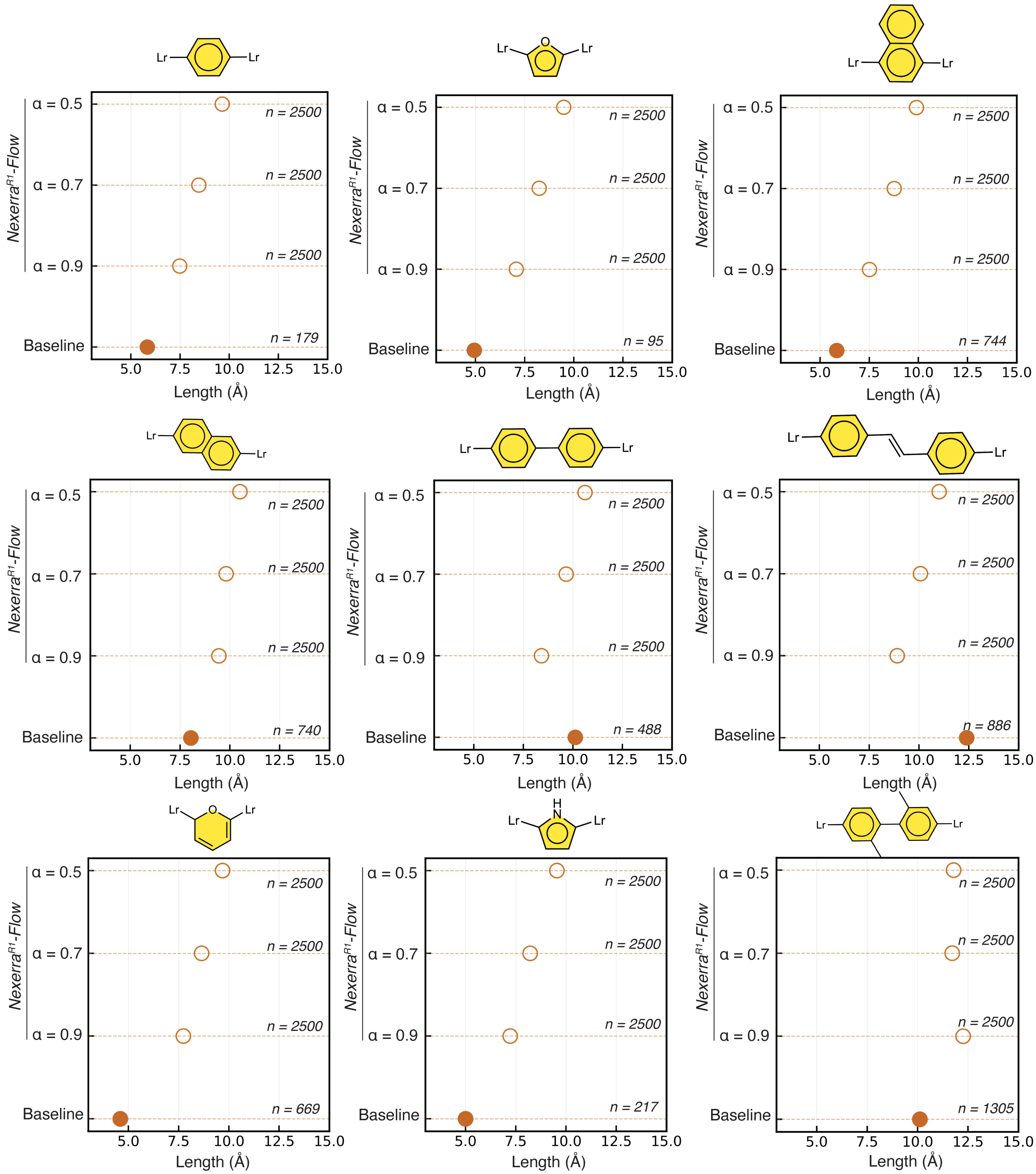

Figure S23. Comparison of random functionalization baseline and flow-guided sampling across representative linker seeds. For each seed, the filled marker denotes the mean linker length obtained from the local substitution baseline, while open markers denote the mean linker length obtained from the flow-guided model at different α values. All methods were assigned the same nominal sampling budget (2500 samples); the reported n values indicate the number of distinct candidates ultimately retained for each method. In many cases, the baseline saturates well below the nominal budget because only a limited number of unique valid local substitutions are reachable from the seed, whereas the flow-guided model continues to access a substantially larger candidate set. The dependence on α further shows that the flow model provides an explicit control over exploration distance from the seed, thereby modulating both accessible diversity and the resulting linker-length distribution.

When the input linker already lies near the upper tail of the span distribution, there is less available headroom for further improvement.

Thus, traditional linker diversification based on random, direct functionalization of the seed typically explores highly local chemical neighborhoods that surround the seed. While these approaches do generate chemically valid analogues, they often saturate rapidly in diversity and provide limited control over emergent properties. In contrast, Nexerra[R1]-Flow operates within the continuous latent manifold enabling controllable latent space traversal leading to the generation of diverse linker populations with controllable emergent properties (**Figure S24**).

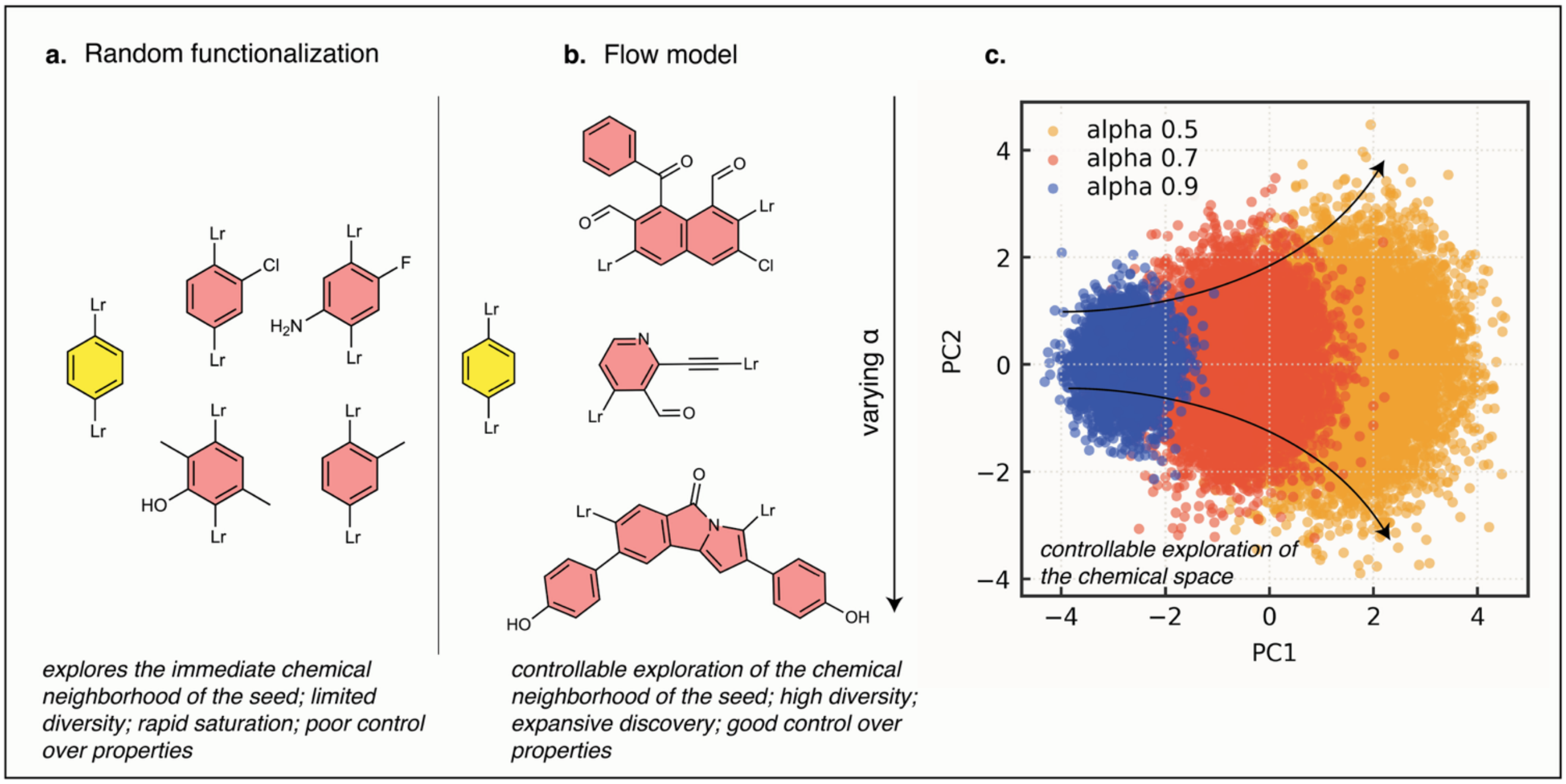

Figure S24. Conceptual comparison between random functionalization and flow-guided latent transport. **a.** Random functionalization explores highly local chemical neighborhood of the seed, resulting in a limited diversity and rapid saturation of linker populations. This ultimately results in poor control over emergent properties. **b.** In contrast, the flow-guided transport framework enables a controllable exploration of the chemical neighborhood of the seed, resulting in high diversity and expansive discovery, with good control over emergent properties. **c.** By controlling the α parameter, the model can achieve progressively stronger and more expansive latent space transport. The figure shows a PCA plot of linkers generated by Nexerra[R1]-Flow using the BDC seed at varying α values. The points are colored by the α value. (n = 9490, 8245, and 3637 at α = 0.5, 0.7, and 0.9 respectively; although each iteration was given the same sampling budget, higher α values produced fewer unique linkers).

S9.6. Rediscovery of experimentally reported linker families. To evaluate whether the flow model generates chemically realistic reticular linkers, we performed a rediscovery analysis using representative seed linkers frequently encountered in experimentally realized MOFs. Specifically, we selected BDC (UiO-66) and biphenyl-4,4'-dicarboxylic acid (BPDC; UiO-67) as representative seeds and generated linker populations using Nexerra[R1]-Flow under identical sampling budgets. Generated molecules were compared against a curated set of experimentally reported reticular linkers using Tanimoto similarity (based on Morgan fingerprints). Exact structural matches and chemically proximal analogues were subsequently identified. **Figure S25** summarizes representative examples obtained from the analysis. The flow model recovered several experimentally reported linker families exactly, including BDC, BPDC, TPDC (UiO-68), and DPA (DPA-MOF). In addition, the model

generated multiple structurally related analogues exhibiting substantial chemical similarity to experimentally reported linkers (defined as Tanimoto similarity > 0.5) while preserving key desirable characteristics including anchor symmetry, rigidity, and compatible linker geometries. Importantly, many of the generated analogues extend beyond simple substituent modification and explore chemically distinct yet assembly-compatible scaffold variations. These results suggest that the learned latent representations encode chemically meaningful transformations within reticular linker space and are capable of navigating toward experimentally relevant linker families while preserving reticular compatibility constraints.

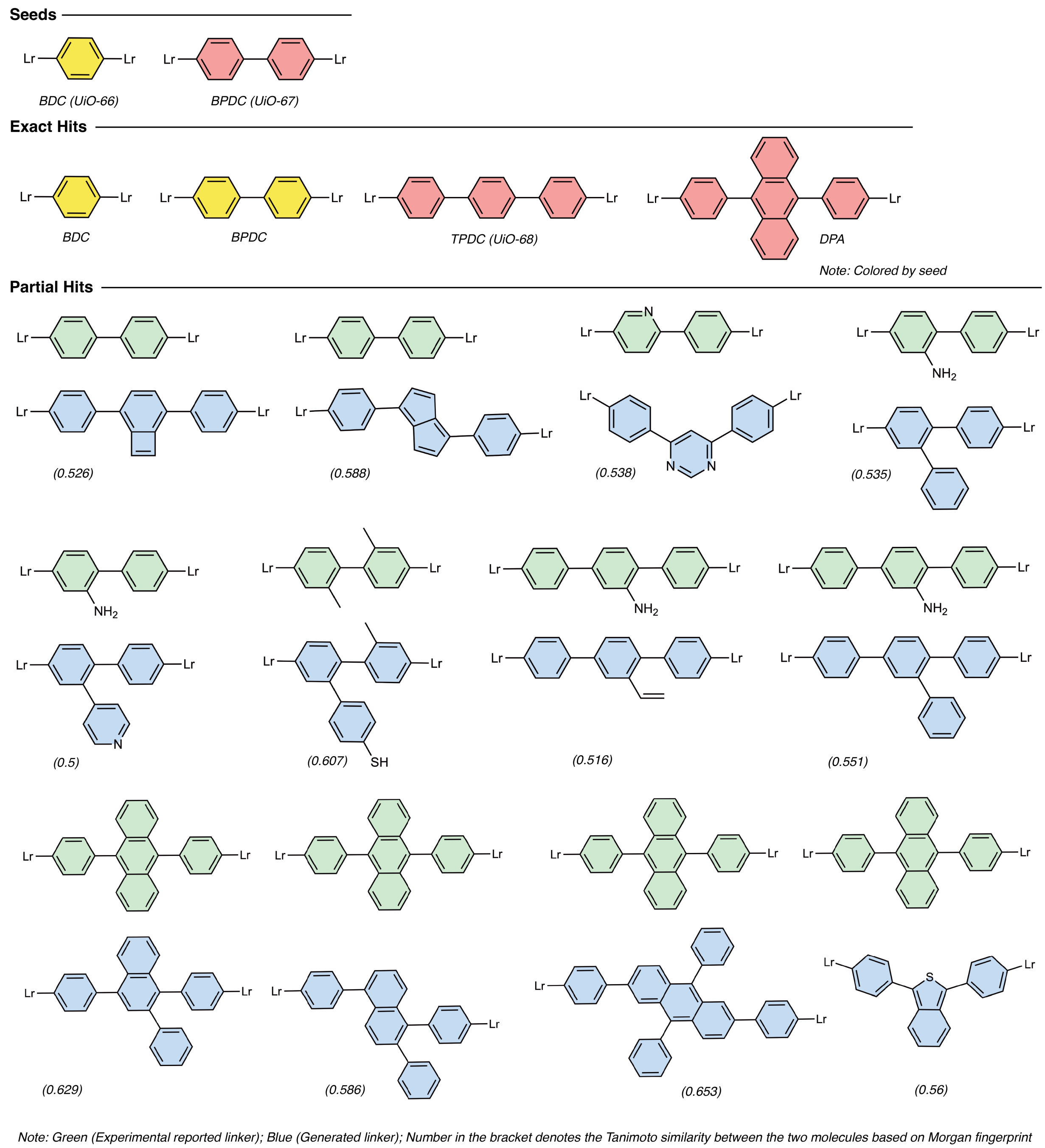

Figure S25. Starting from BDC and BPDC, Nexerra[R1]-Flow recovered several experimentally reported linker families exactly, including BDC, BPDC, TPDC (UiO-68), and DPA (DPA-MOF). The model additionally generated structurally similar analogues of several experimentally reported linkers, while preserving desirable characteristics including anchor

symmetry and compatible linker geometries. Green structures denote experimentally reported linkers; blue structures denote generated linkers. Values in parentheses indicate Morgan fingerprint Tanimoto similarity between generated and experimentally reported linker structures.

Section S10. Molecular Simulations

S10.1. Framework assembly. Structural models were assembled using the Topologically Based Crystal Constructor (ToBaCCo)[18], which constructs MOFs by embedding building blocks within pre-defined edge-transitive network topologies. The organic linker was generated using Nexerra[R1], while the SBU and target topology were specified *a priori* during assembly. Following assembly, structures with atomic overlaps were discarded. The remaining structures were subsequently geometry optimised using the Forcite module in Materials Studio.[34] Optimisation was performed using the SMART algorithm, which combines steepest descent, adjusted basis set Newton-Raphson, and quasi-Newton optimisation. Bonded and short-range non-bonded interactions were described using UFF,[35] while long-range electrostatics were treated using a Coulombic potential. A non-bonded interaction cutoff of 18.5 Å was used.

S10.2. Computational details. Gravimetric and volumetric methane uptake capacities were obtained from grand canonical Monte Carlo (GCMC) simulations, in which chemical potential, volume, and temperature were held constant. Simulations were performed with the RASPA code,[36] treating both framework and adsorbate molecules as rigid. During each Monte Carlo cycle, adsorbates were allowed to undergo translation, insertion, and swap moves – each with equal probability. Each simulation comprised 30,000 cycles; the first 15,000 cycles were treated as equilibration, following which ensemble averages were collected over 15,000 production cycles. The number of trial moves per cycle was either the current number of adsorbates in the system or 20, whichever is larger.

Adsorbate-framework and adsorbate-adsorbate interactions were described using Lennard-Jones (LJ) potentials combined with electrostatic interactions. LJ interactions were truncated and shifted to zero at 12.5 Å, and Ewald summation was applied for long-range electrostatics. LJ parameters for CH_4 were adopted from Martin, Thompson and Nenoff.[37] Framework LJ parameters were chosen from the Dreiding force field,[38] where available, and with UFF parameters otherwise.[35] No framework partial charges were assigned since the CH_4 model adopted does not include partial charges. For interactions lacking explicit parametrization, cross-interactions were determined using Lorentz-Berthelot mixing rules. We append a set of sample GCMC scripts in the source GitHub repository that supports this manuscript for readers who wish to reproduce these calculations.

S10.3. Comparative GCMC analysis of MOF-525 and CU-525. To evaluate whether the scaffold-constrained linker design method produces experimentally realizable frameworks with improved adsorption characteristics relative to the originating seed structure, we performed comparative CH_4 adsorption simulations on MOF-525 and CU-525 under identical conditions. Prior to GCMC simulations, both MOF-525 and CU-525 were geometry optimized using the SMART algorithm (discussed in **Section S10.1**). The experimental unit-cell parameters were held fixed during optimization, and the porphyrin cores were constrained to preserve planarity, while remaining degrees of freedom were allowed to relax. Simulations were performed at 298 K and 80 bar, using the same protocol described in **Section S10.2.** Under identical conditions, CU-525 exhibits a higher predicted gravimetric CH_4 uptake than MOF-525 (272 vs 218 mg g^{-1}) (**Figure S26**). The enhancement is consistent with the expanded pore architecture associated with the scaffold-constrained linker modification.

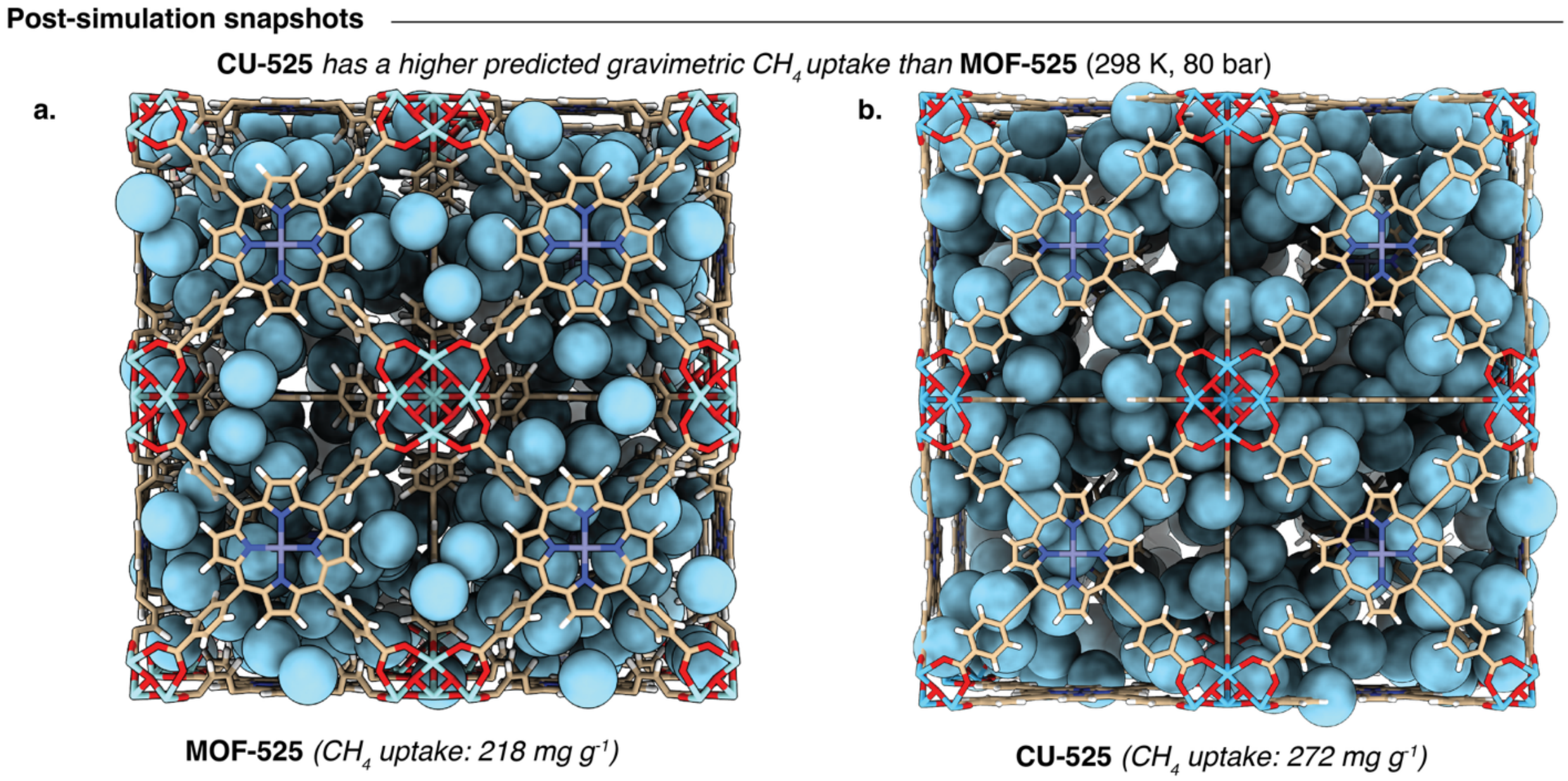

Figure S26. Post-simulation snapshots of CH_4 uptake in MOF-525 and CU-525. GCMC simulations predict higher gravimetric CH_4 capacity in CU-525 relative to MOF-525. These results serve as computational evidence in support of the linker design rationale using the gravimetric reward function R_{grav}. (Blue spheres represent CH_4).

Section S11. References

(1) Guillerm, V.; Jiang, H.; Alezi, D.; Alsadun, N.; Eddaoudi, M. From Elementary to Advanced Design of Functional Metal–Organic Frameworks: A User Guide to Deciphering the Reticular Chemistry Toolbox. *Adv. Mater.* **2025**, *37* (52), 2414153. https://doi.org/10.1002/adma.202414153.

(2) Jiang, H.; Alezi, D.; Eddaoudi, M. A Reticular Chemistry Guide for the Design of Periodic Solids. *Nat. Rev. Mater.* **2021**, *6* (6), 466–487. https://doi.org/10.1038/s41578-021-00287-y.

(3) Li, M.; Li, D.; O'Keeffe, M.; Yaghi, O. M. Topological Analysis of Metal–Organic Frameworks with Polytopic Linkers and/or Multiple Building Units and the Minimal Transitivity Principle. *Chem. Rev.* **2014**, *114* (2), 1343–1370. https://doi.org/10.1021/cr400392k.

(4) Guillerm, V.; Kim, D.; Eubank, J. F.; Luebke, R.; Liu, X.; Adil, K.; Lah, M. S.; Eddaoudi, M. A Supermolecular Building Approach for the Design and Construction of Metal–Organic Frameworks. *Chem Soc Rev* **2014**, *43* (16), 6141–6172. https://doi.org/10.1039/C4CS00135D.

(5) Jiang, W.; Liang, C.; Zhang, Y. Multivariate Metal–Organic Frameworks for Programming Functions. *Adv. Funct. Mater.* **2024**, *34* (43), 2308946. https://doi.org/10.1002/adfm.202308946.

(6) Jiang, H.; Benzaria, S.; Alsadun, N.; Jia, J.; Czaban-Jóźwiak, J.; Guillerm, V.; Shkurenko, A.; Thiam, Z.; Bonneau, M.; Maka, V. K.; Chen, Z.; Ameur, Z. O.; O'Keeffe, M.; Eddaoudi, M. Merged-Nets Enumeration for the Systematic Design of Multicomponent Reticular Structures. *Science* **2024**, *386* (6722), 659–666. https://doi.org/10.1126/science.ads7866.

(7) Chen, X.; Menon, D.; Wang, X.; He, M.; Kiapi, M. R. A.; Asgari, M.; Lyu, Y.; Tang, X.; Keenan, L. L.; Shepard, W.; Wee, L. H.; Yang, S.; Farha, O. K.; Fairen-Jimenez, D. Flexibility-Frustrated Porosity for Enhanced Selective CO2 Adsorption in an Ultramicroporous Metal-Organic Framework. *Chem* **2025**, *11* (7), 102382. https://doi.org/10.1016/j.chempr.2024.11.020.

(8) Gupta, M.; Vittal, J. J. Control of Interpenetration and Structural Transformations in the Interpenetrated MOFs. *Coord. Chem. Rev.* **2021**, *435*, 213789. https://doi.org/10.1016/j.ccr.2021.213789.

(9) Krenn, M.; Ai, Q.; Barthel, S.; Carson, N.; Frei, A.; Frey, N. C.; Friederich, P.; Gaudin, T.; Gayle, A. A.; Jablonka, K. M.; Lameiro, R. F.; Lemm, D.; Lo, A.; Moosavi, S. M.; Nápoles-Duarte, J. M.; Nigam, A.; Pollice, R.; Rajan, K.; Schatzschneider, U.; Schwaller, P.; Skreta, M.; Smit, B.; Strieth-Kalthoff, F.; Sun, C.; Tom, G.; Falk Von Rudorff, G.; Wang, A.; White, A. D.; Young, A.; Yu, R.; Aspuru-Guzik, A. SELFIES and the Future of Molecular String Representations. *Patterns* **2022**, *3* (10), 100588. https://doi.org/10.1016/j.patter.2022.100588.

(10) Krenn, M.; Häse, F.; Nigam, A.; Friederich, P.; Aspuru-Guzik, A. Self-Referencing Embedded Strings (SELFIES): A 100% Robust Molecular String Representation. *Mach. Learn. Sci. Technol.* **2020**, *1* (4), 045024. https://doi.org/10.1088/2632-2153/aba947.
(11) Kingma, D. P.; Welling, M. Auto-Encoding Variational Bayes. arXiv December 10, 2022. https://doi.org/10.48550/arXiv.1312.6114.
(12) Vaswani, A.; Shazeer, N.; Parmar, N.; Uszkoreit, J.; Jones, L.; Gomez, A. N.; Kaiser, L.; Polosukhin, I. Attention Is All You Need. arXiv August 2, 2023. https://doi.org/10.48550/arXiv.1706.03762.
(13) Holtzman, A.; Buys, J.; Du, L.; Forbes, M.; Choi, Y. The Curious Case of Neural Text Degeneration. arXiv February 14, 2020. https://doi.org/10.48550/arXiv.1904.09751.
(14) Frey, N. C.; Soklaski, R.; Axelrod, S.; Samsi, S.; Gómez-Bombarelli, R.; Coley, C. W.; Gadepally, V. Neural Scaling of Deep Chemical Models. *Nat. Mach. Intell.* **2023**, *5* (11), 1297–1305. https://doi.org/10.1038/s42256-023-00740-3.
(15) Skinnider, M. A.; Stacey, R. G.; Wishart, D. S.; Foster, L. J. Chemical Language Models Enable Navigation in Sparsely Populated Chemical Space. *Nat. Mach. Intell.* **2021**, *3* (9), 759–770. https://doi.org/10.1038/s42256-021-00368-1.
(16) Yao, Z.; Sánchez-Lengeling, B.; Bobbitt, N. S.; Bucior, B. J.; Kumar, S. G. H.; Collins, S. P.; Burns, T.; Woo, T. K.; Farha, O. K.; Snurr, R. Q.; Aspuru-Guzik, A. Inverse Design of Nanoporous Crystalline Reticular Materials with Deep Generative Models. *Nat. Mach. Intell.* **2021**, *3* (1), 76–86. https://doi.org/10.1038/s42256-020-00271-1.
(17) RDKit: Open-Source Cheminformatics Software. https://www.rdkit.org (accessed 2026-03-02).
(18) Colón, Y. J.; Gómez-Gualdrón, D. A.; Snurr, R. Q. Topologically Guided, Automated Construction of Metal–Organic Frameworks and Their Evaluation for Energy-Related Applications. *Cryst. Growth Des.* **2017**, *17* (11), 5801–5810. https://doi.org/10.1021/acs.cgd.7b00848.
(19) Chen, Z.; Li, P.; Anderson, R.; Wang, X.; Zhang, X.; Robison, L.; Redfern, L. R.; Moribe, S.; Islamoglu, T.; Gómez-Gualdrón, D. A.; Yildirim, T.; Stoddart, J. F.; Farha, O. K. Balancing Volumetric and Gravimetric Uptake in Highly Porous Materials for Clean Energy. *Science* **2020**, *368* (6488), 297–303. https://doi.org/10.1126/science.aaz8881.
(20) Chen, X.; Wee, L. H.; Alizadeh Kiapi, M. R.; Tampaxis, C.; Zheng, Y.; Asgari, M.; Menon, D.; Willhammar, T.; Keenan, L.; Charalambopoulou, G.; Cui, Y.; Steriotis, T.; Fairen-Jimenez, D. Topology-Driven Design of an Ultraporous Red MOF for High-Performance Hydrogen Storage. September 24, 2025. https://doi.org/10.26434/chemrxiv-2025-f39b4.
(21) Alezi, D.; Belmabkhout, Y.; Suyetin, M.; Bhatt, P. M.; Weseliński, Ł. J.; Solovyeva, V.; Adil, K.; Spanopoulos, I.; Trikalitis, P. N.; Emwas, A.-H.; Eddaoudi, M. MOF Crystal Chemistry Paving the Way to Gas Storage Needs: Aluminum-Based **Soc** -MOF for CH_4 , O_2 , and CO_2 Storage. *J. Am. Chem. Soc.* **2015**, *137* (41), 13308–13318. https://doi.org/10.1021/jacs.5b07053.
(22) Menon, D.; Fairen-Jimenez, D. Guiding the Rational Design of Biocompatible Metal-Organic Frameworks for Drug Delivery. *Matter* **2025**, 101958. https://doi.org/10.1016/j.matt.2025.101958.
(23) Wu, L.; Yan, B.; Han, J.; Li, R.; Xiao, J.; He, S.; Bo, X. TOXRIC: A Comprehensive Database of Toxicological Data and Benchmarks. *Nucleic Acids Res.* **2023**, *51* (D1), D1432–D1445. https://doi.org/10.1093/nar/gkac1074.
(24) Matsunaga, S.; Endo, N.; Mori, W. A New Metal Carboxylate Framework Based on Porphyrin with Extended π-Conjugation. *Eur. J. Inorg. Chem.* **2011**, *2011* (29), 4550–4557. https://doi.org/10.1002/ejic.201100660.
(25) Winter, G. *Xia2* : An Expert System for Macromolecular Crystallography Data Reduction. *J. Appl. Crystallogr.* **2010**, *43* (1), 186–190. https://doi.org/10.1107/S0021889809045701.
(26) Sheldrick, G. M. *SHELXT* – Integrated Space-Group and Crystal-Structure Determination. *Acta Crystallogr. Sect. Found. Adv.* **2015**, *71* (1), 3–8. https://doi.org/10.1107/S2053273314026370.
(27) Sheldrick, G. M. Crystal Structure Refinement with *SHELXL*. *Acta Crystallogr. Sect. C Struct. Chem.* **2015**, *71* (1), 3–8. https://doi.org/10.1107/S2053229614024218.
(28) Dolomanov, O. V.; Bourhis, L. J.; Gildea, R. J.; Howard, J. A. K.; Puschmann, H. *OLEX2* : A Complete Structure Solution, Refinement and Analysis Program. *J. Appl. Crystallogr.* **2009**, *42* (2), 339–341. https://doi.org/10.1107/S0021889808042726.
(29) Lipman, Y.; Chen, R. T. Q.; Ben-Hamu, H.; Nickel, M.; Le, M. Flow Matching for Generative Modeling. arXiv February 8, 2023. https://doi.org/10.48550/arXiv.2210.02747.
(30) Tong, A.; Fatras, K.; Malkin, N.; Huguet, G.; Zhang, Y.; Rector-Brooks, J.; Wolf, G.; Bengio, Y. Improving and Generalizing Flow-Based Generative Models with Minibatch Optimal Transport. arXiv March 11, 2024. https://doi.org/10.48550/arXiv.2302.00482.
(31) Ho, J.; Salimans, T. Classifier-Free Diffusion Guidance. arXiv July 26, 2022. https://doi.org/10.48550/arXiv.2207.12598.
(32) He, K.; Zhang, X.; Ren, S.; Sun, J. Deep Residual Learning for Image Recognition. arXiv December 10, 2015. https://doi.org/10.48550/arXiv.1512.03385.
(33) Perez, E.; Strub, F.; Vries, H. de; Dumoulin, V.; Courville, A. FiLM: Visual Reasoning with a General Conditioning Layer. arXiv December 18, 2017. https://doi.org/10.48550/arXiv.1709.07871.
(34) Dassault Systèmes. BIOVIA Materials Studio 7.0, 2013.

(35) Rappe, A. K.; Casewit, C. J.; Colwell, K. S.; Goddard, W. A.; Skiff, W. M. UFF, a Full Periodic Table Force Field for Molecular Mechanics and Molecular Dynamics Simulations. *J. Am. Chem. Soc.* **1992**, *114* (25), 10024–10035. https://doi.org/10.1021/ja00051a040.
(36) Dubbeldam, D.; Calero, S.; Ellis, D. E.; Snurr, R. Q. RASPA: Molecular Simulation Software for Adsorption and Diffusion in Flexible Nanoporous Materials. *Mol. Simul.* **2016**, *42* (2), 81–101. https://doi.org/10.1080/08927022.2015.1010082.
(37) Martin, M. G.; Thompson, A. P.; Nenoff, T. M. Effect of Pressure, Membrane Thickness, and Placement of Control Volumes on the Flux of Methane through Thin Silicalite Membranes: A Dual Control Volume Grand Canonical Molecular Dynamics Study. *J. Chem. Phys.* **2001**, *114* (16), 7174–7181. https://doi.org/10.1063/1.1360256.
(38) Mayo, S. L.; Olafson, B. D.; Goddard, W. A. DREIDING: A Generic Force Field for Molecular Simulations. *J. Phys. Chem.* **1990**, *94* (26), 8897–8909. https://doi.org/10.1021/j100389a010.
